# Supplementary material for: Synthesis, Properties, and Biological Activity Evaluation of Some Novel Naphtho[2,1-e]pyrazolo[5,1-c][1,2,4]triazines
Source: Int J Mol Sci. 2025 Aug 8;26(16):7681. doi: 10.3390/ijms26167681 (PMC12386420; doi:10.3390/ijms26167681)
Supplement: Supplementary file 1 [file ijms-26-07681-s001.zip › ijms-3810669-supplementary.pdf]

Supplementary information

Contents

1D and 2D NMR spectra for compounds (3a), (3b), (4a), (4b), (5), (6) 4
IR spectra for compounds (3a), (3b), (4a), (4b), (5), (6)........ 24
UV-Vis spectra for compounds (3a), (3b), (4a), (4b), (5), (6)........ 30
Emission spectra for compounds (6) and (4b) 36
LC-HRMS data for compounds (3a), (3b), (4a), (4b), 5, 6 38
Biological activity evaluation data for compounds (3a), (3b), (4a), (4b), 5, 6........ 44

Figures

Figure S1 ^{1}H NMR spectrum of the compound **(3a)**......4
Figure S2 ^{13}C NMR spectrum of the compound **(3a)**......4
Figure S3 COSY ^{1}H-^{1}H spectrum of the compound **(3a)**......5
Figure S4 HSQC ^{1}H-^{13}C spectrum of the compound **(3a)**......5
Figure S5 HMBC ^{1}H-^{13}C spectrum of the compound **(3a)**......6
Figure S6 ^{1}H spectrum of the compound **(3b)**......6
Figure S7 ^{13}C NMR spectrum of the compound **(3b)**......7
Figure S8 COSY ^{1}H-^{1}H spectrum of the compound **(3b)**......7
Figure S9 ^{13}C DEPT 135 spectrum of the compound **(3b)**......8
Figure S10 ^{1}H-^{13}C HSQC spectrum of the compound **(3b)**......8
Figure S11 ^{1}H-^{15}N HSQC spectrum of the compound **(3b)**......9
Figure S12 ^{1}H-^{13}C HMBC spectrum of the compound **(3b)**......9
Figure S13 ^{1}H-^{15}N HMBC spectrum of the compound **(3b)**......10
Figure S14 ^{1}H NMR spectrum of the compound **(4a)**......10
Figure S15 ^{13}C NMR spectrum of the compound **(4a)**......11
Figure S16 ^{13}C DEPT 135 spectrum of the compound **(4a)**......11
Figure S17 ^{1}H-^{1}H COSY spectrum of the compound **(4a)**......12
Figure S18 ^{1}H-^{13}C HSQC spectrum of the compound **(4a)**......12
Figure S19 ^{1}H-^{13}C HMBC spectrum of the compound **(4a)**......13
Figure S20 ^{1}H-^{15}N spectrum of the compound **(4a)**......13
Figure S21 ^{1}H NMR spectrum of the compound **(4b)**......14
Figure S22 ^{13}C NMR spectrum of the compound **(4b)**......14
Figure S23 ^{13}C DEPT135 spectrum of the compound **(4b)**......15
Figure S24 ^{1}H-^{1}H COSY spectrum of the compound **(4b)**......15
Figure S25 ^{1}H-^{13}C HSQC spectrum of the compound **(4b)**......16
Figure S26 ^{1}H-^{13}C HMBC spectrum of the compound **(4b)**......16
Figure S27 ^{1}H-^{15}N HMBC spectrum of the compound **(4b)**......17
Figure S28 ^{1}H NMR spectrum of the compound **(5)**......17
Figure S29 ^{13}C NMR spectrum of the compound **(5)**......18
Figure S30 ^{13}C DEPT135 spectrum of the compound **(5)**......18
Figure S31 ^{1}H-^{1}H COSY spectrum of the compound **(5)**......19
Figure S32 ^{1}H-^{13}C HSQC spectrum of the compound **(5)**......19
Figure S33 ^{1}H-^{13}C HMBC spectrum of the compound **(5)**......20
Figure S34 ^{1}H NMR spectrum of the compound **(6)**......20
Figure S35 ^{13}C NMR spectrum of the compound **(6)**......21
Figure S36 ^{13}C DEPT135 spectrum of the compound **(6)**......21
Figure S37 ^{1}H-^{1}H COSY spectrum of the compound **(6)**......22
Figure S38 ^{1}H-^{13}C HSQC spectrum of the compound **(6)**......22
Figure S39 ^{1}H-^{13}C HMBC spectrum of the compound **(6)**......23
Figure S40 ^{1}H-^{15}N HMBC spectrum of the compound **(6)**......23
Figure S41 IR spectrum of the compound **(3a)**......24
Figure S42 IR spectrum of the compound **(3b)**......25

Figure S43 IR spectrum of the compound **(4a)** ... 26
Figure S44 IR spectrum of the compound **(4b)** ... 27
Figure S45 IR spectrum of the compound **(5)** ... 28
Figure S46 IR spectrum of the compound **(6)** ... 29
Figure S47 UV-Vis spectrum of the compound **(3a)** ... 30
Figure S48 UV-Vis spectrum of the compound **(3b)** ... 31
Figure S49 Measured (top) and calculated (bottom) UV-Vis spectra of the compound **(4a)** ... 32
Figure S50 Measured (top) and calculated (bottom) UV-Vis spectra of the compound **(4b)** ... 33
Figure S51 Measured (top) and calculated (bottom) UV-Vis spectra of the compound **(5)** ... 34
Figure S52 Measured (top) and calculated (bottom) UV-Vis spectra of the compound **(6)** ... 35
Figure S53 Emission spectrum of the compound **(6)** ... 36
Figure S54 Emission spectrum of the compound **(4b)** ... 37
Figure S55 LC-HRMS data for compound **(3a)** ... 38
Figure S56 LC-HRMS data for compound **(3b)** ... 39
Figure S57 LC-HRMS data for compound **(4a)** ... 40
Figure S58 LC-HRMS data for compound **(4b)** ... 41
Figure 59 HRMS spectrum of compound **(5)** ... 42
Figure S60 LC-HRMS data for compound **(6)** ... 43
Figure S61 Best ligand **(3a)** conformation (left) and ligand **(3a)** – receptor (3GCW protein) interaction ... 55
Figure S62 Depiction of the hydrogen bond established between the naphtol -OH group of compound **(3a)** and Asp360 residue ... 55
Figure S63 Best ligand **(3b)** conformation (left) and ligand **(3b)** – receptor (3GCW protein) interactions (right) ... 56
Figure S64 Best ligand **(4a)** conformation (left) and ligand **(4a)** – receptor (3GCW protein) interactions (right) ... 56
Figure S65 Best ligand **(4b)** conformation (left) and ligand **(4b)** – receptor (3GCW protein) interactions ... 56
Figure S66 Best ligand **(5)** conformation (left) and ligand **(5)** – receptor (3GCW protein) interactions (right) ... 57
Figure S67 Best ligand **(6)** conformation (left) and ligand **(6)** – receptor (3GCW protein) interactions (right) ... 57
Figure S68 Depiction of the hydrogen bond established between the naphtol -OH group of compound **(6)** and Glu426 residue ... 58

1D and 2D NMR spectra for compounds (3a), (3b), (4a), (4b), (5), (6)

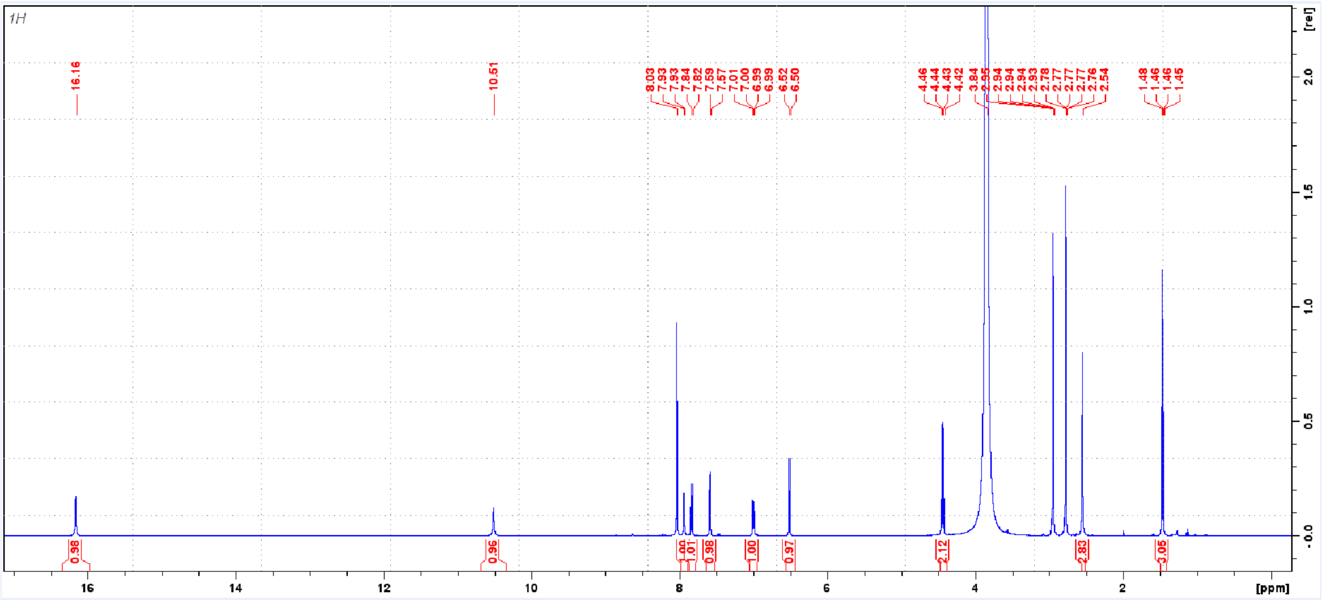

Figure S1 ^{1}H NMR spectrum of the compound **(3a)**

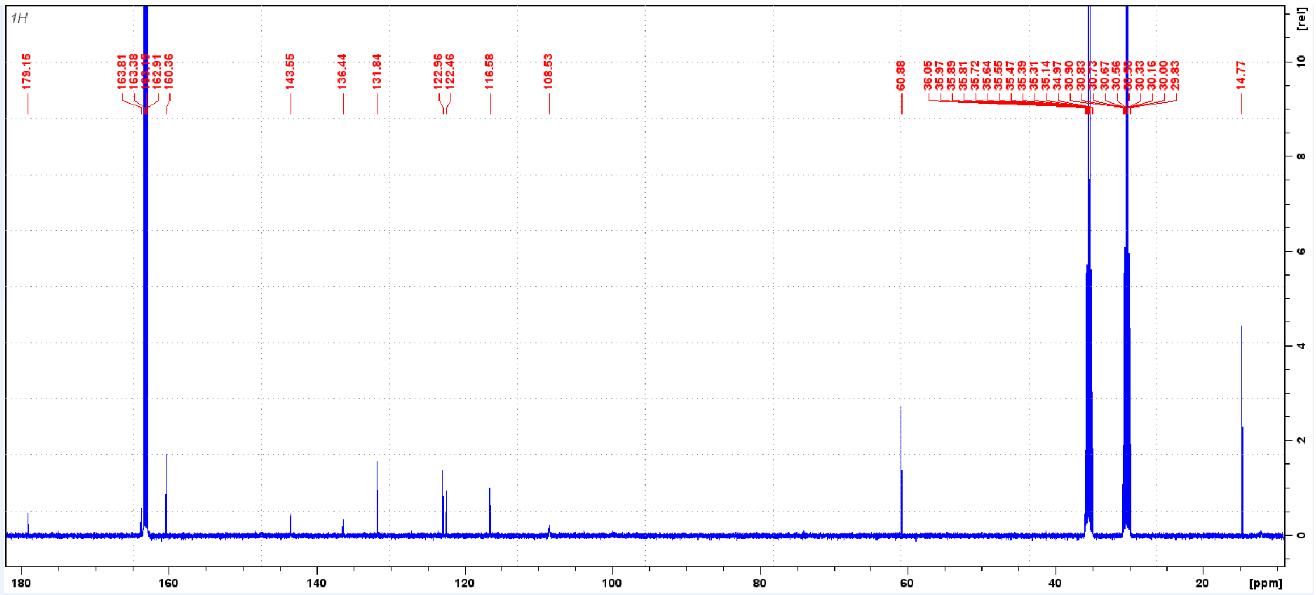

Figure S2 ^{13}C NMR spectrum of the compound **(3a)**

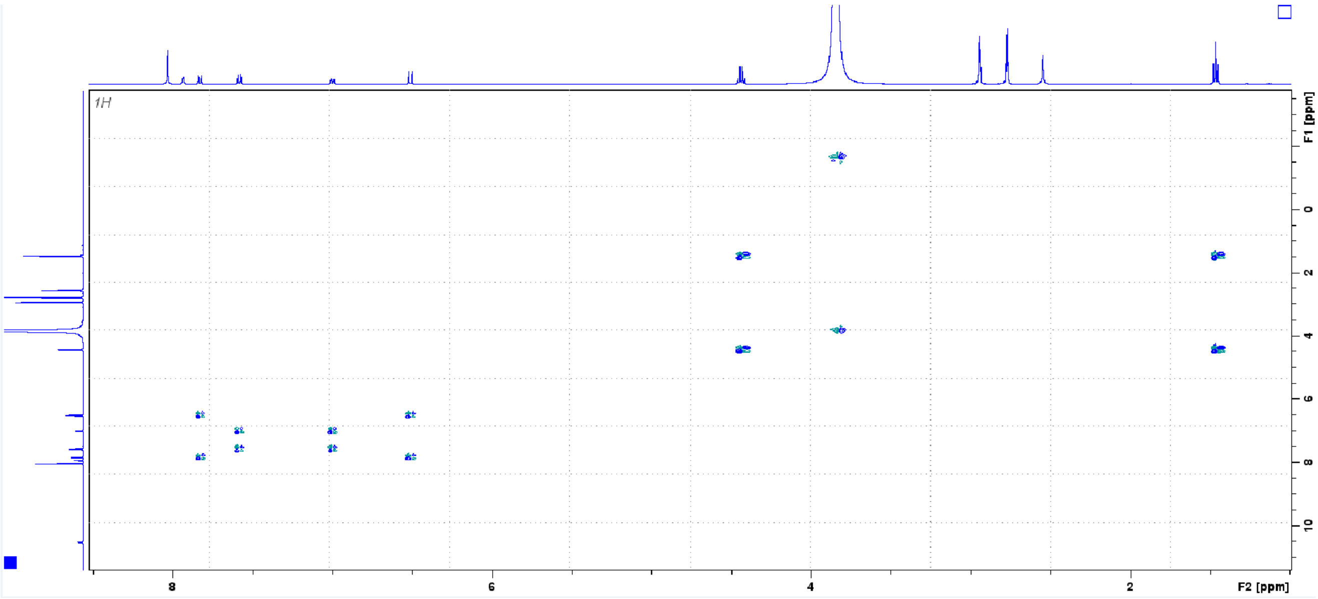

Figure S3 COSY ^{1}H-^{1}H spectrum of the compound **(3a)**

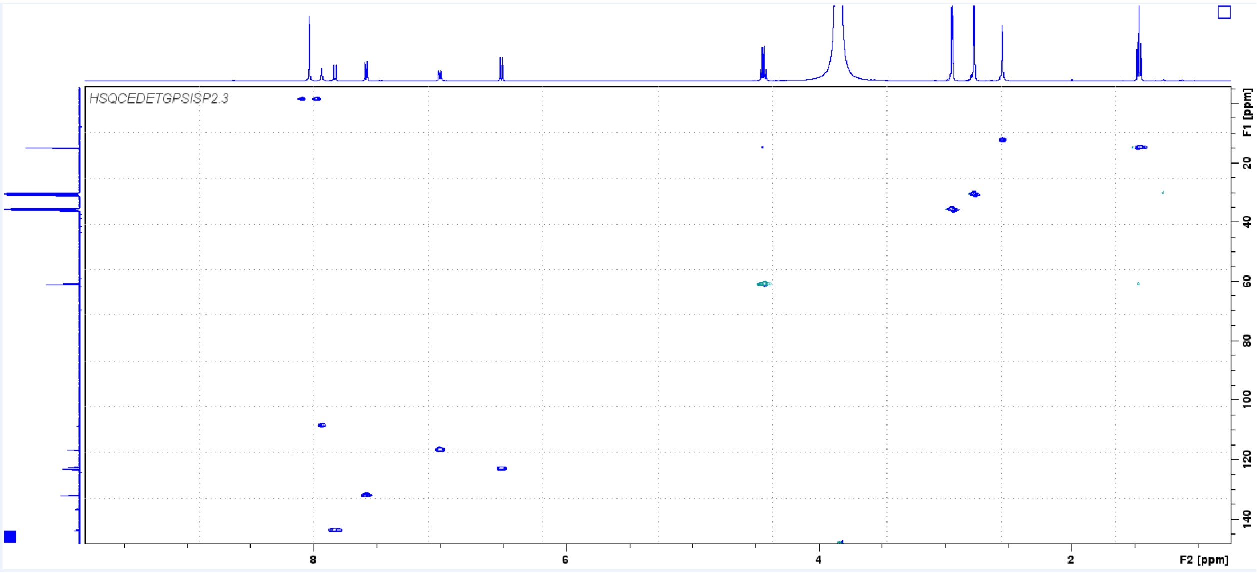

Figure S4 HSQC ^{1}H-^{13}C spectrum of the compound **(3a)**

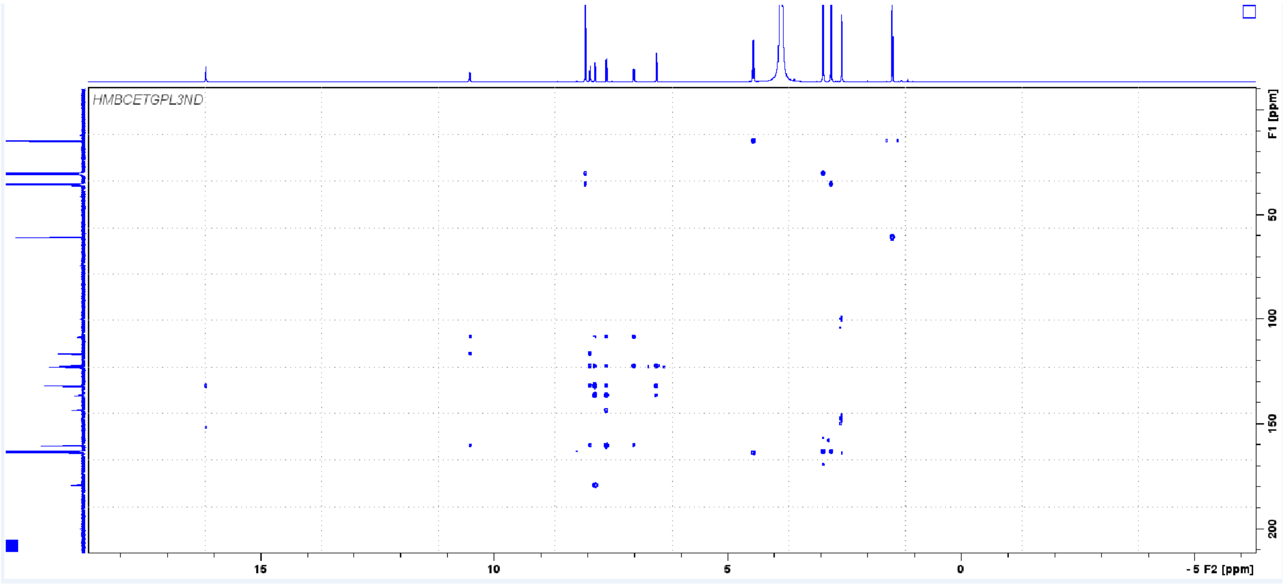

Figure S5 HMBC ^{1}H-^{13}C spectrum of the compound **(3a)**

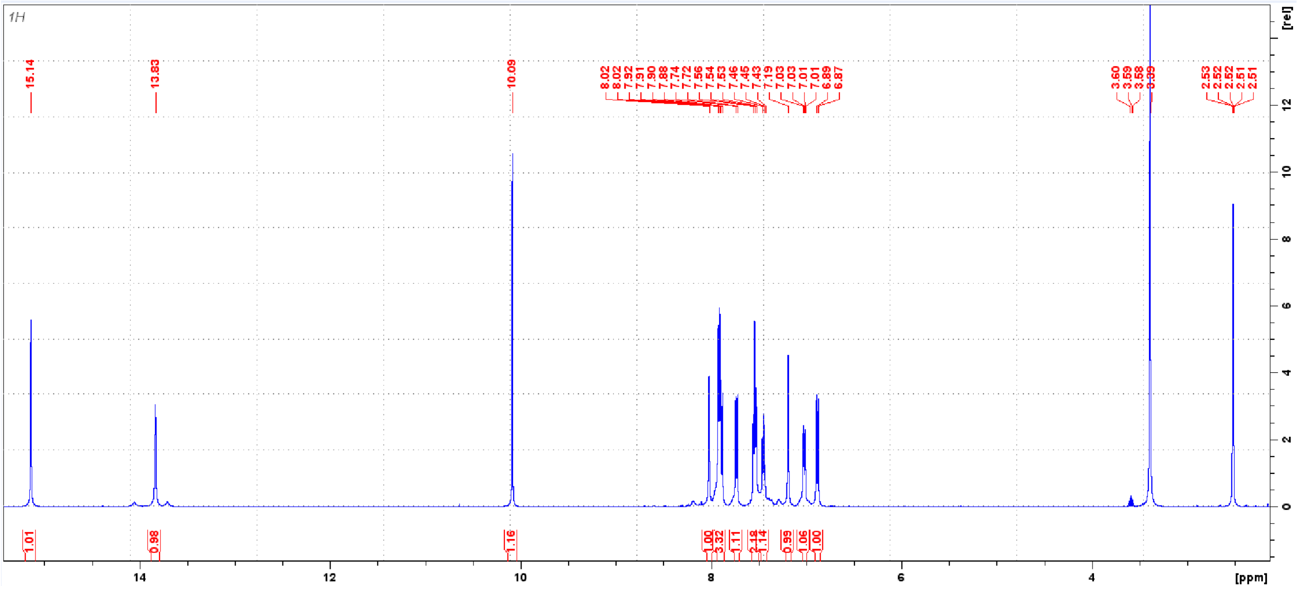

Figure S6 ^{1}H spectrum of the compound **(3b)**

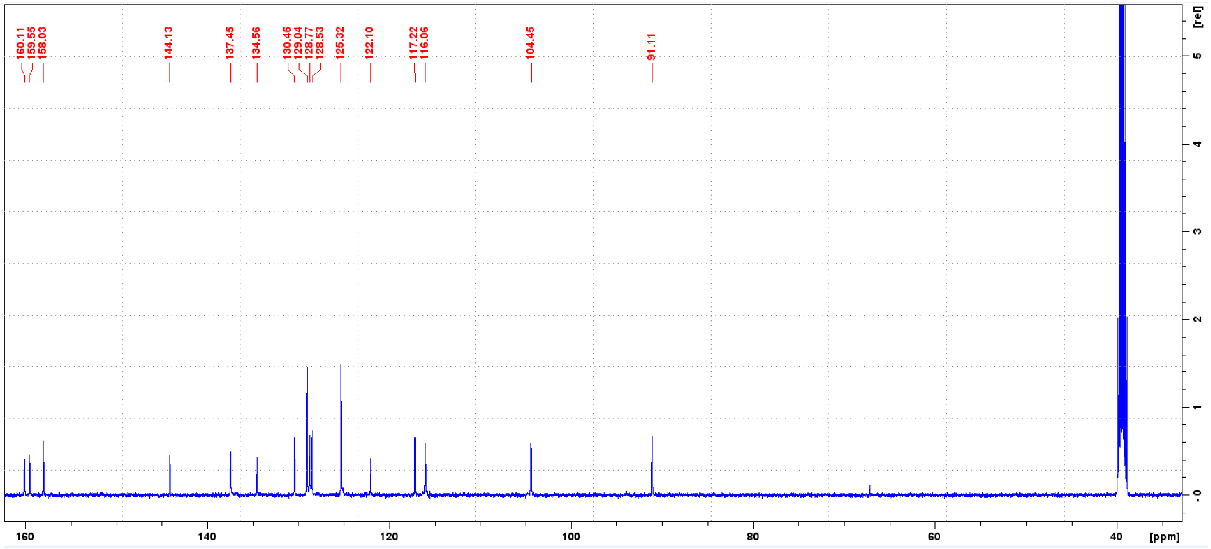

Figure S7 ^{13}C NMR spectrum of the compound **(3b)**

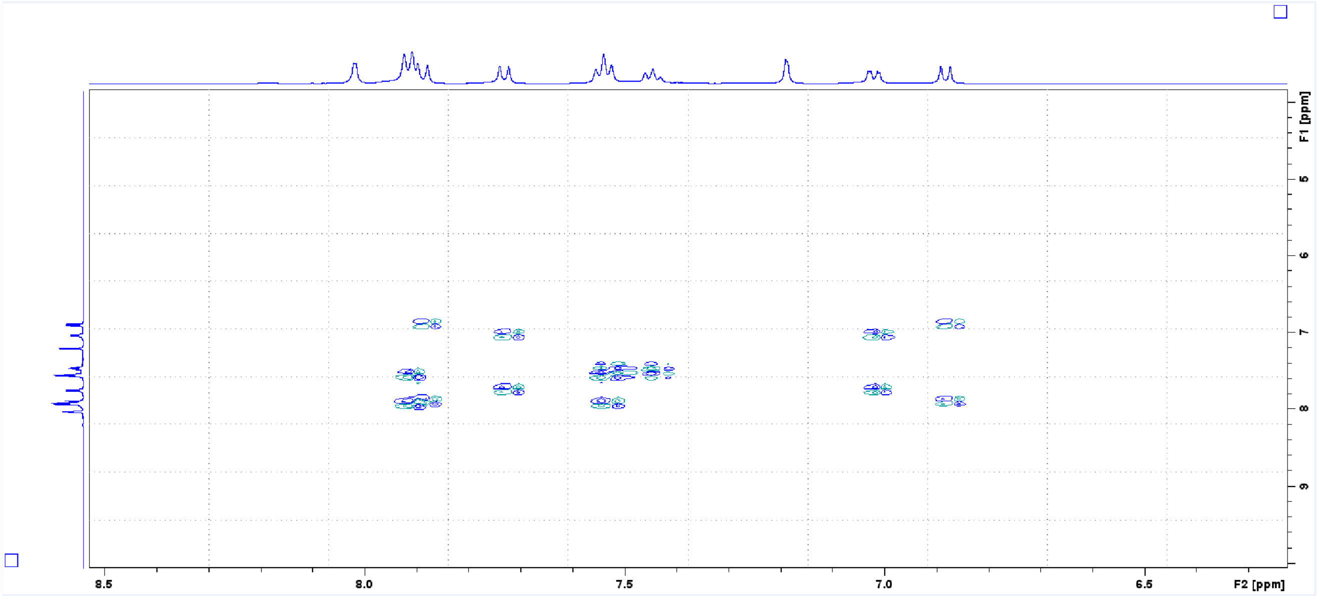

Figure S8 COSY ^{1}H-^{1}H spectrum of the compound **(3b)**

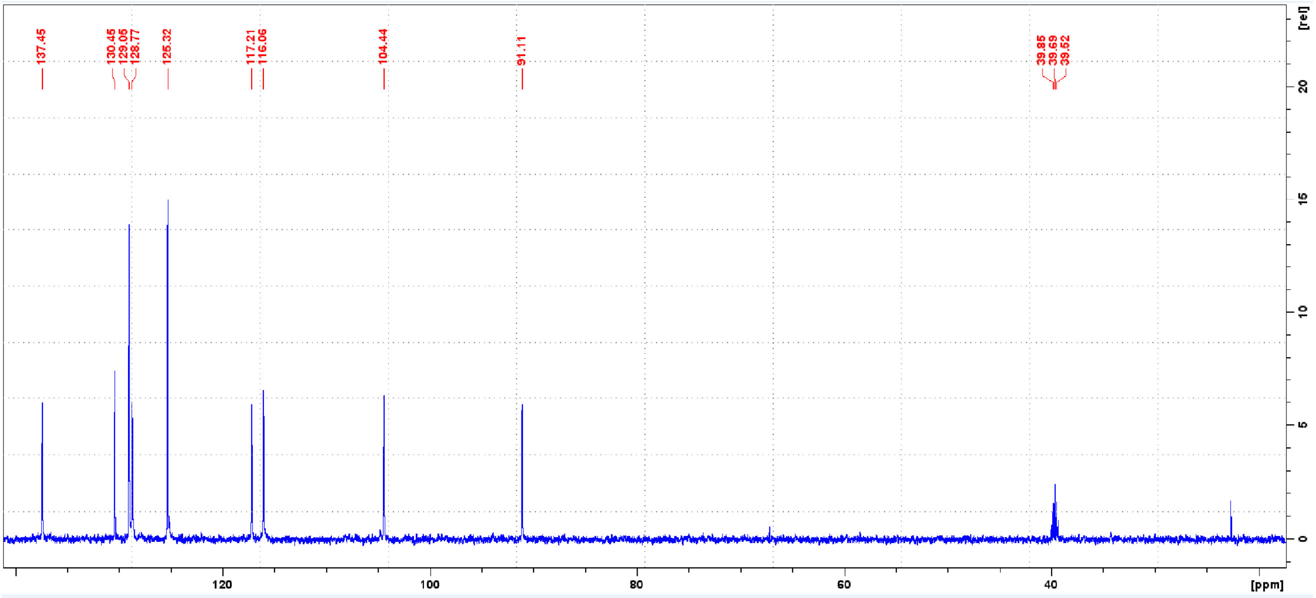

Figure S9 ^{13}C DEPT 135 spectrum of the compound **(3b)**

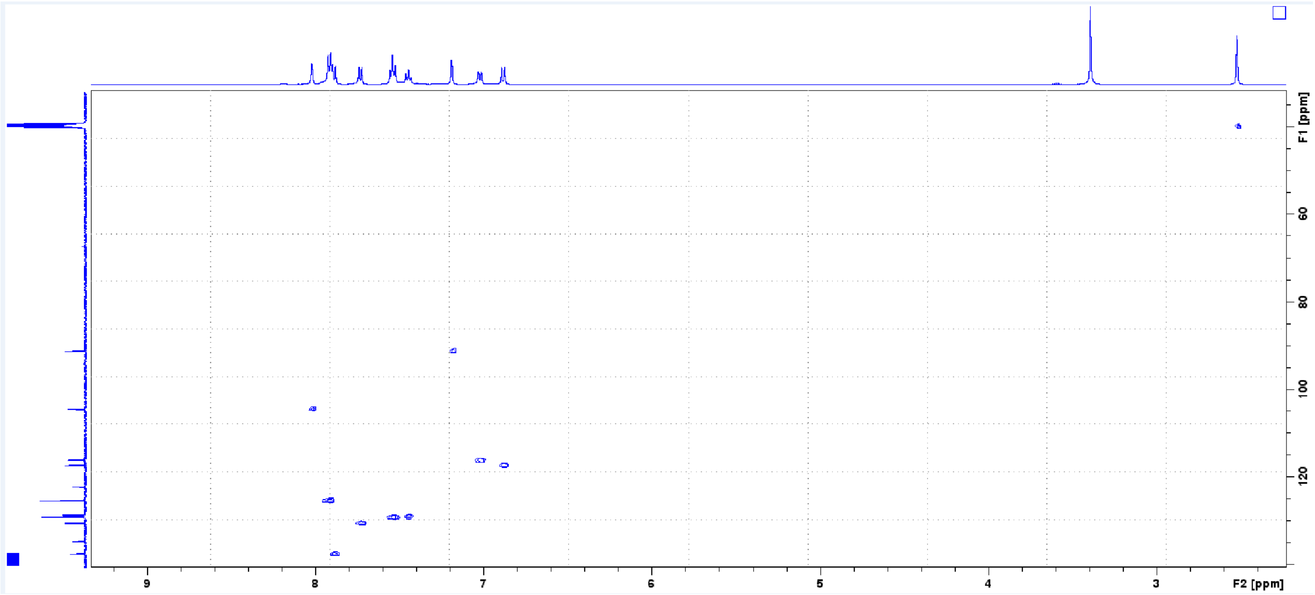

Figure S10 ^{1}H-^{13}C HSQC spectrum of the compound **(3b)**

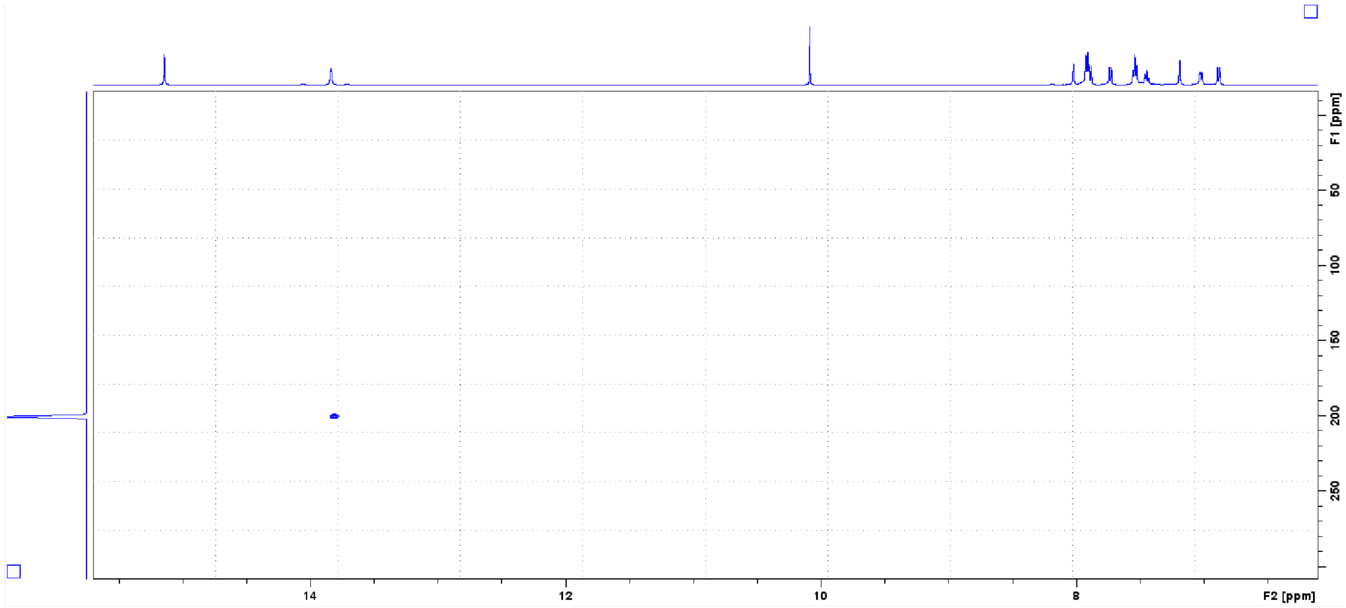

Figure S11 ^{1}H-^{15}N HSQC spectrum of the compound **(3b)**

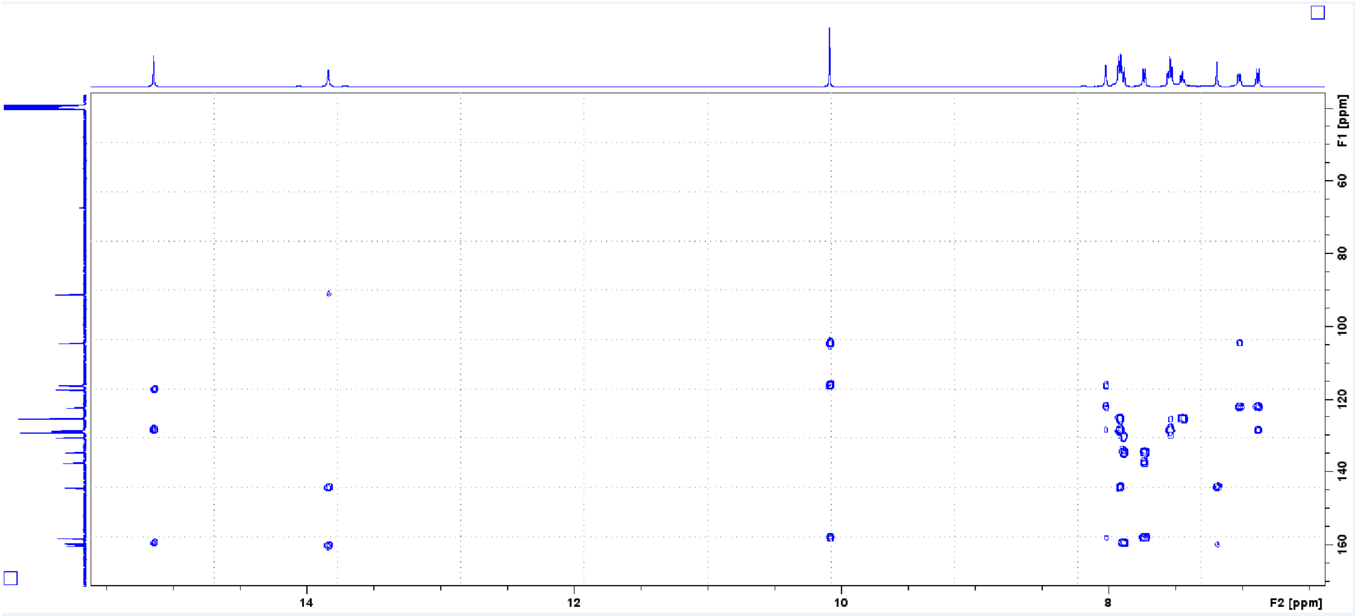

Figure S12 ^{1}H-^{13}C HMBC spectrum of the compound **(3b)**

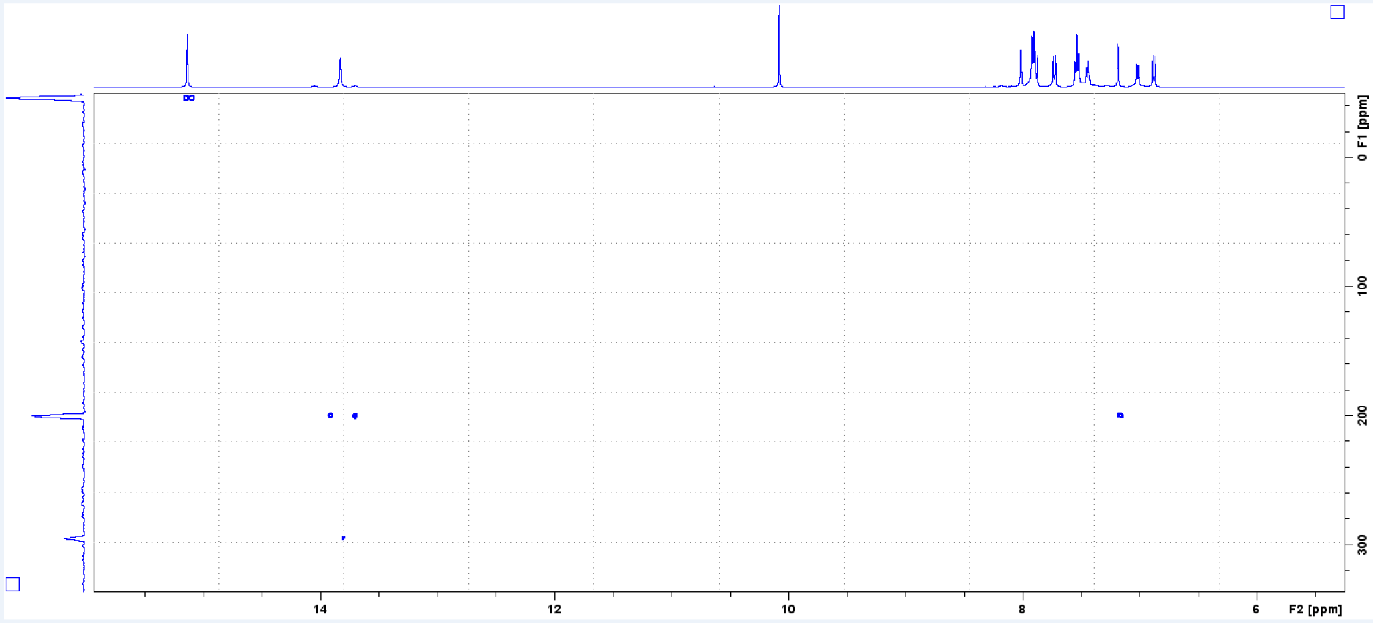

Figure S13 ^{1}H-^{15}N HMBC spectrum of the compound **(3b)**

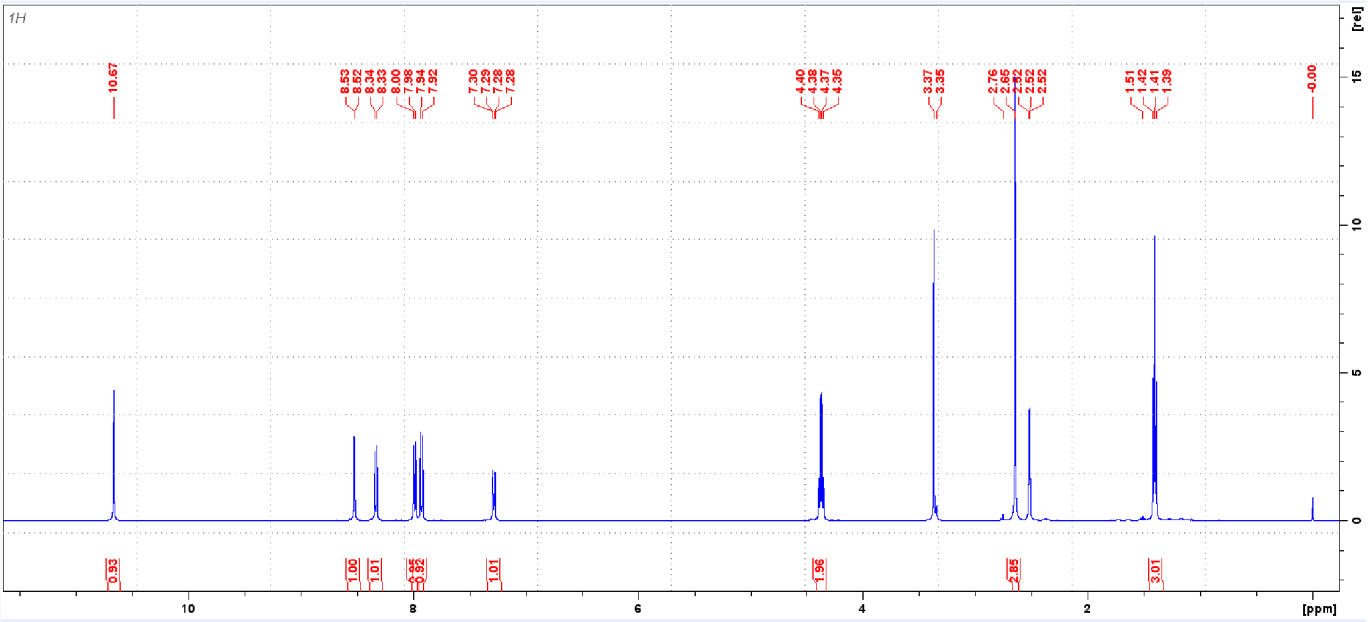

Figure S14 ^{1}H NMR spectrum of the compound **(4a)**

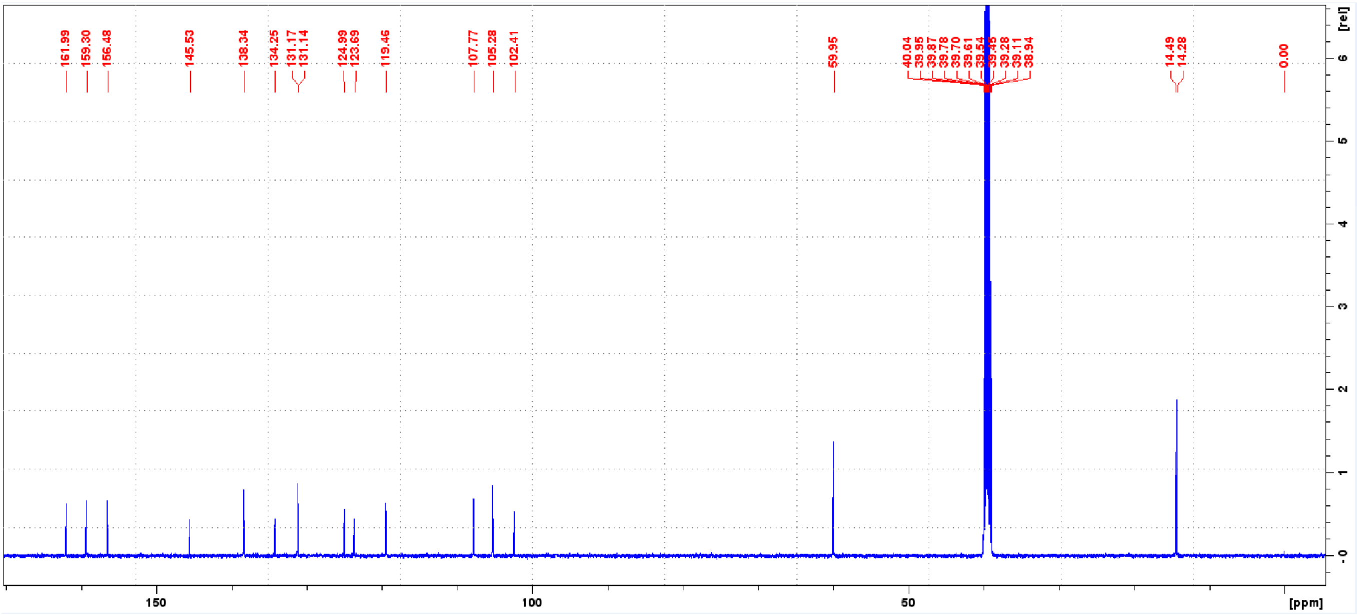

Figure S15 ^{13}C NMR spectrum of the compound **(4a)**

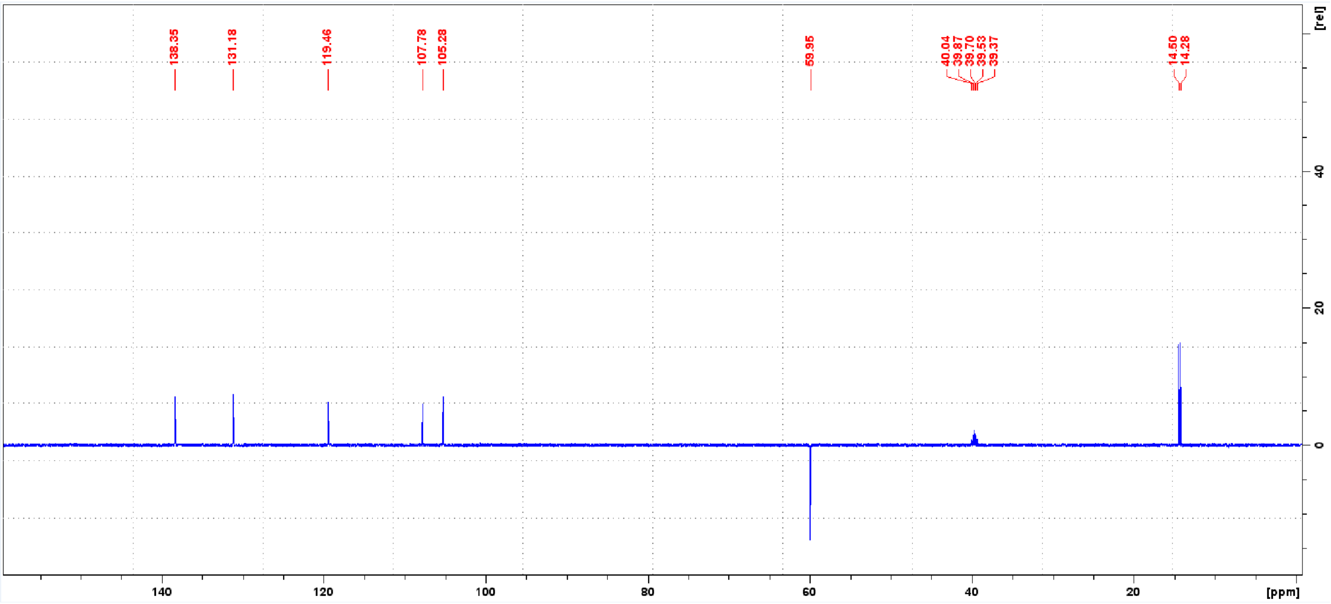

Figure S16 ^{13}C DEPT 135 spectrum of the compound **(4a)**

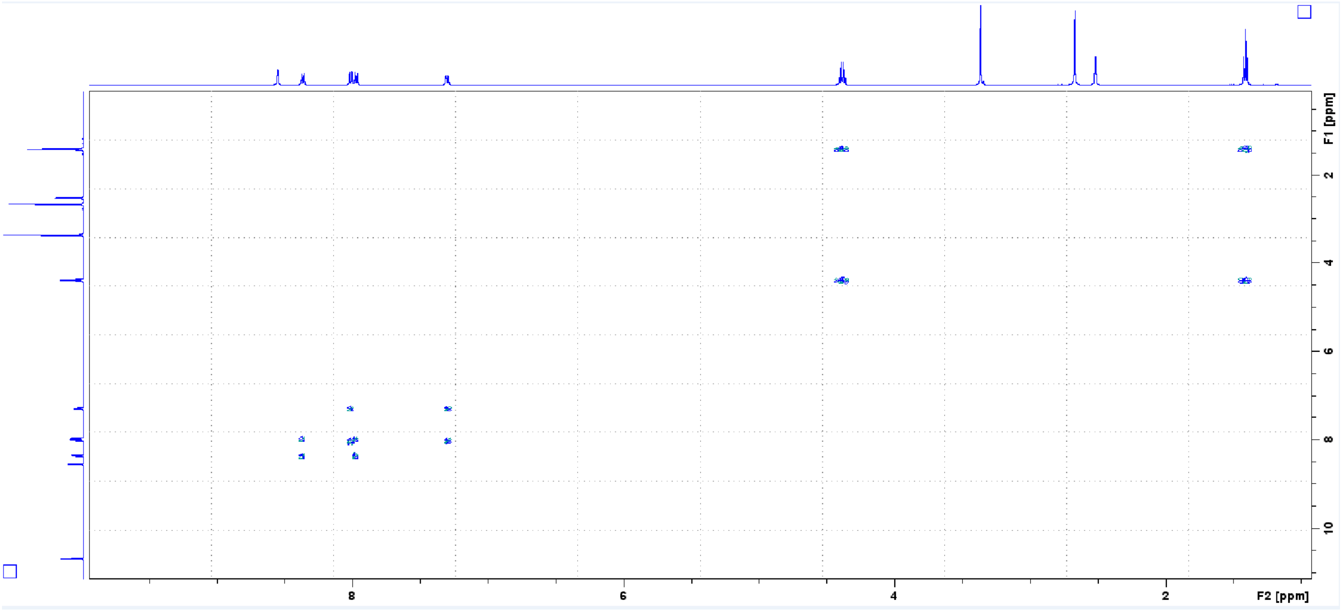

Figure S17 ^{1}H-^{1}H COSY spectrum of the compound **(4a)**

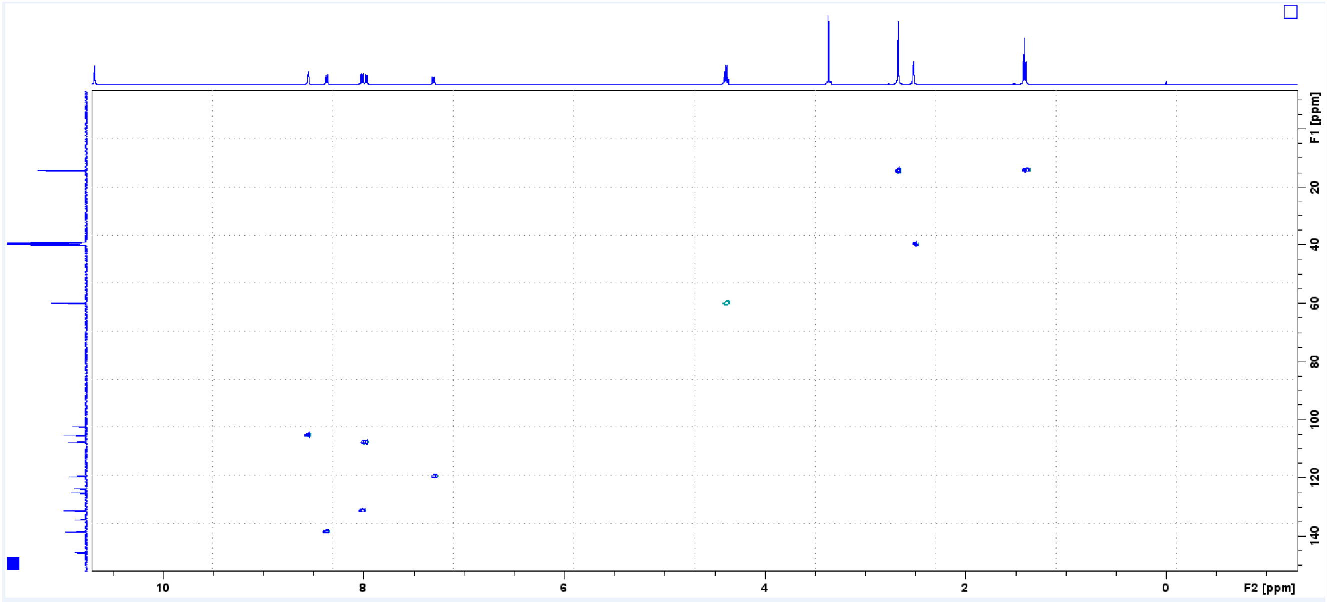

Figure S18 ^{1}H-^{13}C HSQC spectrum of the compound **(4a)**

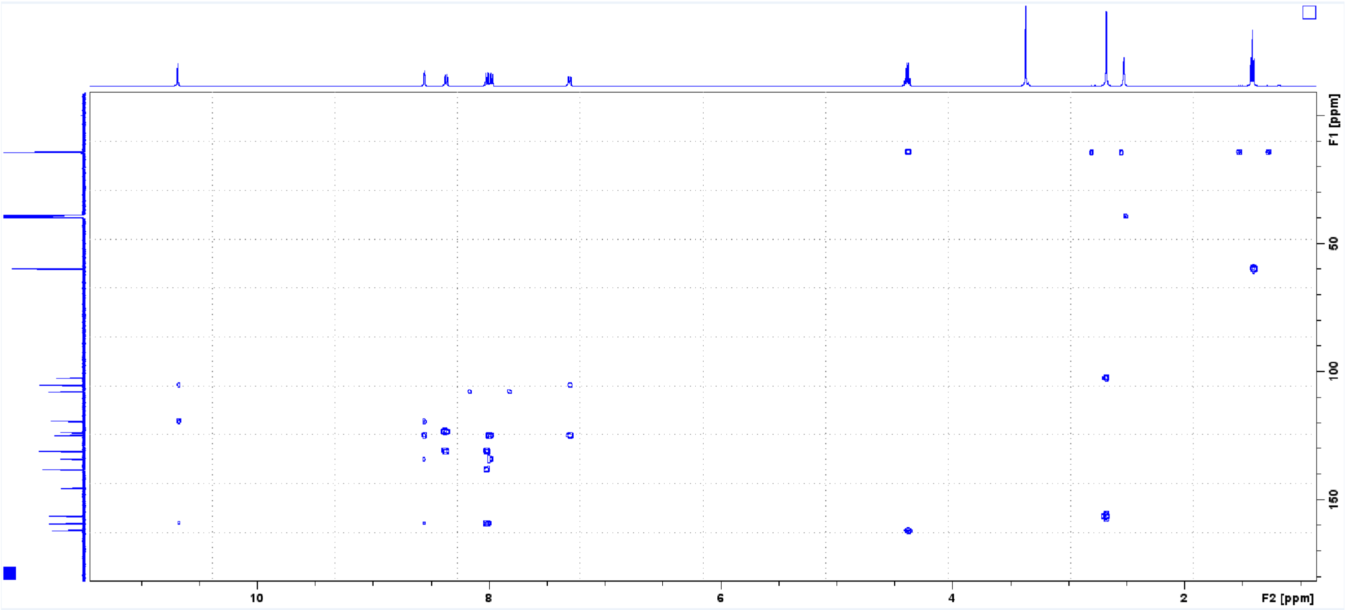

Figure S19 ^{1}H-^{13}C HMBC spectrum of the compound **(4a)**

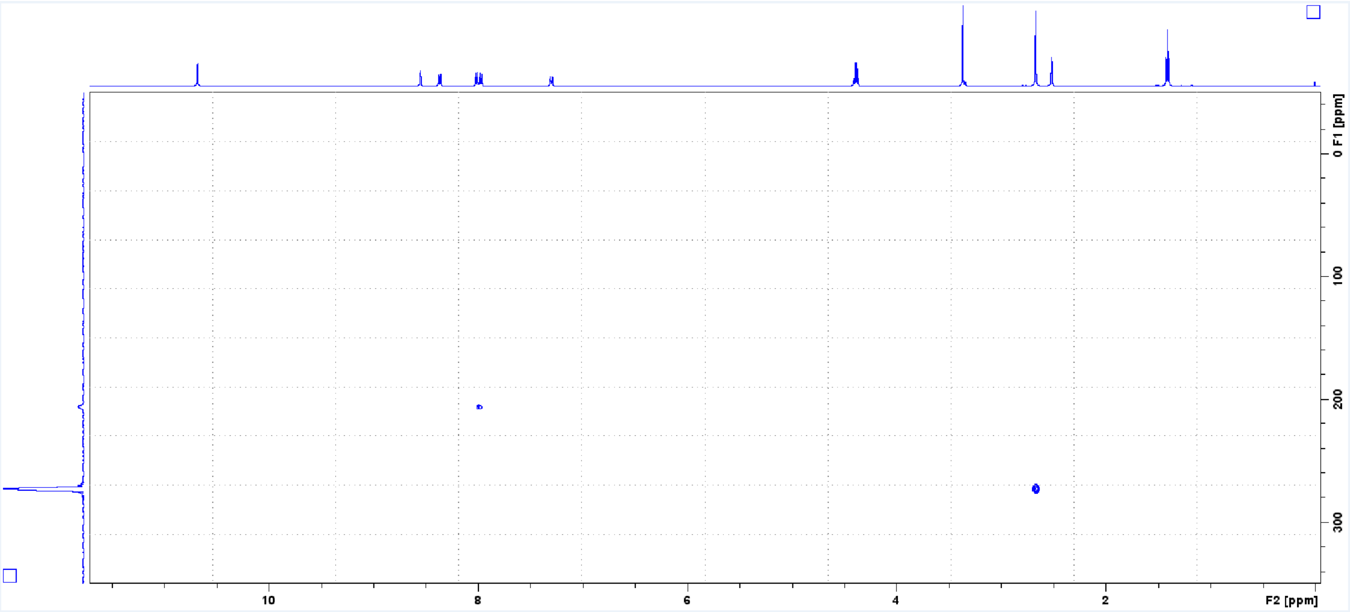

Figure S20 ^{1}H-^{15}N spectrum of the compound **(4a)**

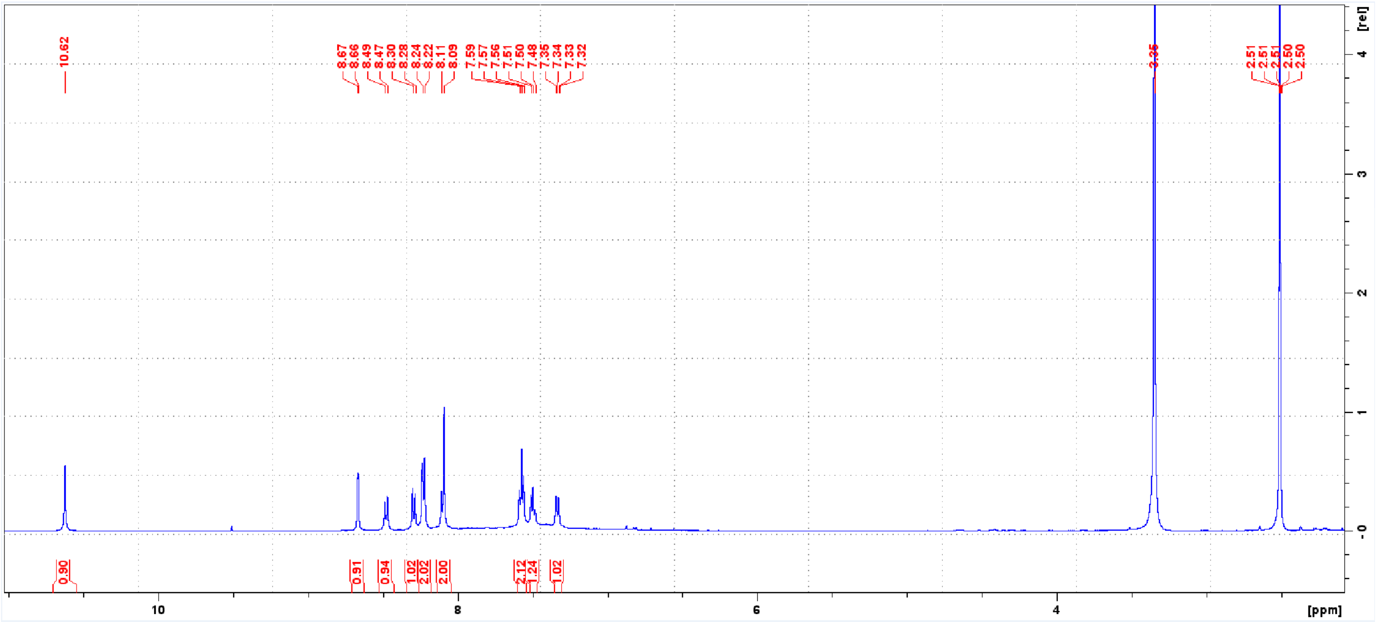

Figure S21 ^{1}H NMR spectrum of the compound **(4b)**

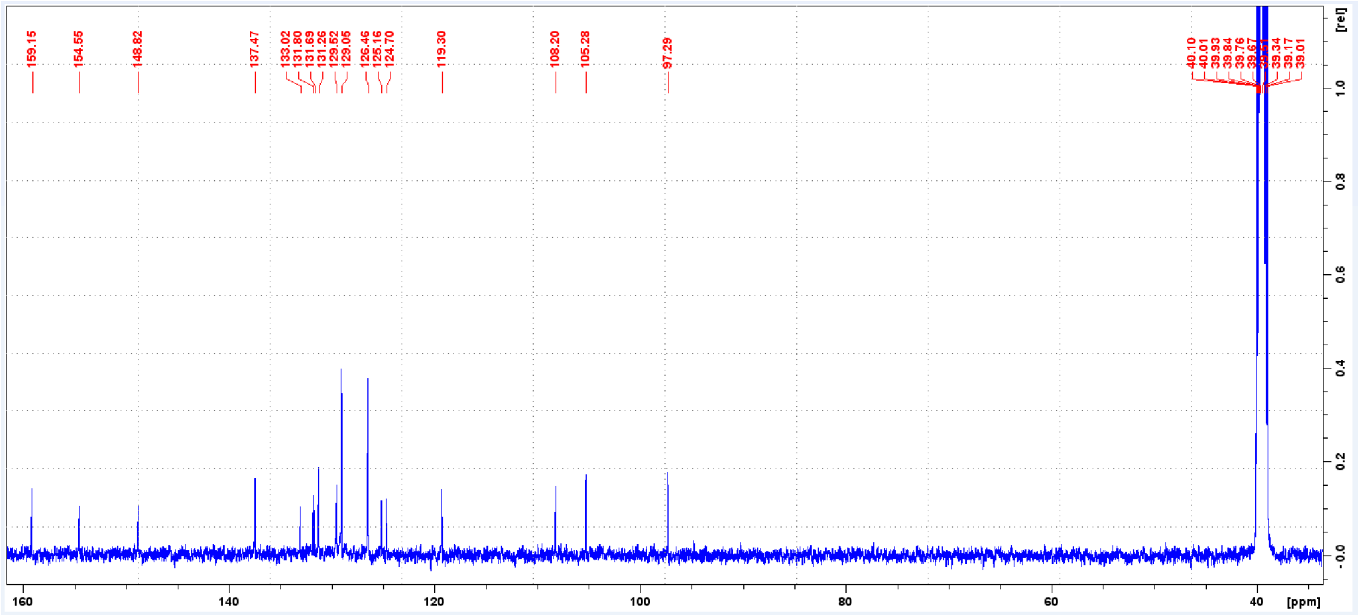

Figure S22 ^{13}C NMR spectrum of the compound **(4b)**

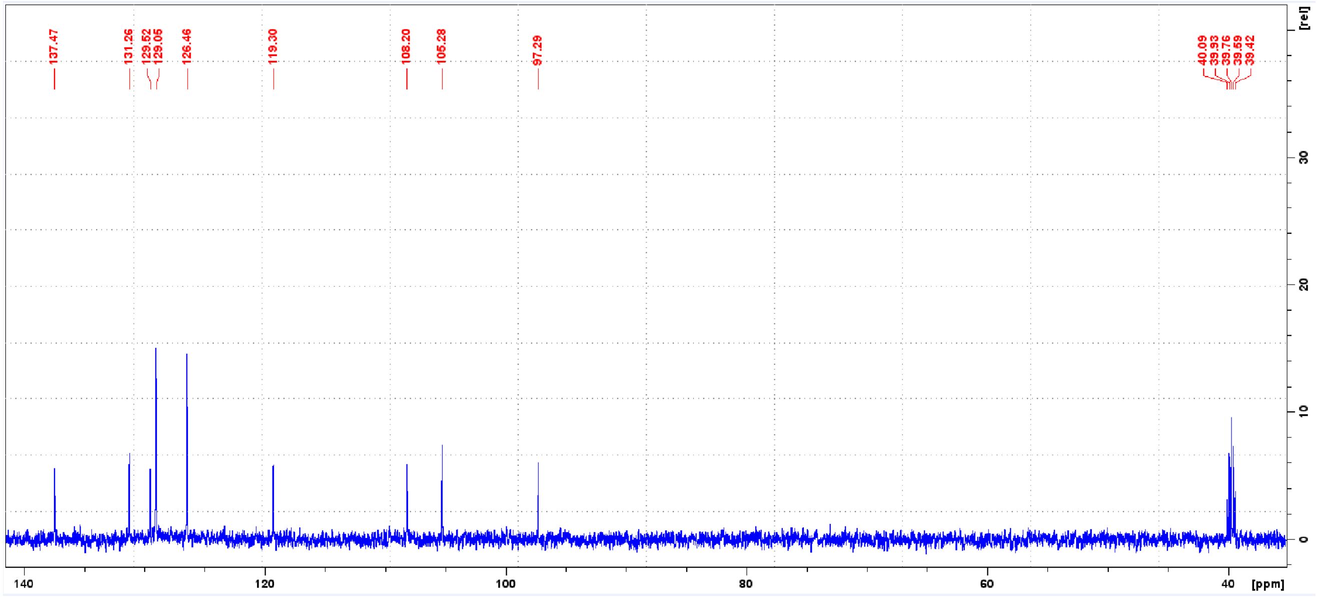

Figure S23 ^{13}C DEPT135 spectrum of the compound **(4b)**

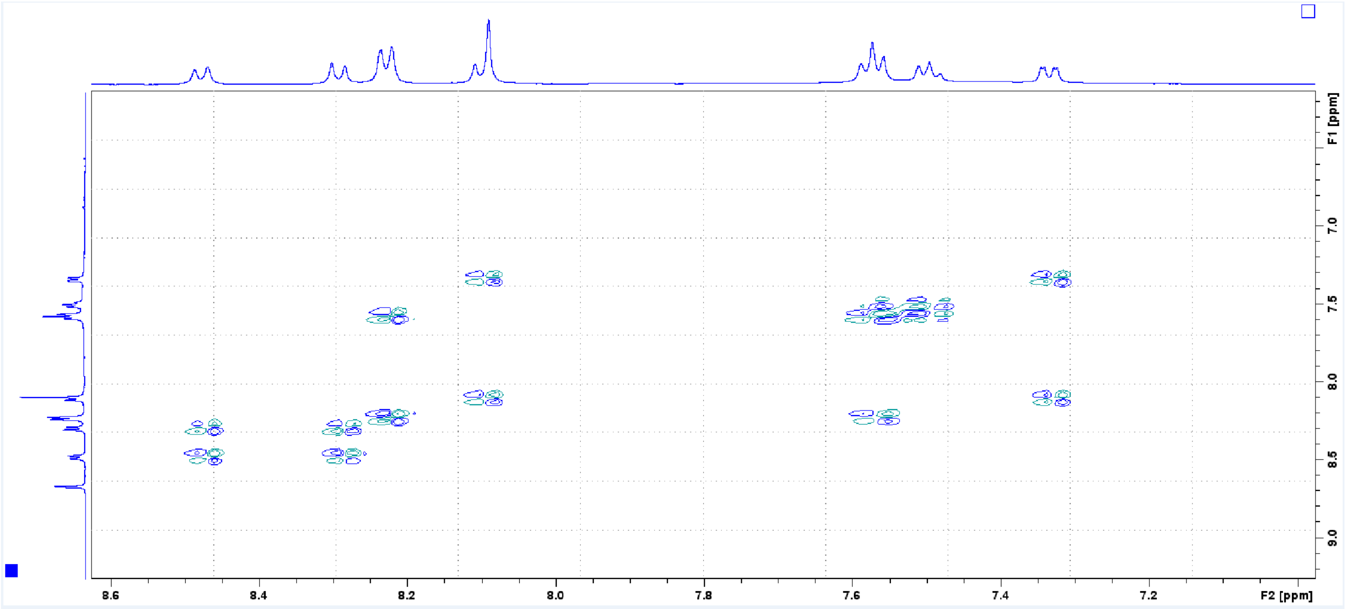

Figure S24 ^{1}H-^{1}H COSY spectrum of the compound **(4b)**

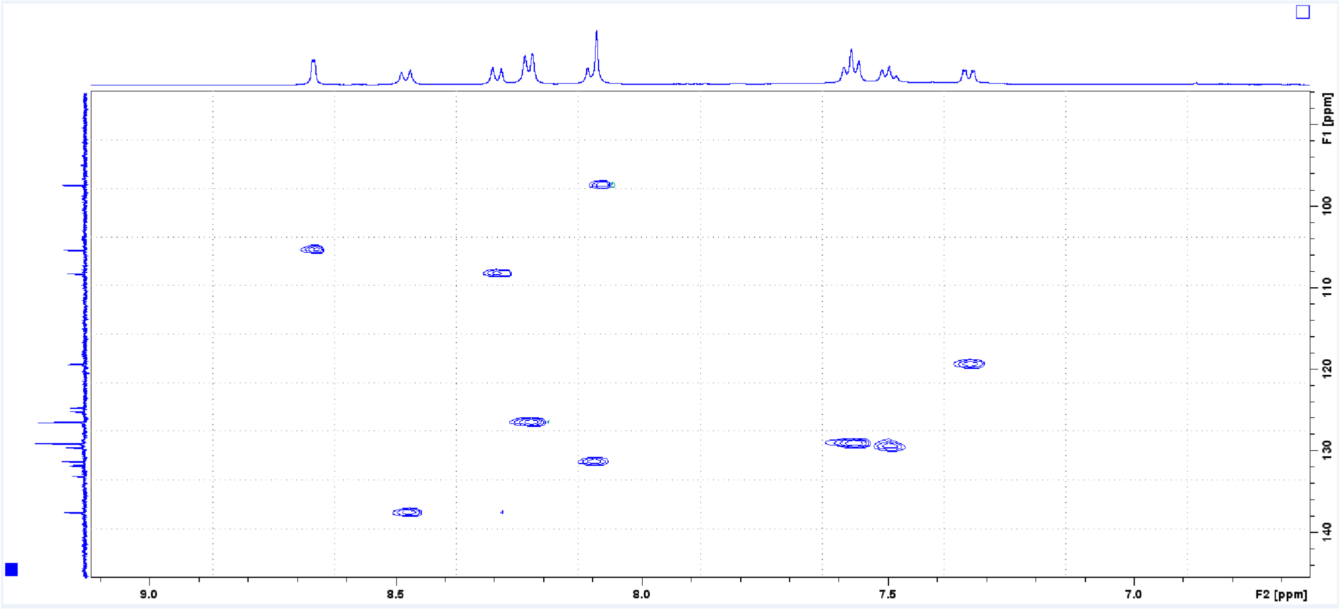

Figure S25 ^{1}H-^{13}C HSQC spectrum of the compound **(4b)**

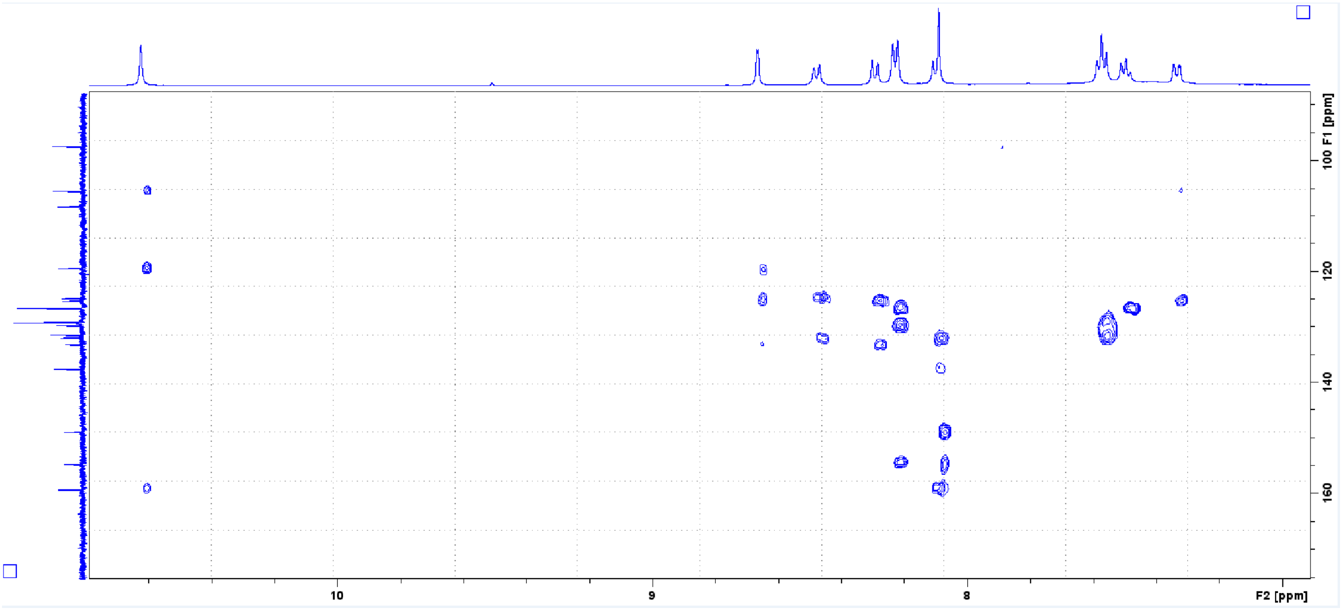

Figure S26 ^{1}H-^{13}C HMBC spectrum of the compound **(4b)**

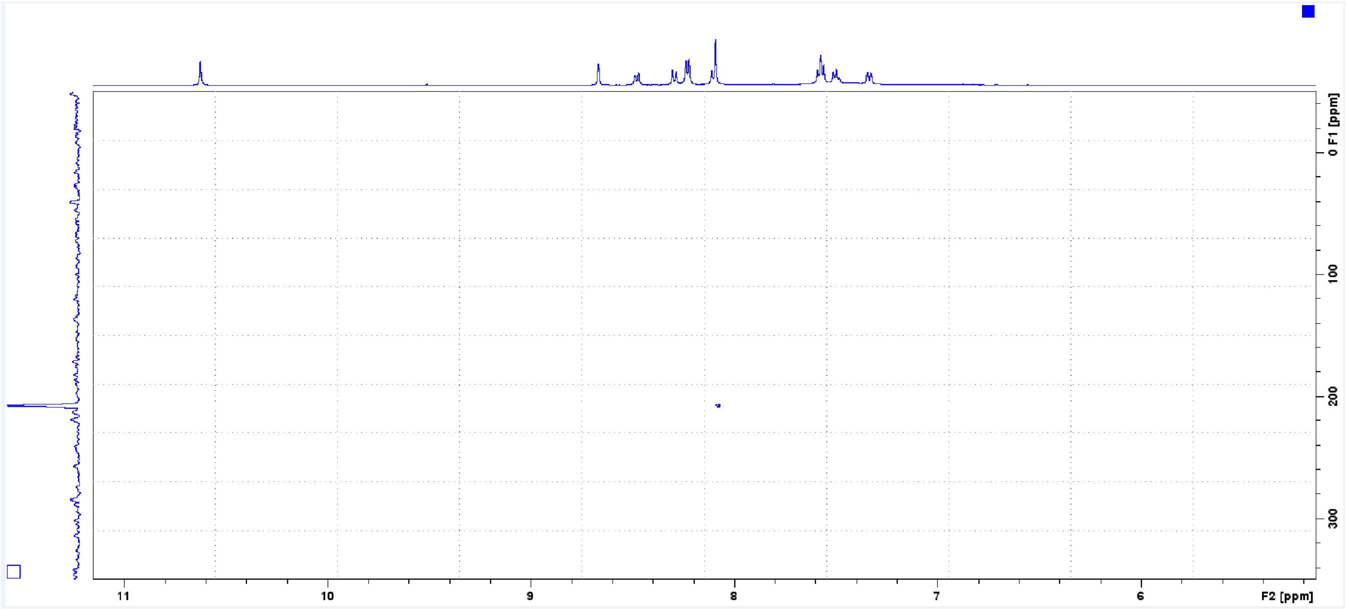

Figure S27 ^{1}H-^{15}N HMBC spectrum of the compound **(4b)**

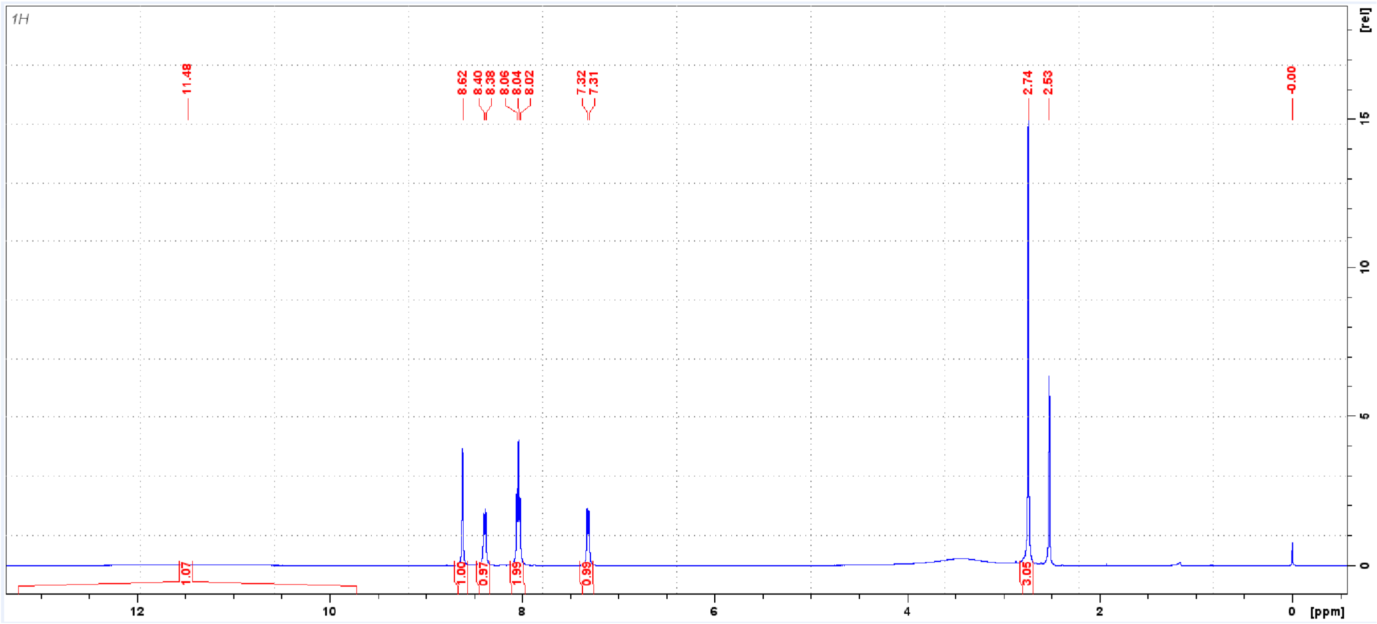

Figure S28 ^{1}H NMR spectrum of the compound **(5)**

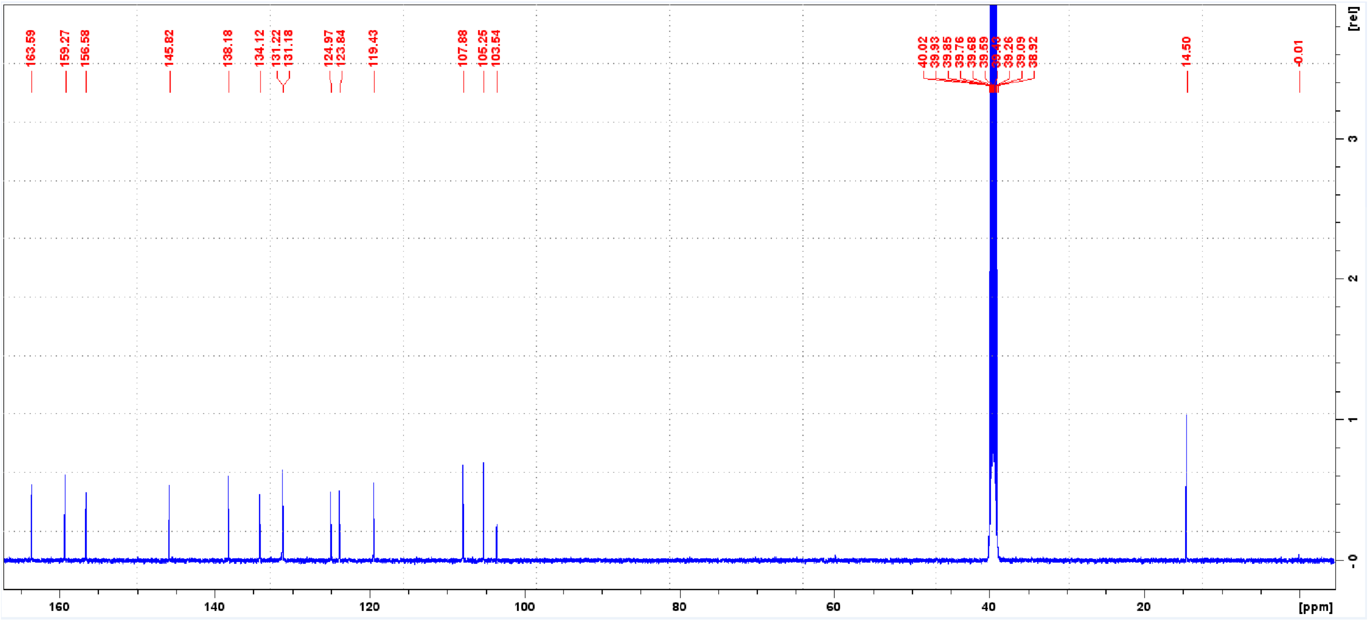

Figure S29 ^{13}C NMR spectrum of the compound **(5)**

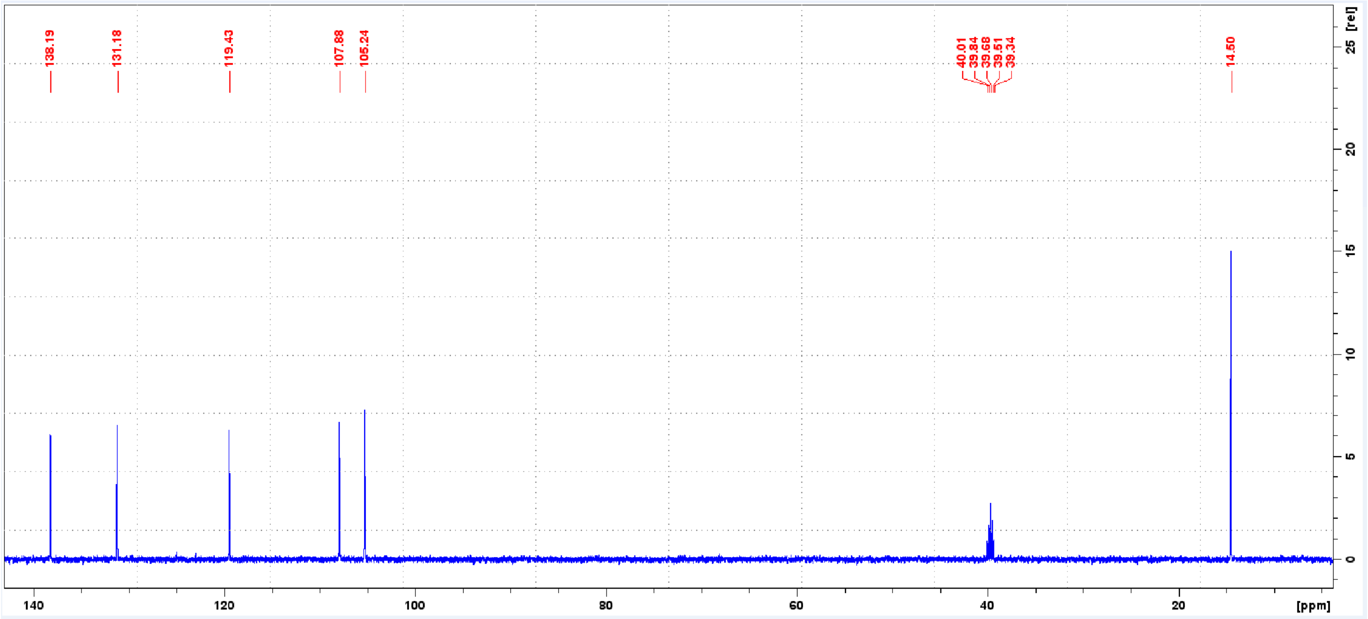

Figure S30 ^{13}C DEPT135 spectrum of the compound **(5)**

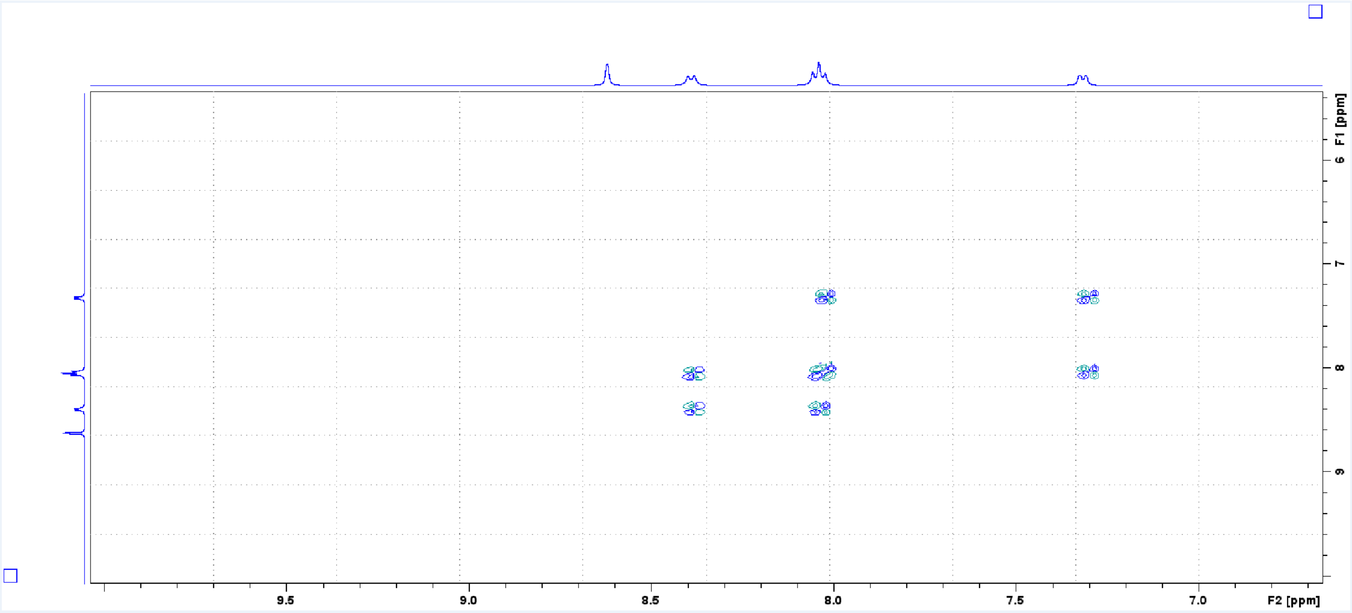

Figure S31 ^{1}H-^{1}H COSY spectrum of the compound **(5)**

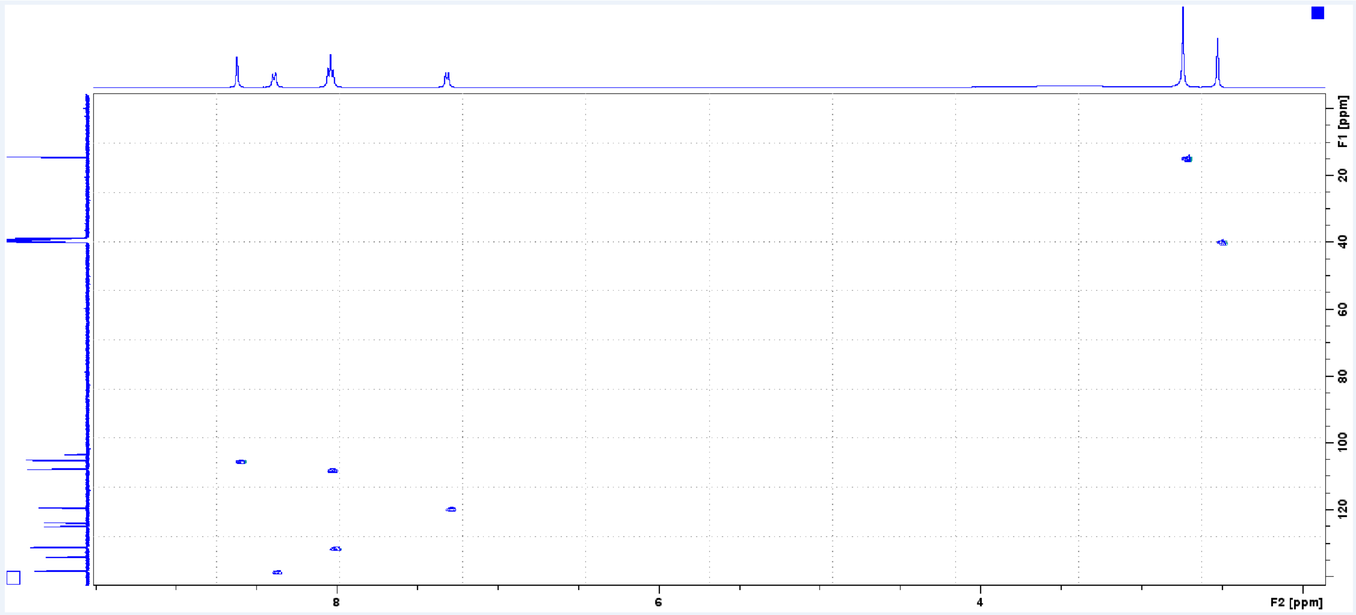

Figure S32 ^{1}H-^{13}C HSQC spectrum of the compound **(5)**

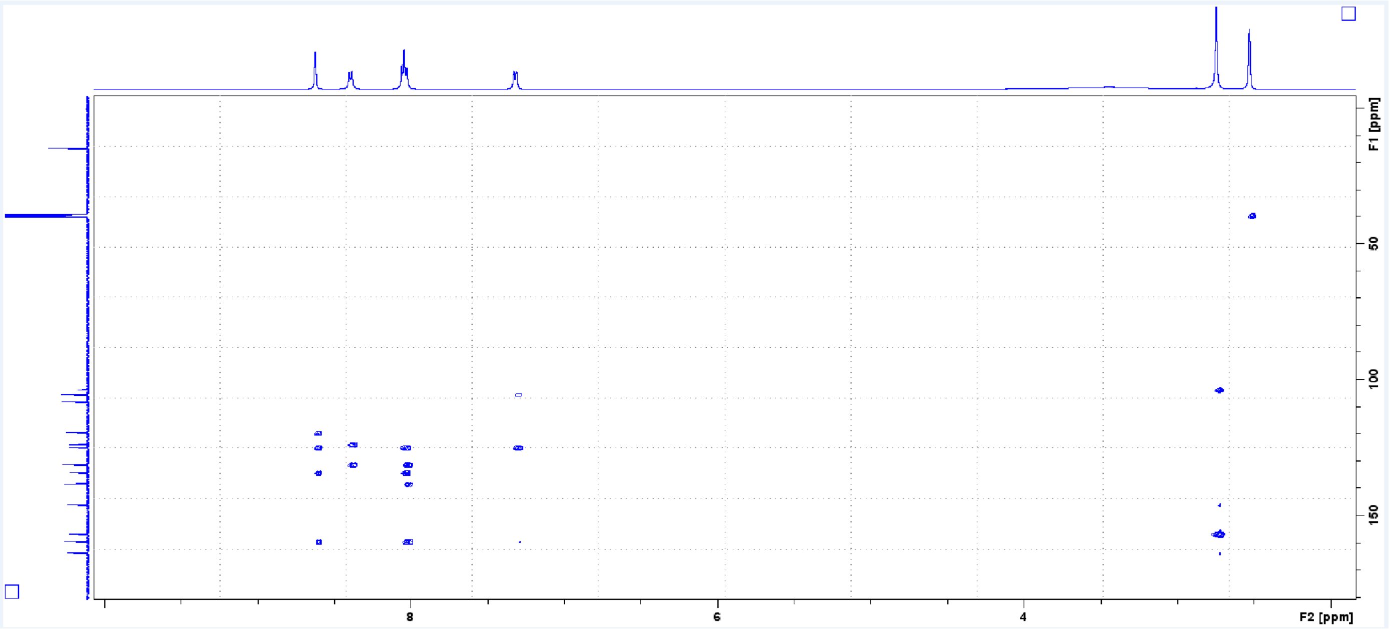

Figure S33 ^{1}H-^{13}C HMBC spectrum of the compound **(5)**

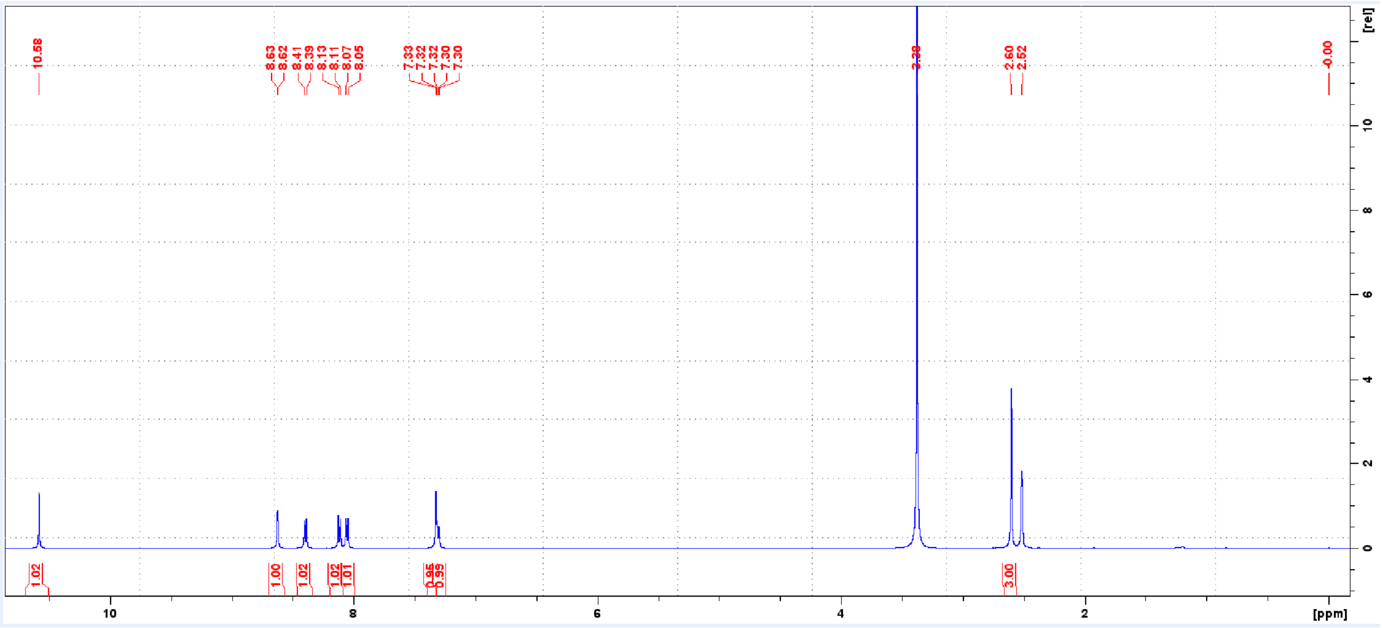

Figure S34 ^{1}H NMR spectrum of the compound **(6)**

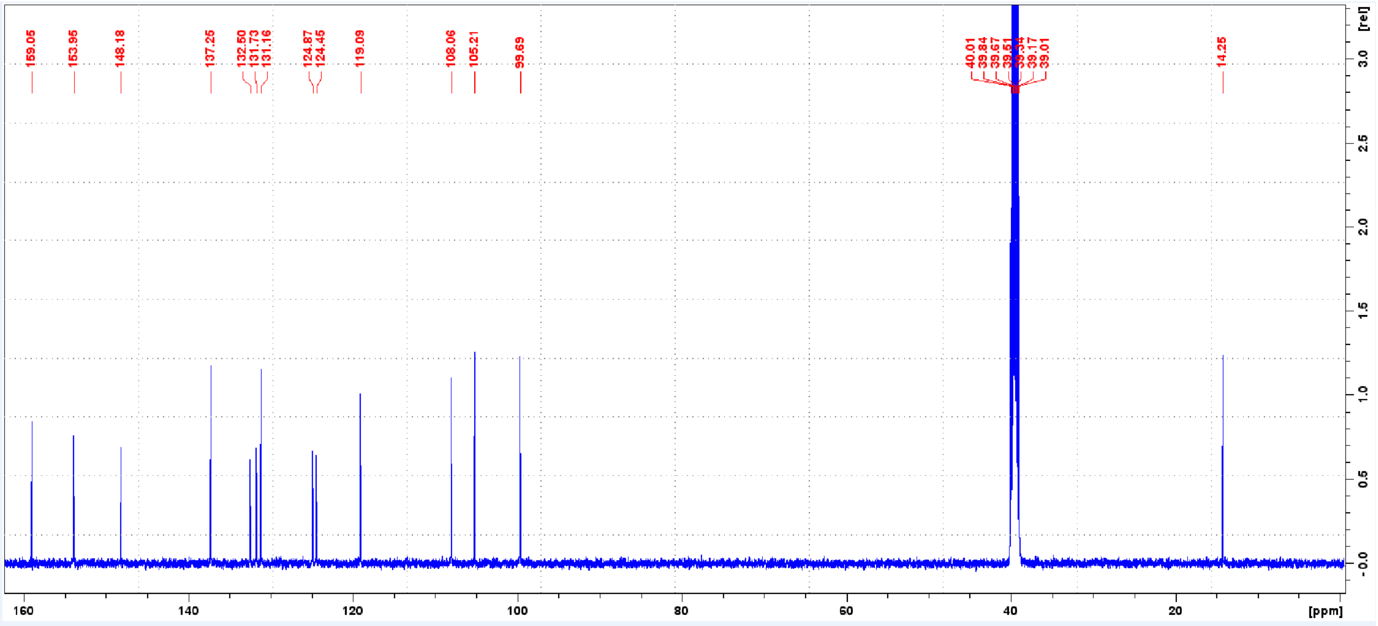

Figure S35 ^{13}C NMR spectrum of the compound **(6)**

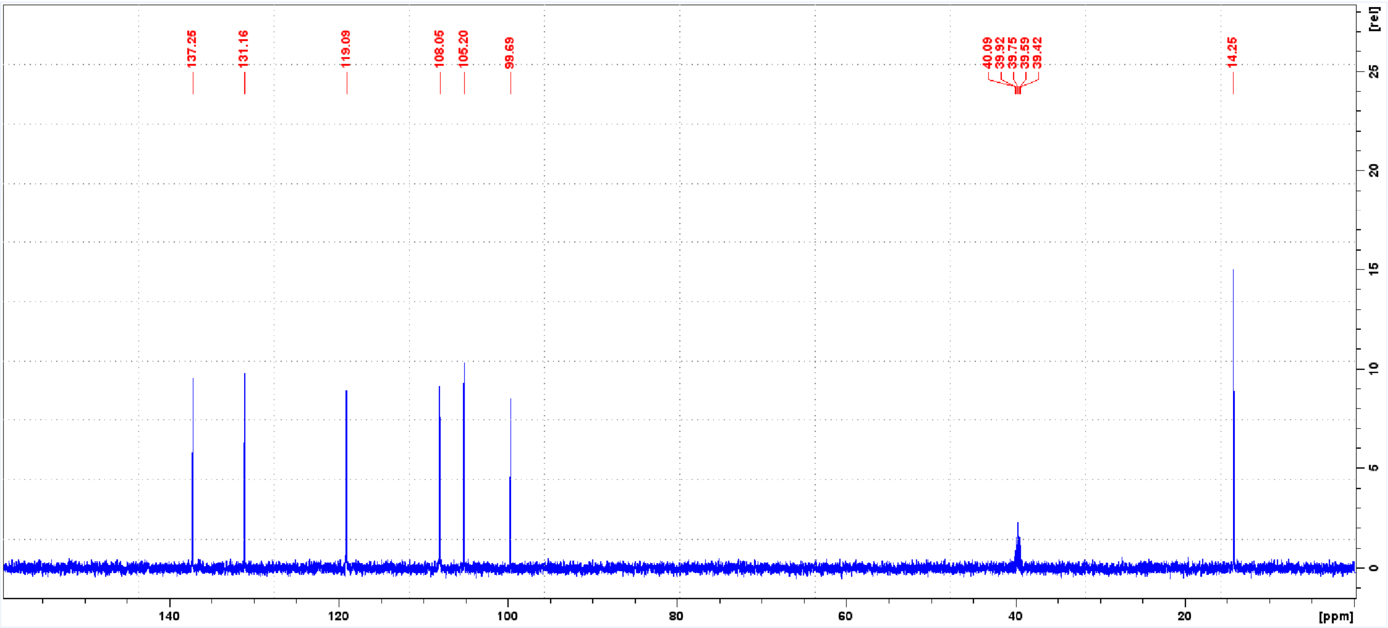

Figure S36 ^{13}C DEPT135 spectrum of the compound **(6)**

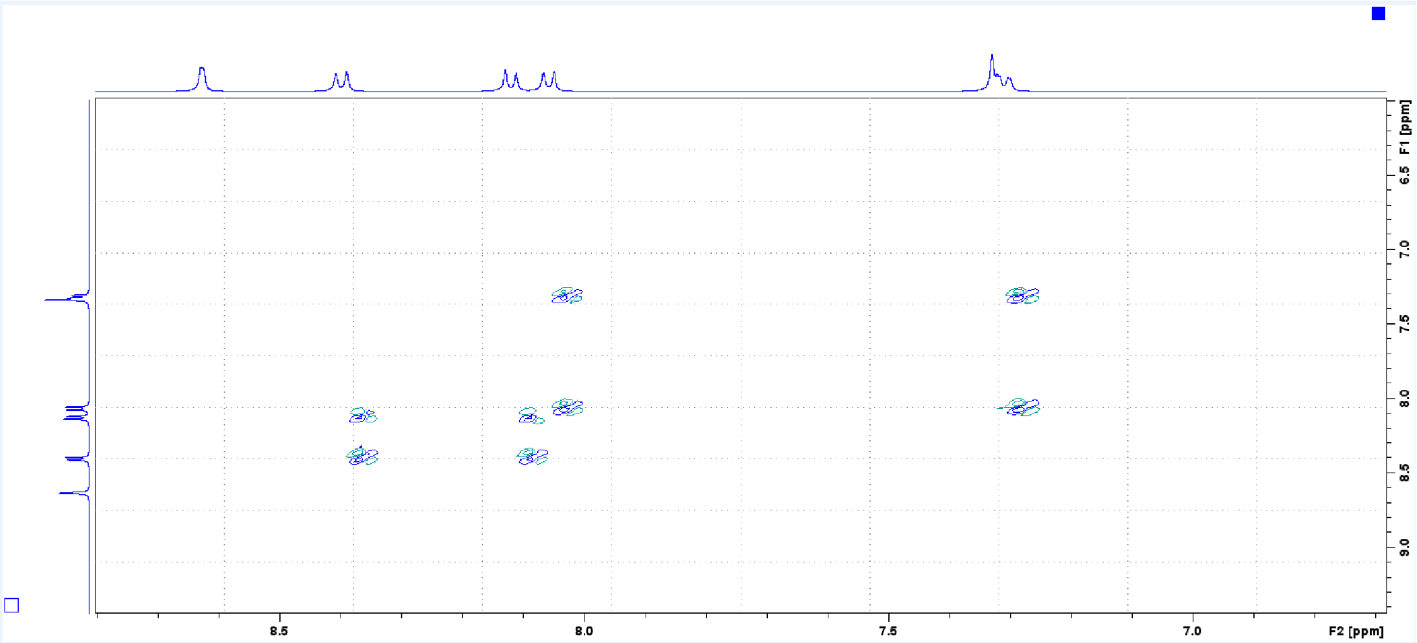

Figure S37 ^{1}H-^{1}H COSY spectrum of the compound **(6)**

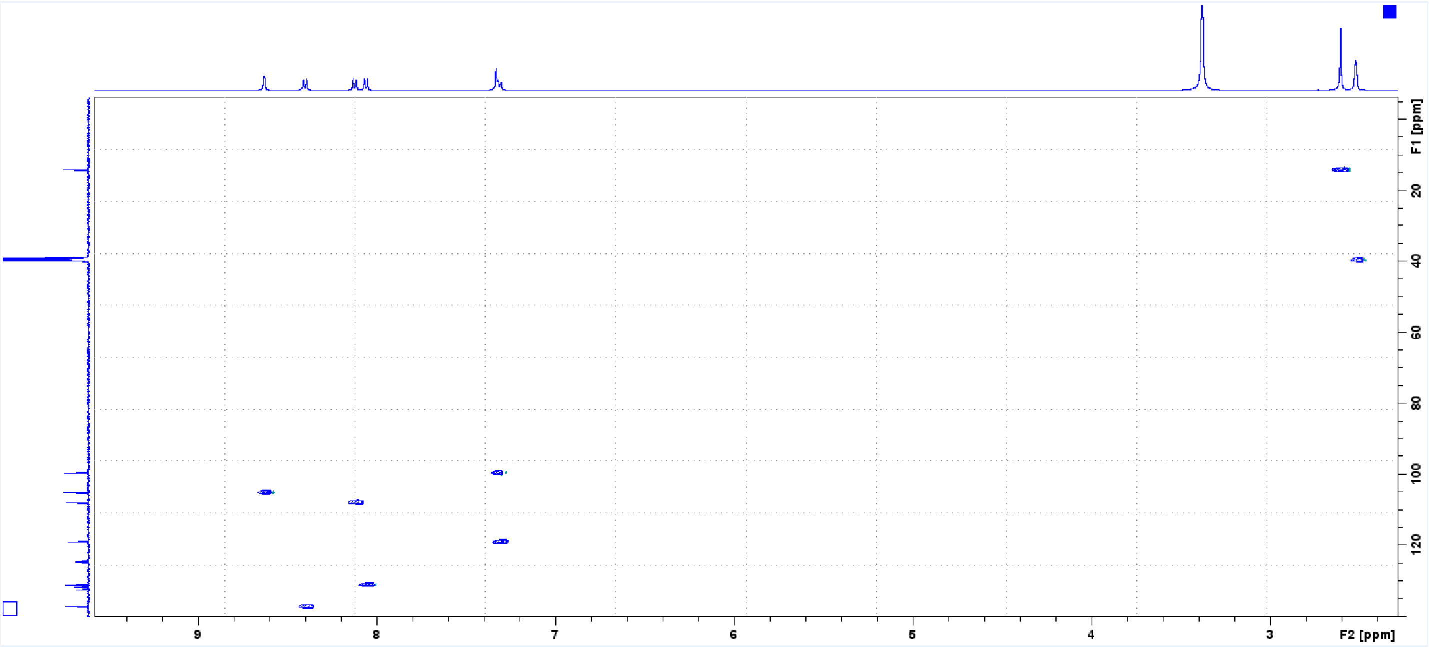

Figure S38 ^{1}H-^{13}C HSQC spectrum of the compound **(6)**

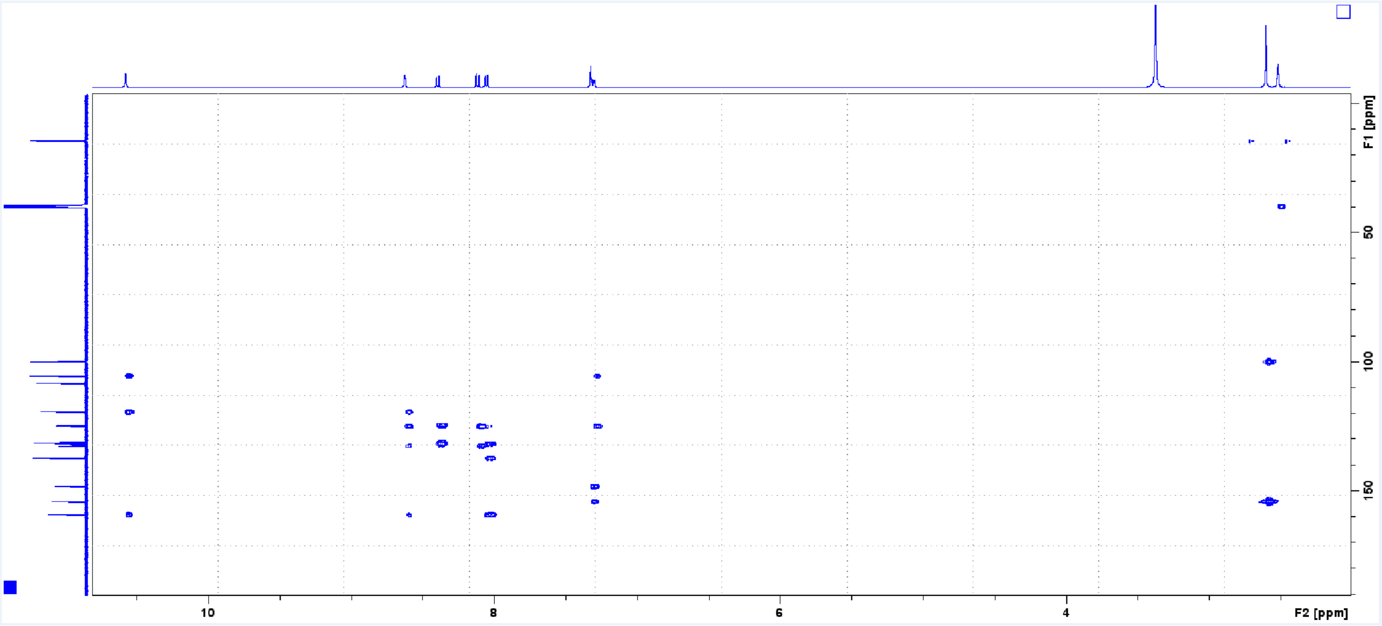

Figure S39 ^{1}H-^{13}C HMBC spectrum of the compound **(6)**

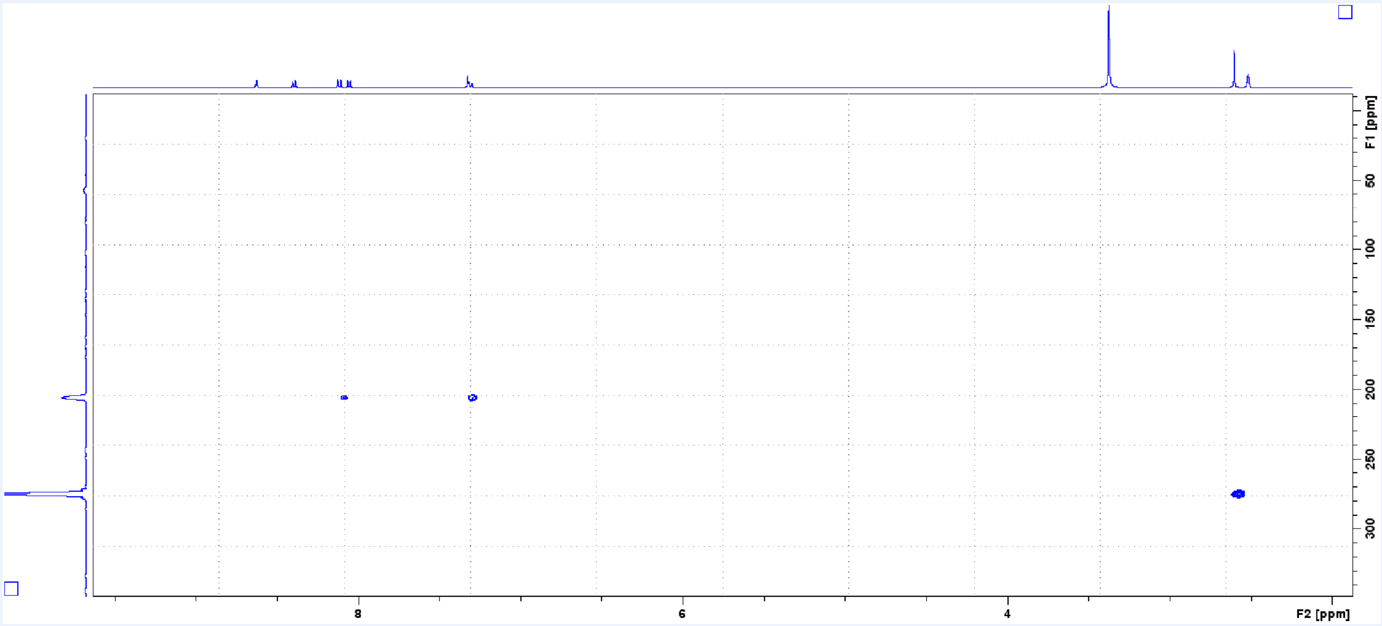

Figure S40 ^{1}H-^{15}N HMBC spectrum of the compound **(6)**

IR spectra for compounds (3a), (3b), (4a), (4b), (5), (6)

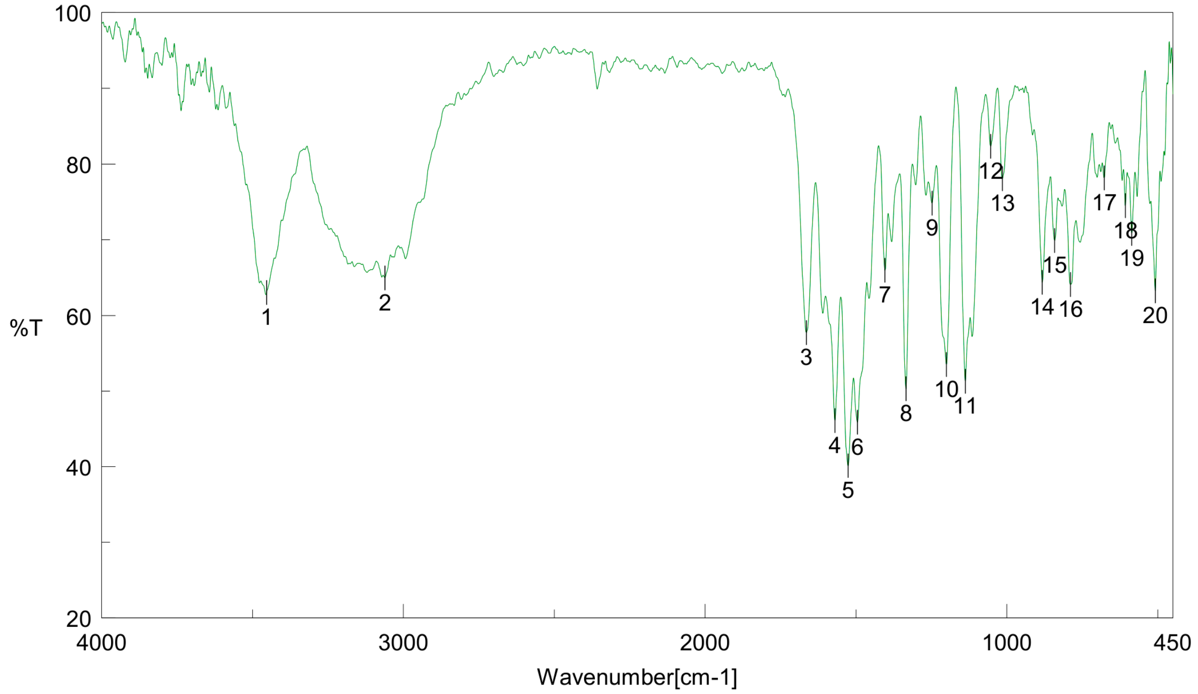

1: 3452.92, 63.0327	2: 3061.44, 64.9817	3: 1664.27, 57.7542
4: 1569.77, 46.1273	5: 1526.38, 40.128	6: 1495.53, 45.9228
7: 1403.92, 65.9894	8: 1334.5, 50.3563	9: 1247.72, 74.8606
10: 1200.47, 53.5419	11: 1137.8, 51.3271	12: 1053.91, 82.3594
13: 1015.34, 78.0672	14: 883.238, 64.3815	15: 841.776, 69.9065
16: 789.707, 64.0984	17: 677.856, 78.1691	18: 607.467, 74.5342
19: 586.254, 70.8395	20: 508.151, 63.2791	

Figure S41 IR spectrum of the compound **(3a)**

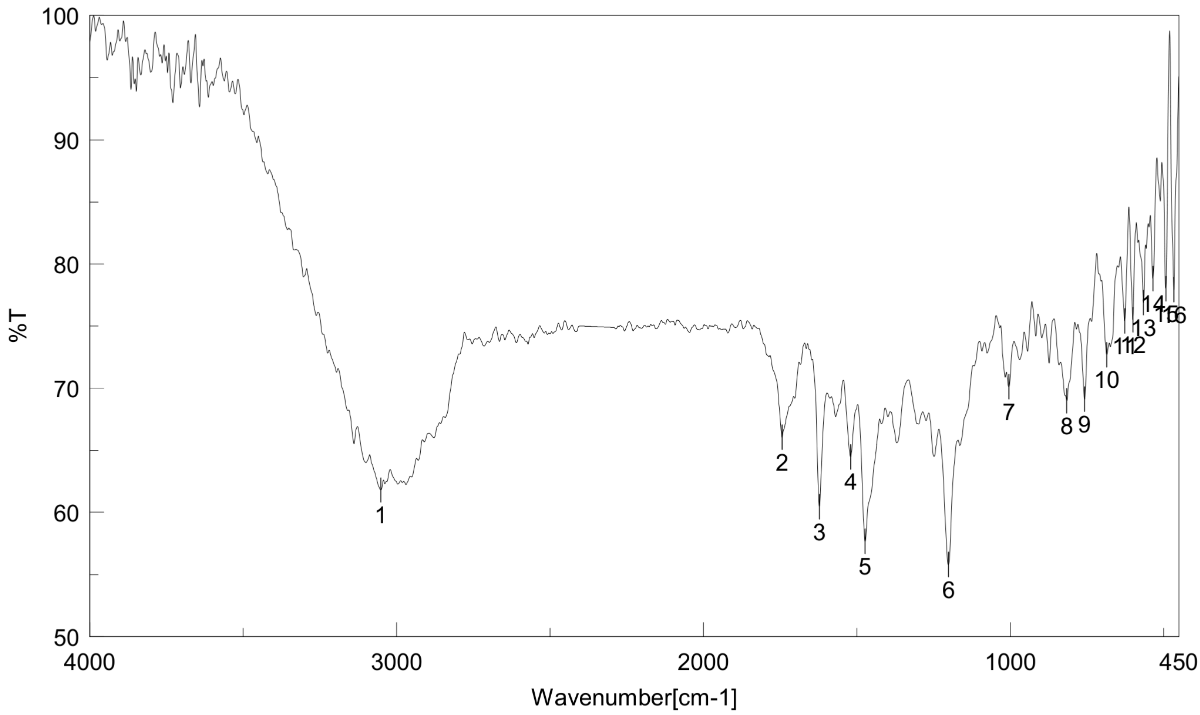

1: 3051.8, 61.7932	2: 1743.33, 66.0513	3: 1621.84, 60.4442
4: 1519.63, 64.4731	5: 1473.35, 57.6641	6: 1201.43, 55.8005
7: 1004.73, 70.1336	8: 815.742, 68.9956	9: 757.888, 69.125
10: 685.57, 72.6947	11: 626.752, 75.4073	12: 599.753, 75.5011
13: 566.005, 76.8866	14: 534.185, 78.806	15: 492.723, 77.9849
16: 466.689, 77.9178		

Figure S42 IR spectrum of the compound **(3b)**

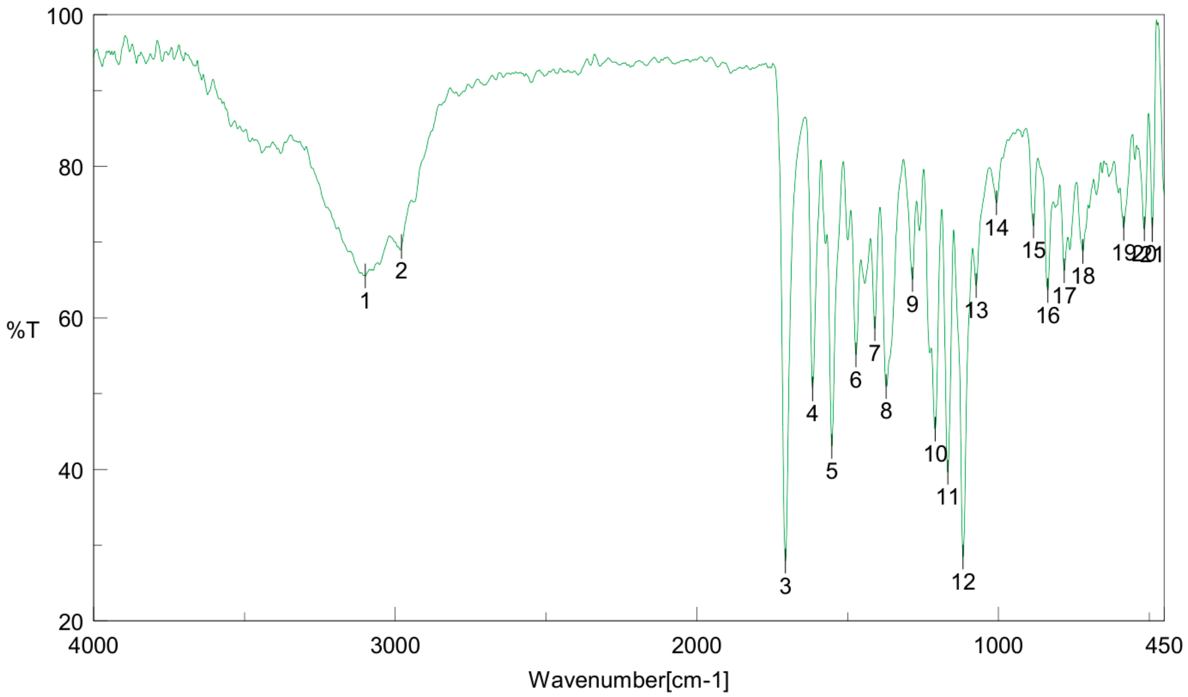

1: 3099.05, 65.5491	2: 2979.48, 69.4306	3: 1706.69, 27.9084
4: 1616.06, 50.647	5: 1552.42, 43.0649	6: 1472.38, 55.1283
7: 1409.71, 58.577	8: 1372.1, 50.9561	9: 1285.32, 65.0831
10: 1210.11, 45.3516	11: 1167.69, 39.6266	12: 1117.55, 28.4712
13: 1074.16, 64.2982	14: 1006.66, 75.2042	15: 884.202, 72.1646
16: 836.955, 63.6133	17: 781.993, 66.2285	18: 720.282, 68.7744
19: 584.326, 71.8321	20: 516.829, 71.7776	21: 489.831, 71.636

Figure S43 IR spectrum of the compound **(4a)**

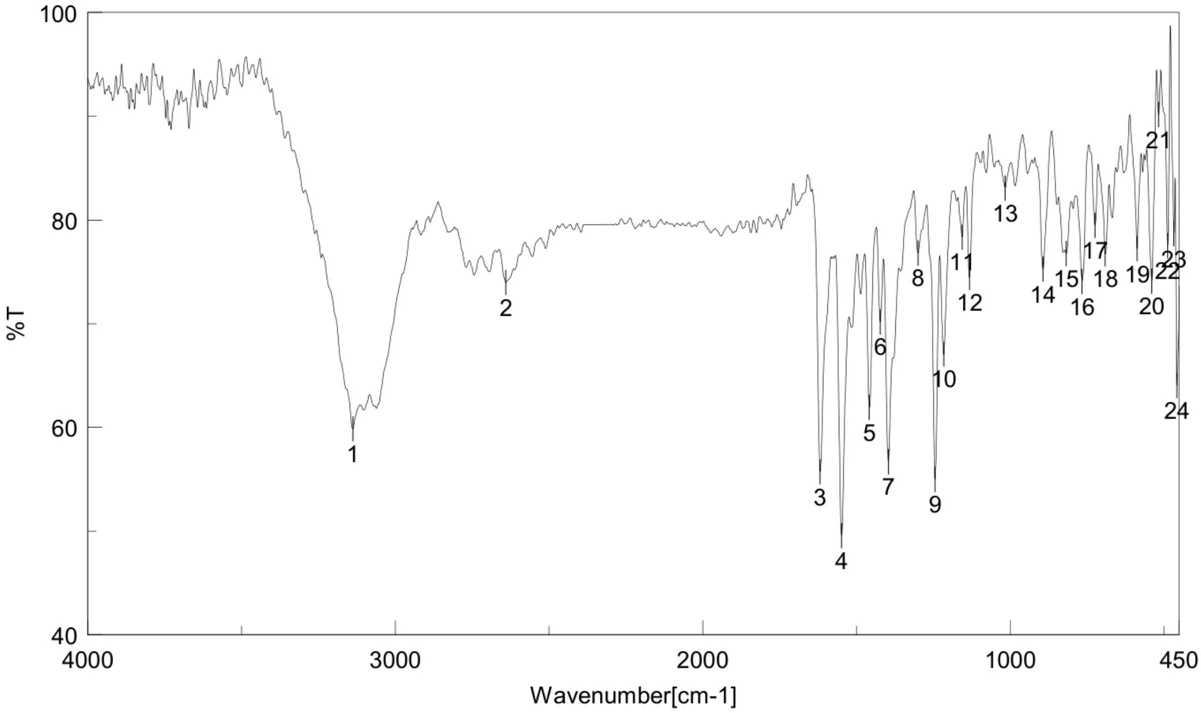

1: 3137.62, 59.8373	2: 2640.07, 74.0031	3: 1617.98, 55.7171
4: 1548.56, 49.5468	5: 1457.92, 61.9153	6: 1422.24, 70.1954
7: 1396.21, 56.6671	8: 1299.79, 76.8403	9: 1243.86, 54.9799
10: 1215.9, 67.0502	11: 1156.12, 78.358	12: 1132.01, 74.4855
13: 1016.3, 83.096	14: 892.88, 75.2742	15: 817.67, 76.79
16: 766.566, 74.0702	17: 724.139, 79.5019	18: 690.391, 76.7392
19: 587.218, 77.2479	20: 539.007, 74.1045	21: 516.829, 90.1774
22: 487.902, 77.5396	23: 467.653, 78.6718	24: 457.047, 64.0175

Figure S44 IR spectrum of the compound **(4b)**

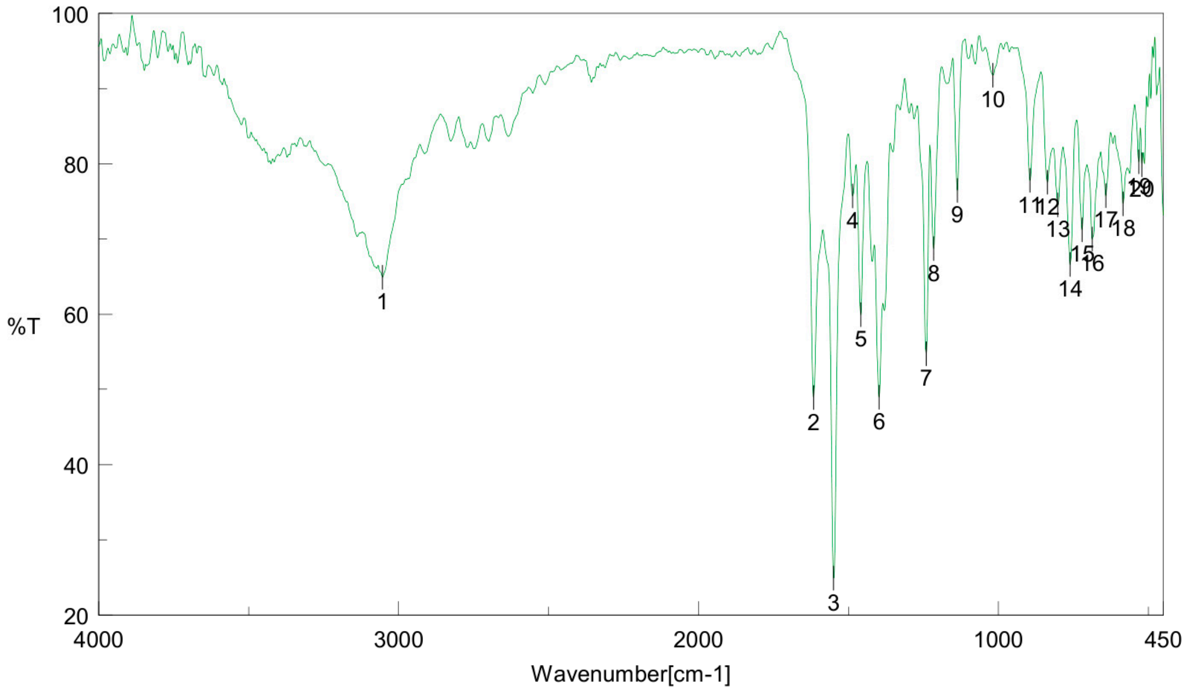

1: 3053.73, 64.9343	2: 1616.06, 48.9089	3: 1549.52, 24.9279
4: 1485.88, 75.7658	5: 1458.89, 59.9782	6: 1398.14, 48.9293
7: 1240.97, 54.7145	8: 1215.9, 68.7329	9: 1136.83, 76.4847
10: 1018.23, 91.8025	11: 894.809, 77.8091	12: 836.955, 77.5566
13: 802.242, 74.5729	14: 760.78, 66.6582	15: 721.247, 71.2407
16: 687.498, 70.0179	17: 642.18, 75.7943	18: 584.326, 74.6833
19: 532.257, 80.2931	20: 521.65, 79.8914	

Figure S45 IR spectrum of the compound **(5)**

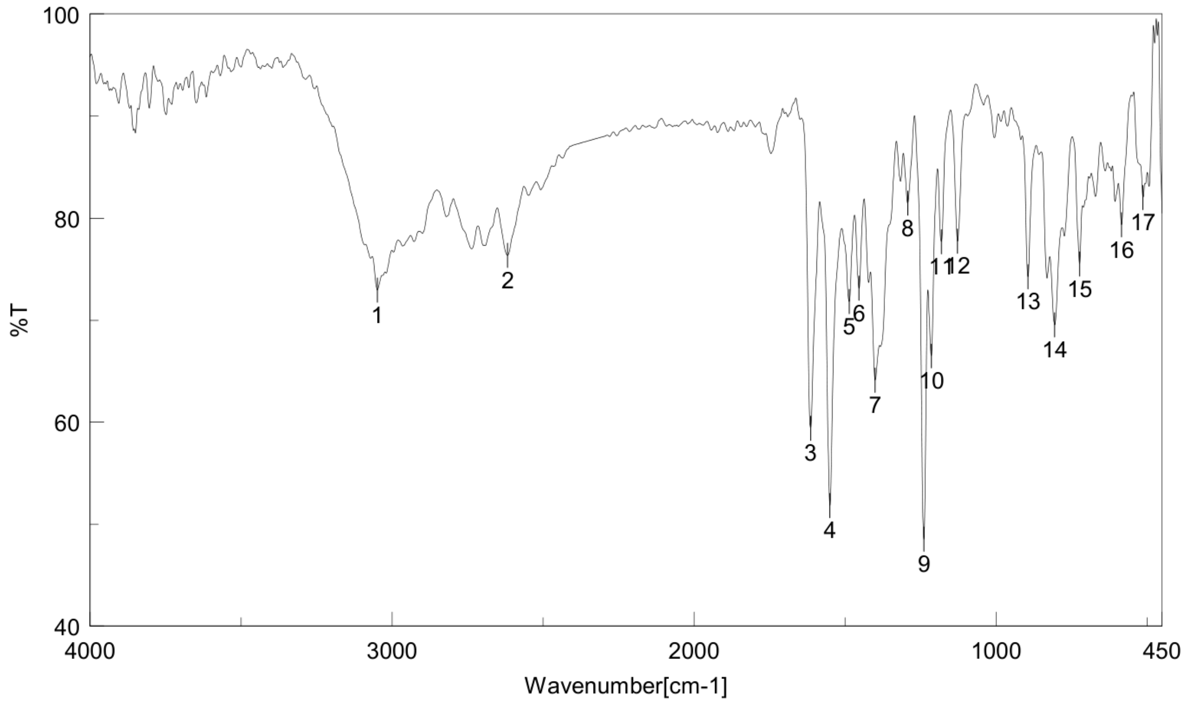

1: 3048.91, 72.98	2: 2617.89, 76.3705	3: 1614.13, 59.3809
4: 1550.49, 51.8131	5: 1485.88, 71.8578	6: 1454.06, 73.1536
7: 1400.07, 64.087	8: 1292.07, 81.4865	9: 1239.04, 48.5185
10: 1213.97, 66.486	11: 1181.19, 77.7134	12: 1127.19, 77.7642
13: 893.844, 74.2766	14: 806.099, 69.551	15: 723.175, 75.5382
16: 584.326, 79.3648	17: 512.972, 82.0379	

Figure S46 IR spectrum of the compound **(6)**

UV-Vis spectra for compounds (3a), (3b), (4a), (4b), (5), (6)

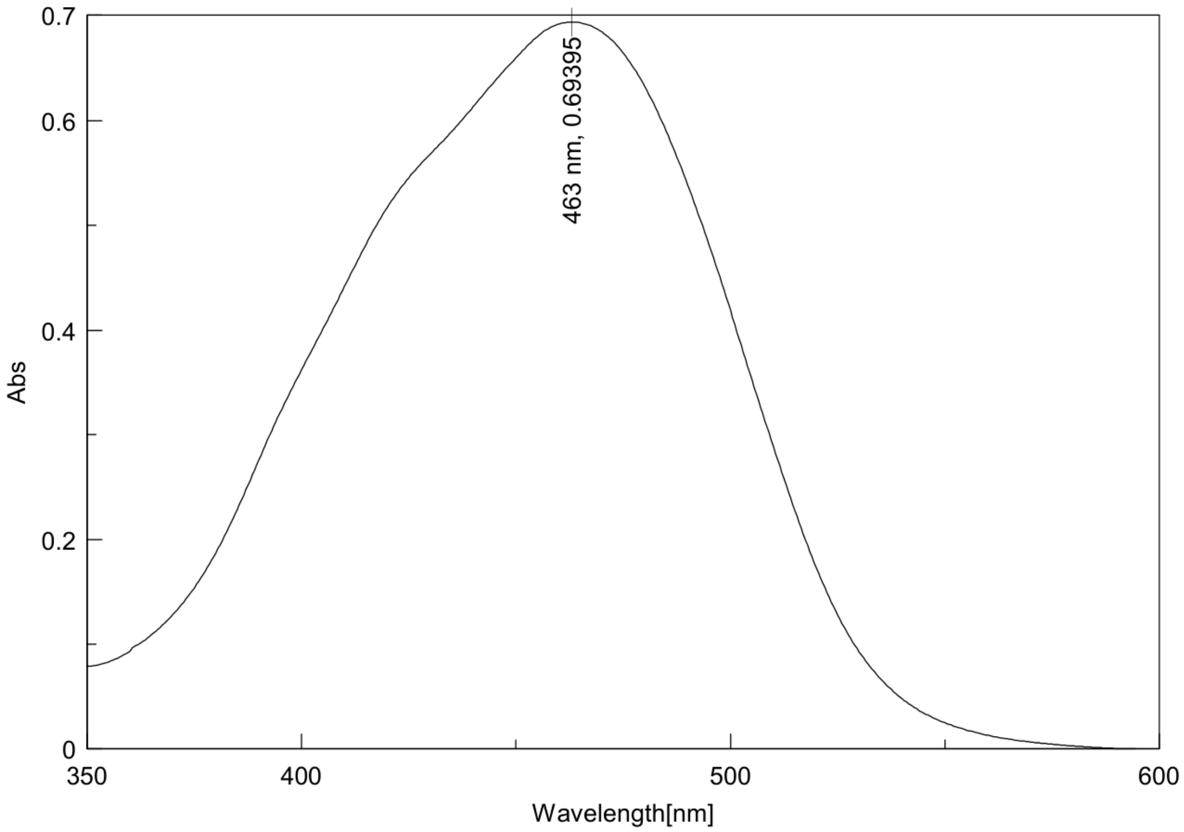

Figure S47 UV-Vis spectrum of the compound **(3a)**

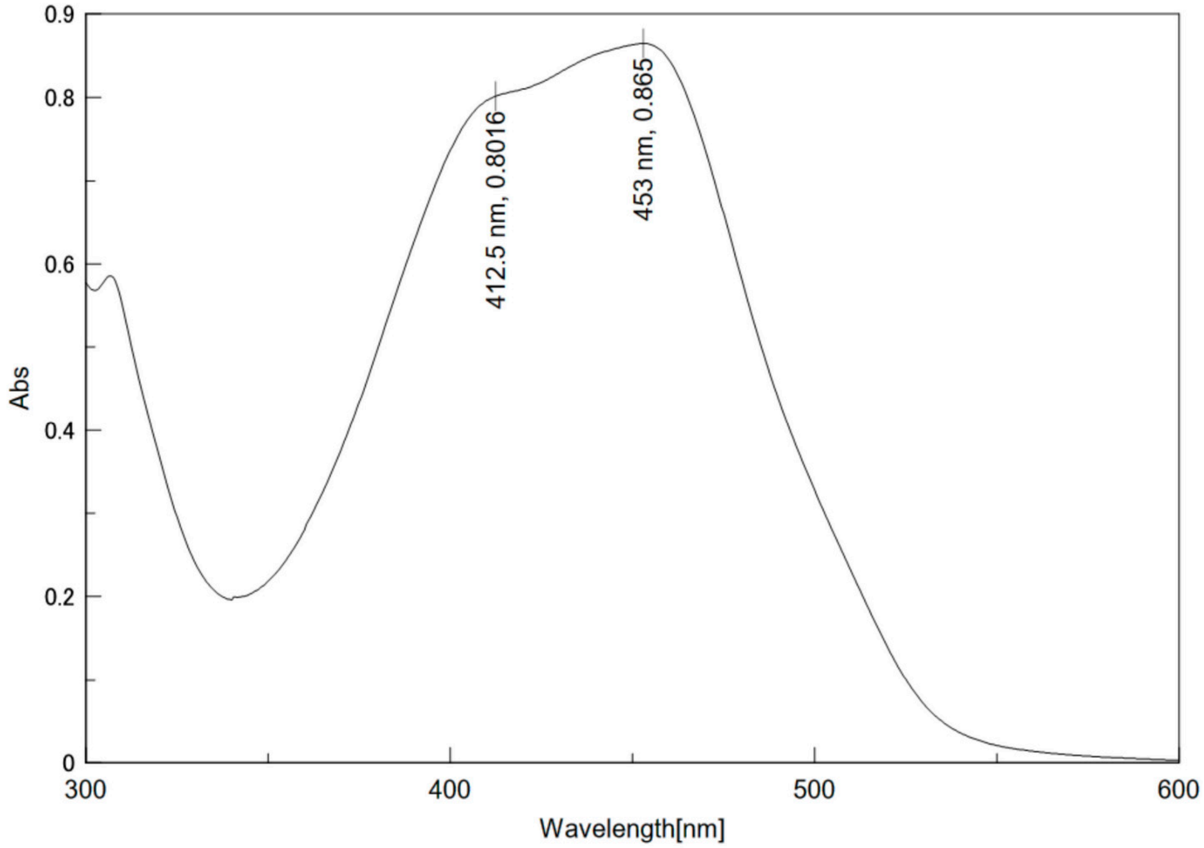

Figure S48 UV-Vis spectrum of the compound **(3b)**

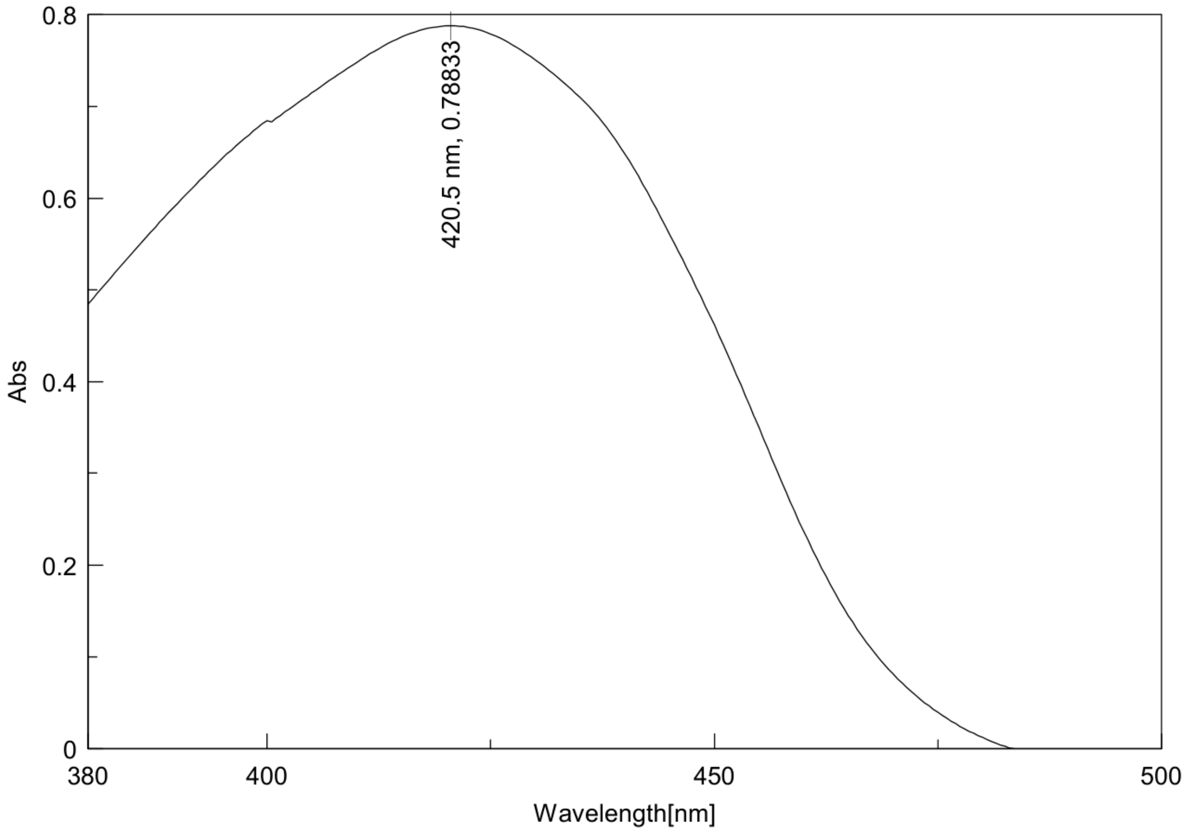

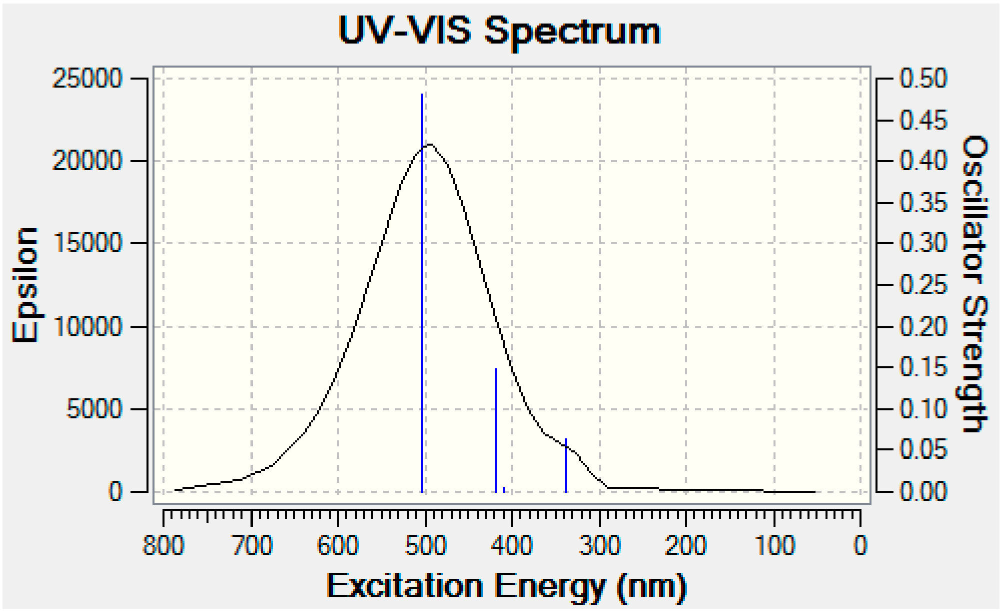

Figure S49 Measured (top) and calculated (bottom) UV-Vis spectra of the compound **(4a)**

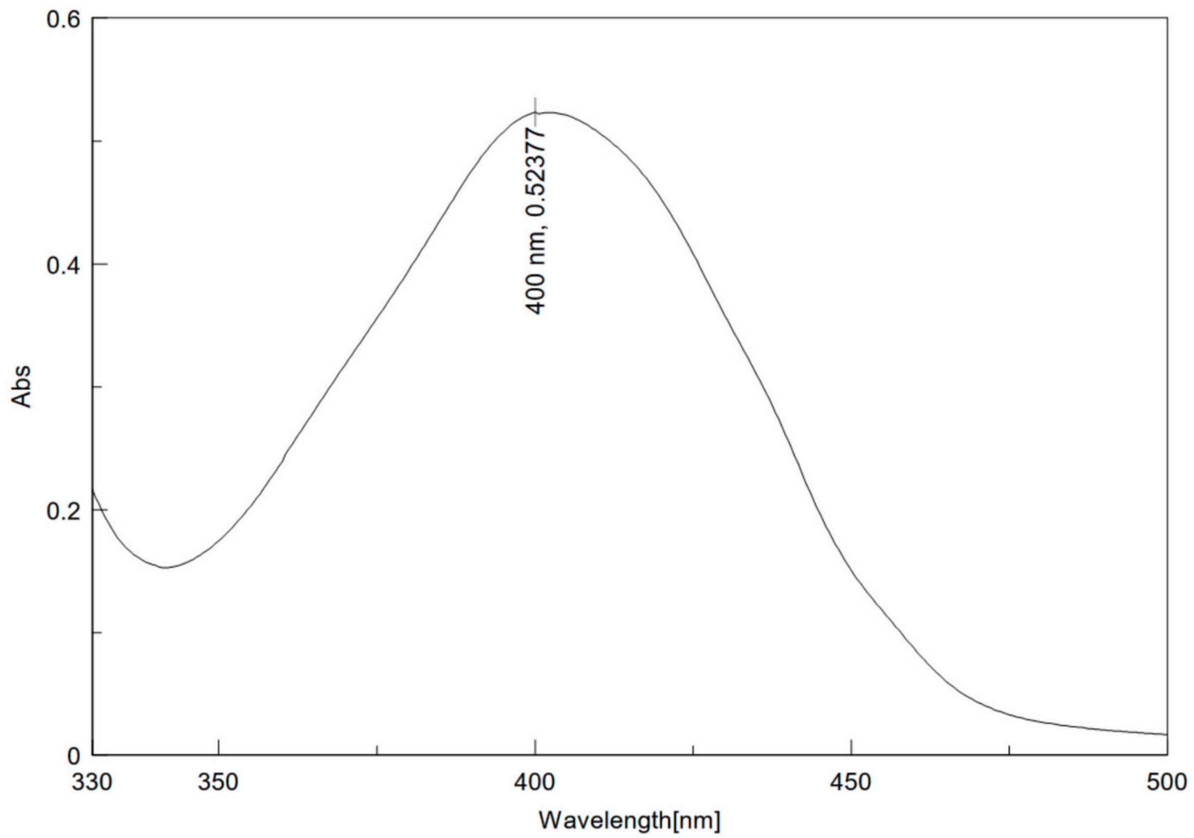

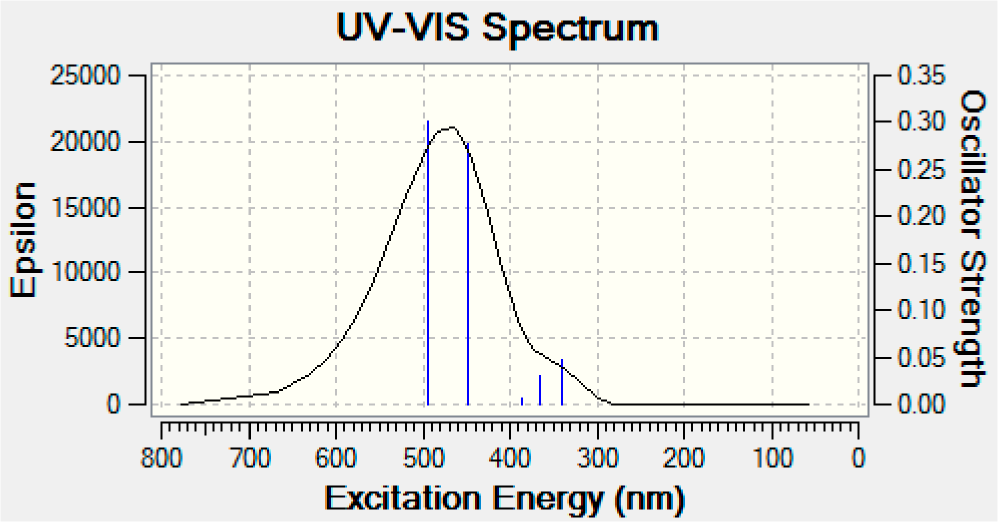

Figure S50 Measured (top) and calculated (bottom) UV-Vis spectra of the compound **(4b)**

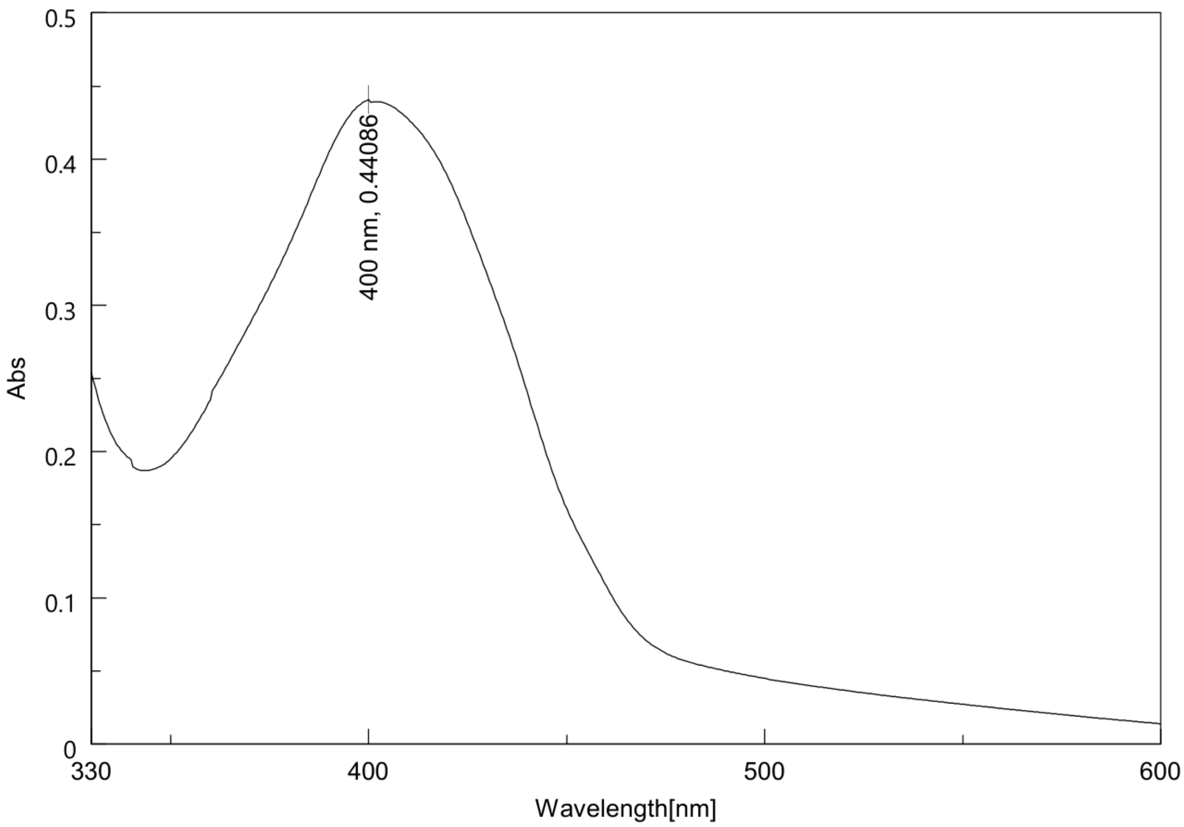

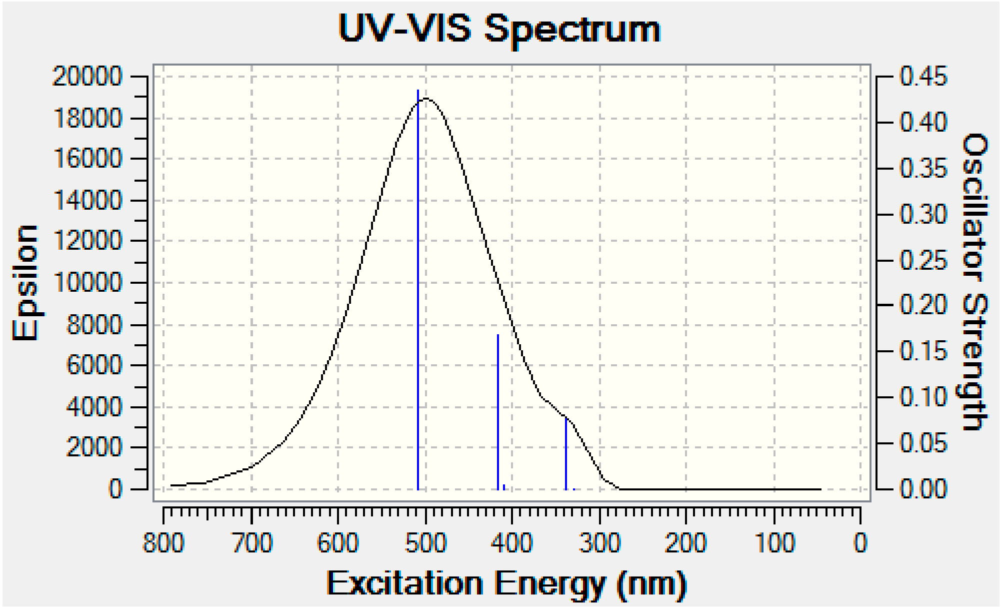

Figure S51 Measured (top) and calculated (bottom) UV-Vis spectra of the compound **(5)**

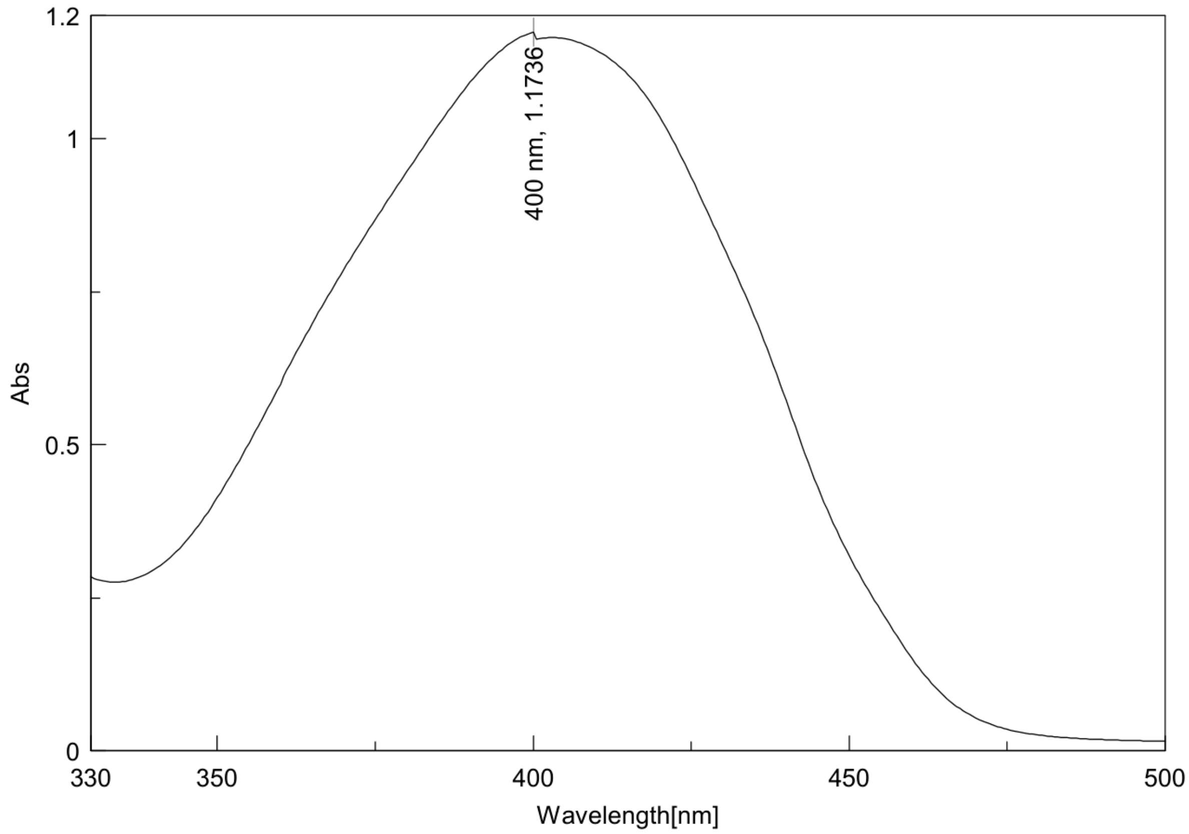

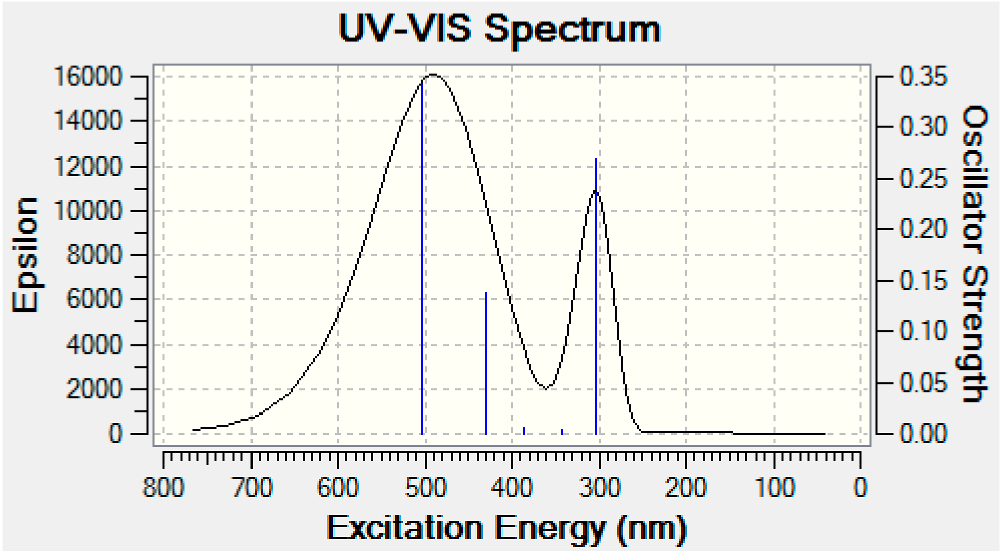

Figure S52 Measured (top) and calculated (bottom) UV-Vis spectra of the compound **(6)**

Emission spectra for compounds (6) and (4b)

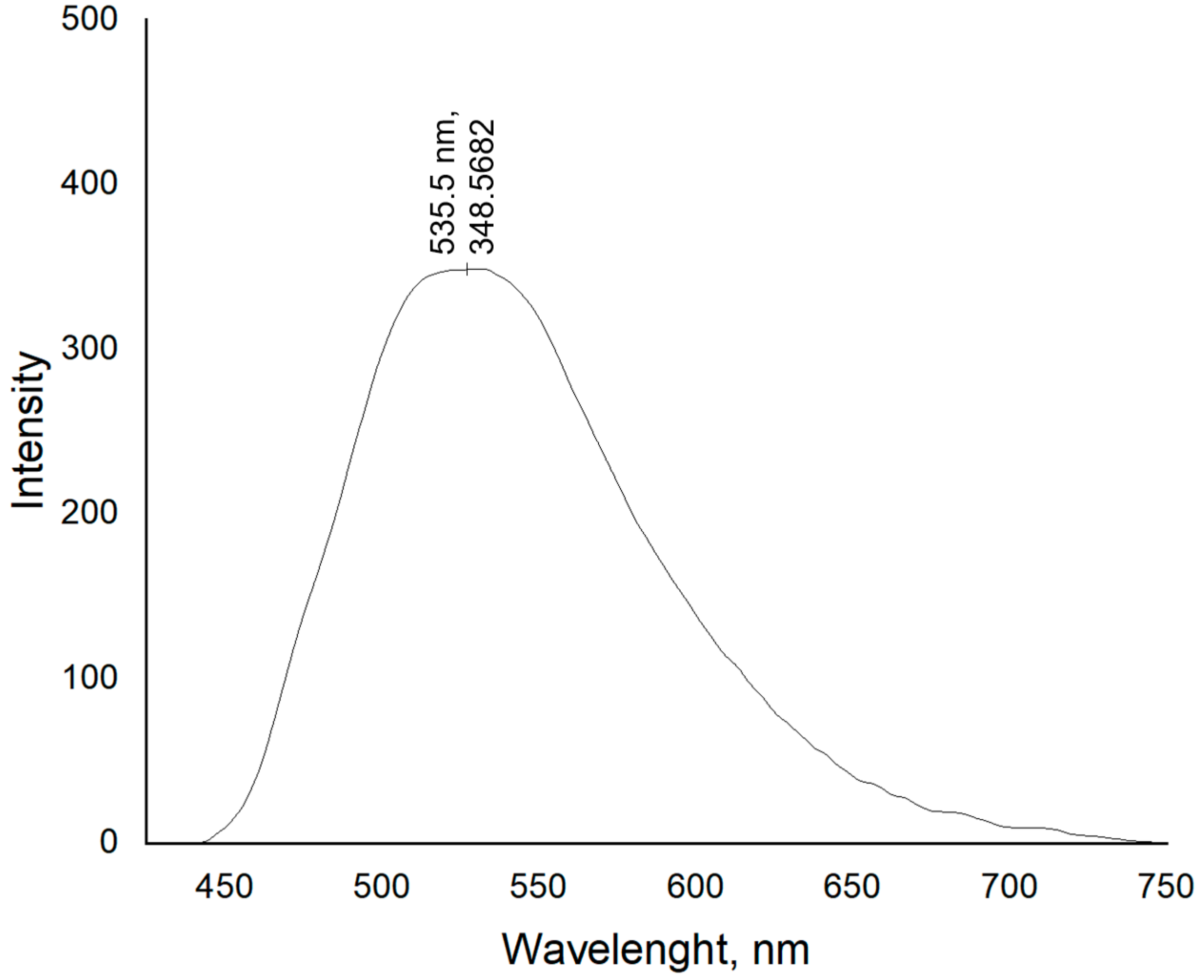

Figure S53 Emission spectrum of the compound **(6)**

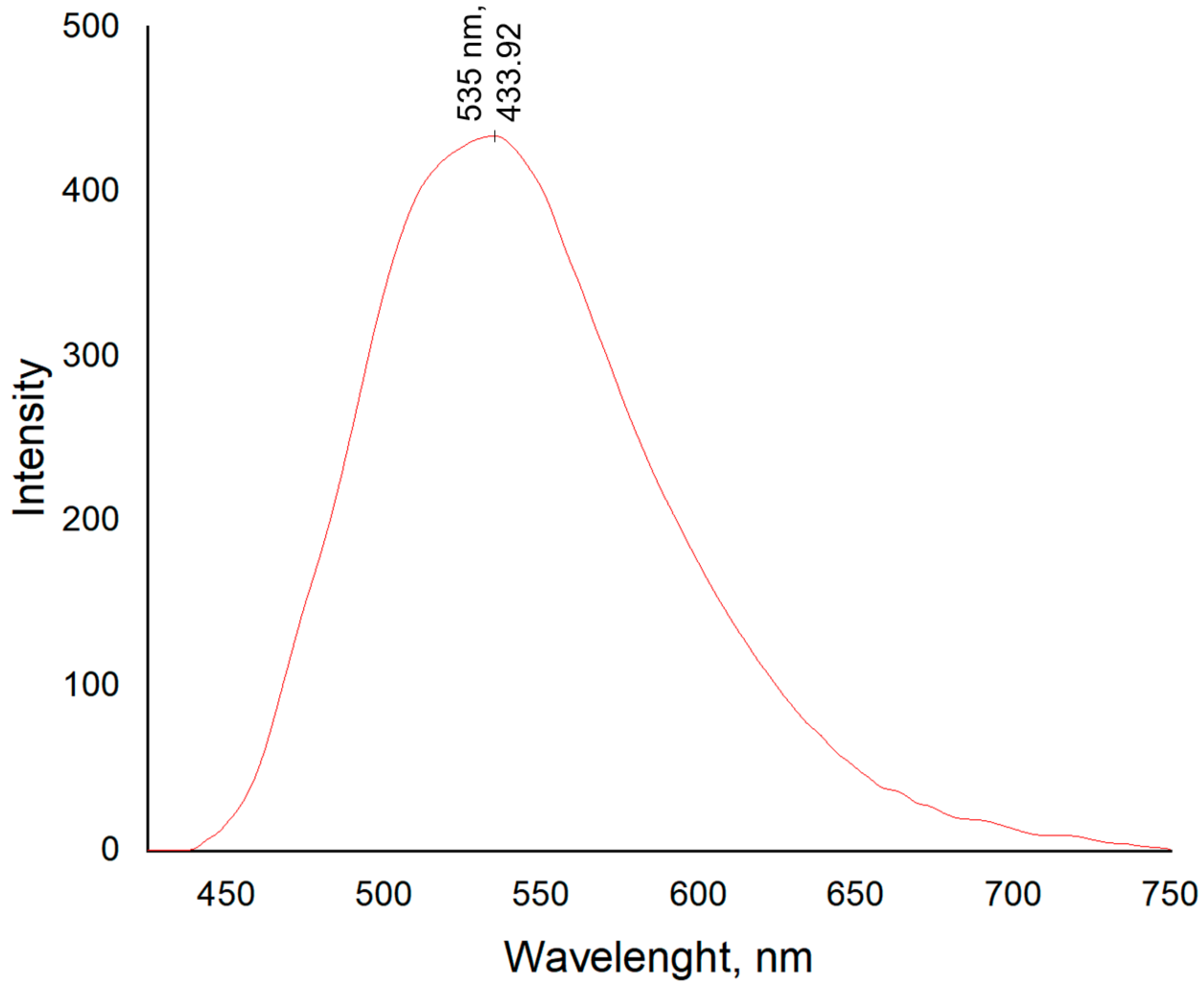

Figure S54 Emission spectrum of the compound **(4b)**

LC-HRMS data for compounds (3a), (3b), (4a), (4b), 5, 6

Compound Details

Cpd. 1: C17 H16 N4 O4

Formula	RT	Mass	Mass (Tgt)	Abund	Algorithm
C17 H16 N4 O4	1.354	340.1166	340.1172	1143919	FBF

Compound Spectra (overlaid)

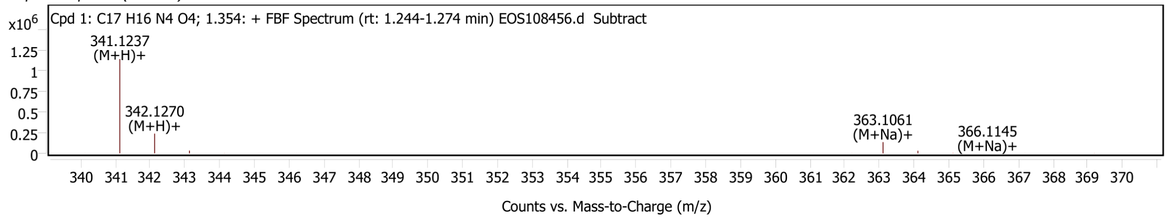

Spectrum Peaks

m/z	m/z (Calc)	Diff (ppm)	Abund	Height %	Height % (Calc)	Ion Species	Z
341.1237	341.1244	-2.16	1143919	100.00	100.00	(M+H)+	1
342.1270	342.1274	-1.03	238359	20.84	20.20	(M+H)+	1
343.1300	343.1298	0.64	32350	2.83	2.76	(M+H)+	1
363.1061	363.1064	-0.79	135032	100.00	100.00	(M+Na)+	1
364.1100	364.1093	1.98	30053	22.26	20.18	(M+Na)+	1
366.1145	366.1142	0.80	312	0.23	0.28	(M+Na)+	1

Sample Chromatograms

RT	Area Sum %	Title
1.488	100.00	UIB1 - A:Analog In (user-defined unit) EOS108456.d

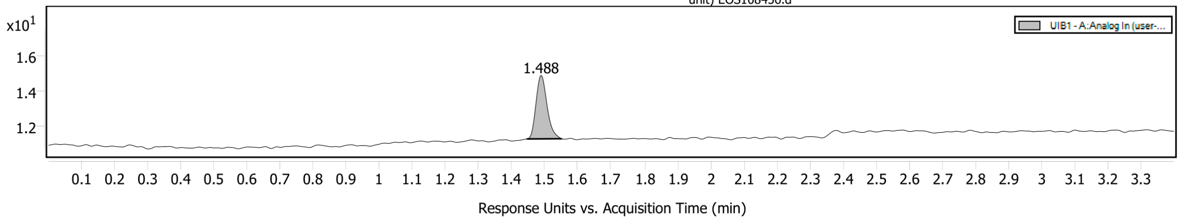

RT	Area Sum %	Title
1.433	100.00	DAD1 - A:Sig=230,4 Ref=360,100 EOS108456.d

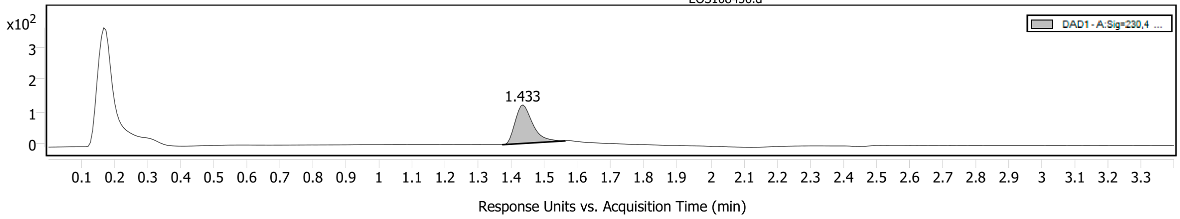

Figure S55 LC-HRMS data for compound **(3a)**

Compound Details

Cpd. 1: C19 H14 N4 O2

Formula	RT	Mass	Mass (Tgt)	Abund	Algorithm
C19 H14 N4 O2	1.514	330.1111	330.1117	822520	FBF

Compound Spectra (overlaid)

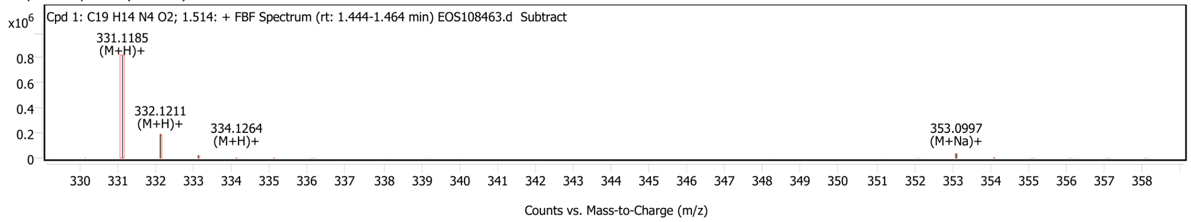

Spectrum Peaks

m/z	m/z (Calc)	Diff (ppm)	Abund	Height %	Height % (Calc)	Ion Species	Z
331.1185	331.1190	-1.23	822520	100.00	100.00	(M+H)+	1
332.1211	332.1219	-2.41	194636	23.66	22.26	(M+H)+	1
333.1233	333.1246	-3.87	23253	2.83	2.77	(M+H)+	1
334.1264	334.1272	-2.29	2479	0.30	0.25	(M+H)+	1
335.1258	335.1297	-11.74	152	0.02	0.02	(M+H)+	1
353.0997	353.1009	-3.29	36206	100.00	100.00	(M+Na)+	1
354.1026	354.1039	-3.57	7763	21.44	22.25	(M+Na)+	1

Sample Chromatograms

Chromatogram Peaks

RT	Area Sum %	Area
1.635	100	1957.69551821855

Chromatogram Peaks

RT	Area Sum %	Area
1.689	100	165.949677178003

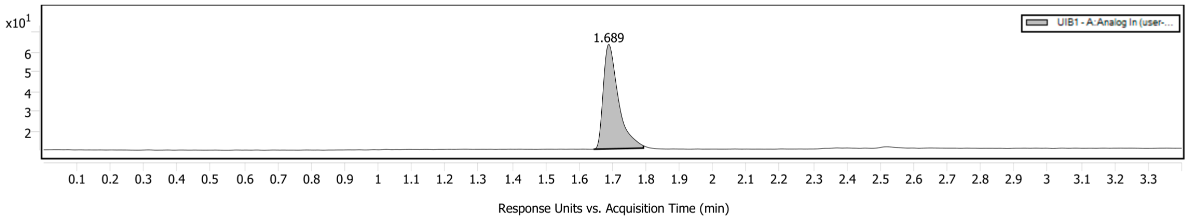

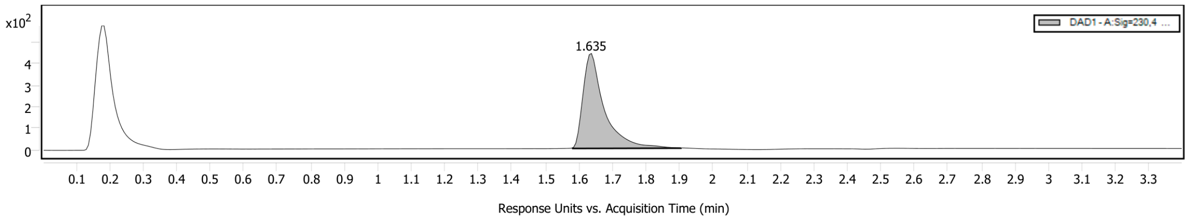

Figure S56 LC-HRMS data for compound **(3b)**

Compound Details

Cpd. 1: C17 H16 N4 O4

Formula	RT	Mass	Mass (Tgt)	Abund	Algorithm
C17 H16 N4 O4	1.354	340.1166	340.1172	1143919	FBF

Compound Spectra (overlaid)

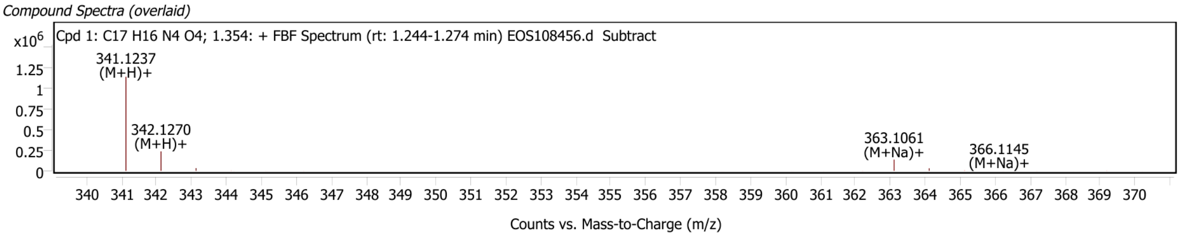

Spectrum Peaks

m/z	m/z (Calc)	Diff (ppm)	Abund	Height %	Height % (Calc)	Ion Species	Z
341.1237	341.1244	-2.16	1143919	100.00	100.00	(M+H)+	1
342.1270	342.1274	-1.03	238359	20.84	20.20	(M+H)+	1
343.1300	343.1298	0.64	32350	2.83	2.76	(M+H)+	1
363.1061	363.1064	-0.79	135032	100.00	100.00	(M+Na)+	1
364.1100	364.1093	1.98	30053	22.26	20.18	(M+Na)+	1
366.1145	366.1142	0.80	312	0.23	0.28	(M+Na)+	1

Sample Chromatograms

RT	Area Sum %	Title
1.488	100.00	UIB1 - A:Analog In (user-defined unit) EOS108456.d

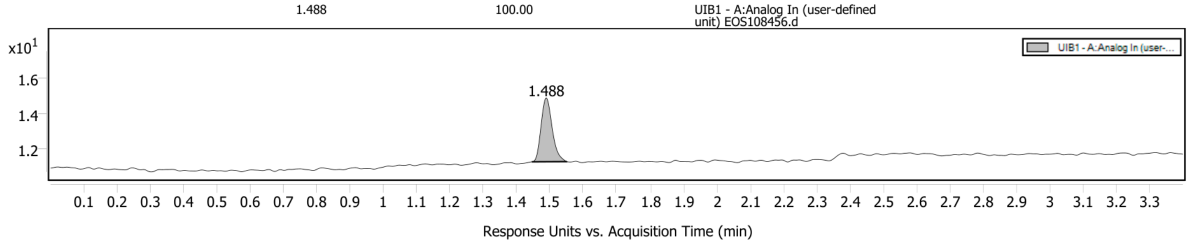

RT	Area Sum %	Title
1.433	100.00	DAD1 - A:Sig=230,4 Ref=360,100 EOS108456.d

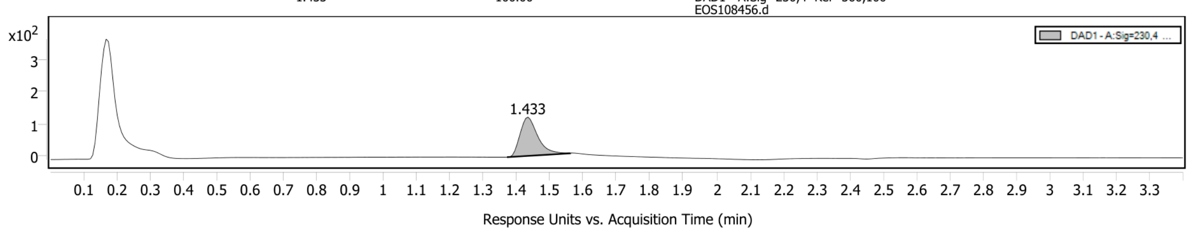

Figure S57 LC-HRMS data for compound **(4a)**

Compound Details

Cpd. 1: C19 H12 N4 O

Formula	RT	Mass	Mass (Tgt)	Abund	Algorithm
C19 H12 N4 O	1.762	312.1008	312.1011	320412	FBF

Compound Spectra (overlaid)

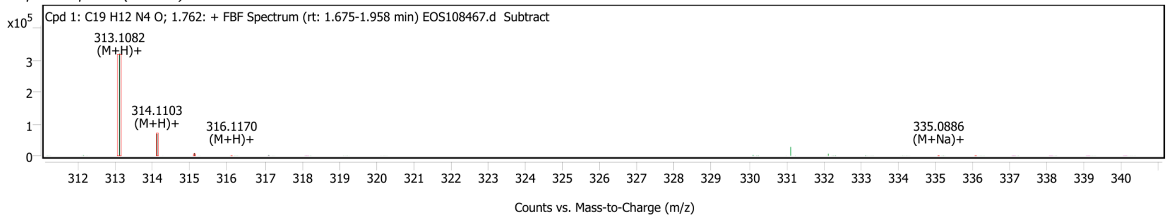

Spectrum Peaks

m/z	m/z (Calc)	Diff (ppm)	Abund	Height %	Height % (Calc)	Ion Species	Z
313.1082	313.1084	-0.64	320412	100.00	100.00	(M+H)+	1
314.1103	314.1113	-3.40	68845	21.49	22.20	(M+H)+	1
315.1137	315.1142	-1.35	8902	2.78	2.56	(M+H)+	1
316.1170	316.1168	0.57	722	0.23	0.20	(M+H)+	1
335.0886	335.0903	-5.19	818	100.00	100.00	(M+Na)+	1
336.0867	336.0933	-19.64	209	25.55	22.19	(M+Na)+	1

Sample Chromatograms

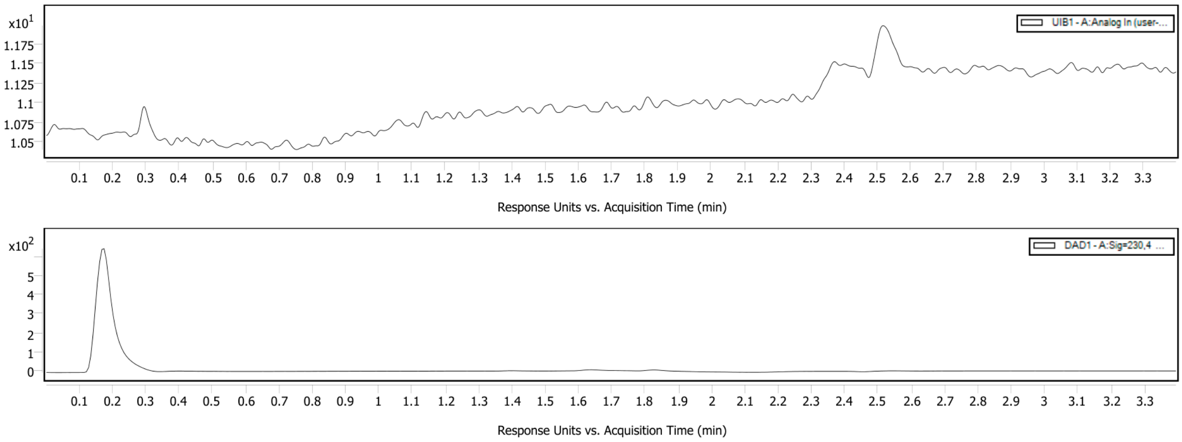

Figure S58 LC-HRMS data for compound **(4b)**

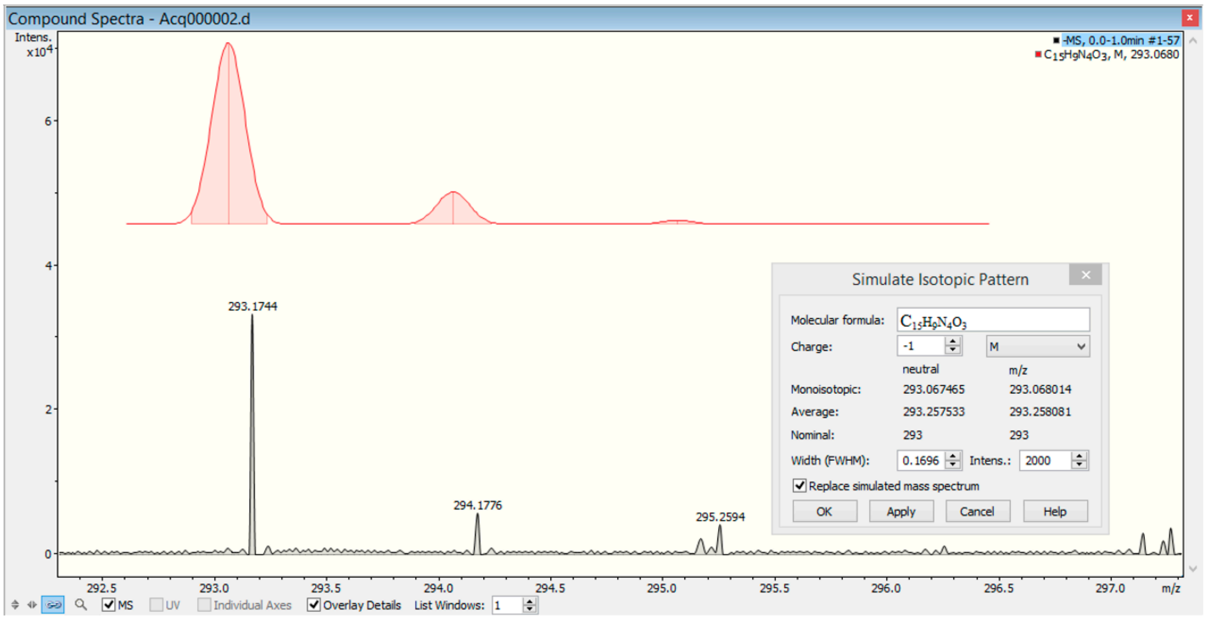

Figure 59 HRMS spectrum of compound **(5)**

Compound Details

Cpd. 1: C14 H10 N4 O

Formula	RT	Mass	Mass (Tgt)	Abund	Algorithm
C14 H10 N4 O	1.238	250.0849	250.0855	767583	FBF

Compound Spectra (overlaid)

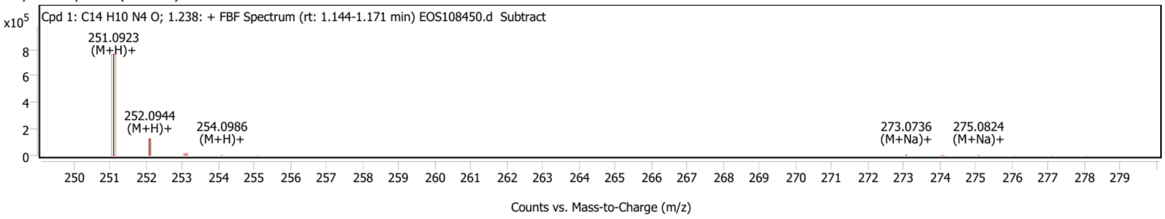

Spectrum Peaks

m/z	m/z (Calc)	Diff (ppm)	Abund	Height %	Height % (Calc)	Ion Species	Z
251.0923	251.0927	-1.82	767583	100.00	100.00	(M+H)+	1
252.0944	252.0956	-4.65	130685	17.03	16.77	(M+H)+	1
253.0975	253.0982	-2.74	12660	1.65	1.53	(M+H)+	1
254.0986	254.1006	-8.21	760	0.10	0.10	(M+H)+	1
273.0736	273.0747	-4.00	6564	100.00	100.00	(M+Na)+	1
274.0770	274.0775	-1.72	1231	18.75	16.76	(M+Na)+	1
275.0824	275.0801	8.26	277	4.22	1.52	(M+Na)+	1

Sample Chromatograms

Chromatogram Peaks

RT	Area Sum %	Area
1.331	100	317.007706433776

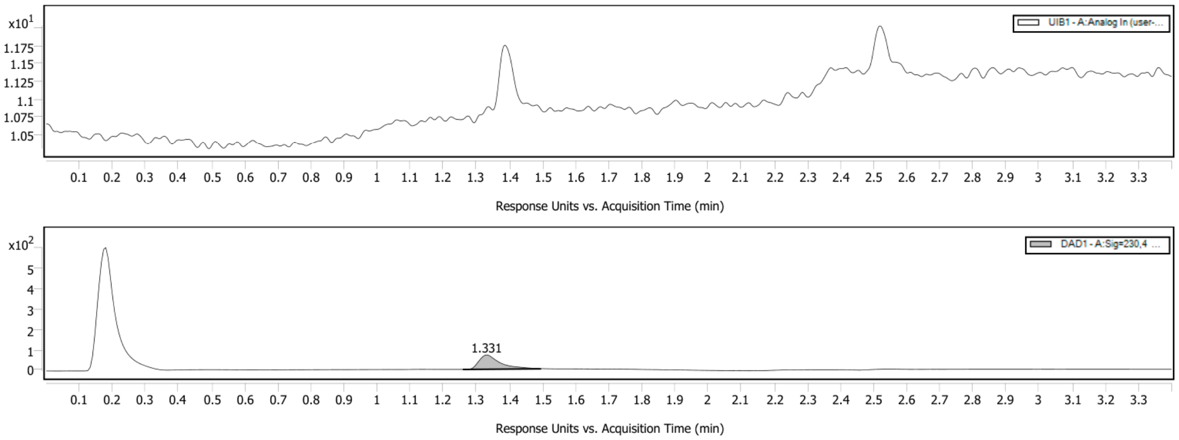

Figure S60 LC-HRMS data for compound **(6)**

Biological activity evaluation data for compounds (3a), (3b), (4a), (4b), 5, 6

Compound	Candida albicans Conc./ % inhibition/ Result	Aspergillus fumigatus Conc./ % inhibition/ Result	Candida Auris Conc./ % inhibition/ Result	Enterococcus faecalis ATCC Conc./ % inhibition/ Result	Staphylococcus aureus Conc./ % inhibition/ Result	Pseudomonas aeruginosa Conc./ % growth inhibition/ Result	Escherichia coli Conc./ % growth inhibition / Result	Klebsiella pneumoniae Conc./ % inhibition/ Result	Acinetobacter baumannii Conc./ % inhibition/ Result	Cell line permanent Hep-G2 Conc./ % inhibition / Result
**3a**	50.0 µM/ 4.55/ inactive	50.0 µM/ 5.38/ inactive	50.0 µM/ 0.96/ inactive	50.0 µM/ -7.04/ inactive	50.0 µM/ -6.08/ inactive	50.0 µM/ 12.70/ inactive	50.0 µM/ -13.99 / inactive	50.0 µM/ -32.00 / inactive	50.0 µM/ -22.02 / inactive	10000.0 nM/ 3.5h 22.50 % inactive
**4a**	50.0 µM/ -0.78/ inactive	50.0 µM/ -14.14/ inactive	50.0 µM/ 6.24/ inactive	50.0 µM/ -1.84/ inactive	50.0 µM/ 5.32/ inactive	50.0 µM/ -2.82/ inactive	50.0 µM/ -16.42 / inactive	50.0 µM/ -19.00 / inactive	50.0 µM/ -10.33 / inactive	10000.0 nM/ 3.5h 3.60 % inactive
**3b**	50.0 µM/ 8.88/ inactive	50.0 µM/ -9.79/ inactive	50.0 µM/ 34.00/ inactive	50.0 µM/ 6.85/ inactive	50.0 µM/ -8.47/ inactive	50.0 µM/ -10.40/ inactive	50.0 µM/ -12.23 / inactive	50.0 µM/ -12.00 / inactive	50.0 µM/ -15.05 / inactive	10000.0 nM/ 3.5h 62.60 % active
**4b**	50.0 µM/ 2.05/ inactive	50.0 µM/ -16.18/ inactive	50.0 µM/ 7.67/ inactive	50.0 µM/ 1.41/ inactive	50.0 µM/ 8.10/ inactive	50.0 µM/ -5.30/ inactive	50.0 µM/ -6.73 / inactive	50.0 µM/ 14.00 / inactive	50.0 µM/ -10.33 / inactive	10000.0 nM/ 3.5h 11.20 % inactive
**5**	50.0 µM/ -1.88/ inactive	50.0 µM/ -15.78/ inactive	50.0 µM/ 3.31/ inactive	50.0 µM/ -1.43/ inactive	50.0 µM/ 0.35/ inactive	50.0 µM/ -7.33/ inactive	50.0 µM/ -9.66 / inactive	50.0 µM/ -12.00 / inactive	50.0 µM/ -20.61 / inactive	10000.0 nM/ 3.5h 7.60 % inactive
**6**	50.0 µM/ 14.95/ inactive	50.0 µM/ -6.41/ inactive	50.0 µM/ 2.62/ inactive	50.0 µM/ 0.09/ inactive	50.0 µM/ -6.69/ inactive	50.0 µM/ 18.70/ inactive	50.0 µM/ -11.13 / inactive	50.0 µM/ -38.00 / inactive	50.0 µM/ 0.37 / inactive	10000.0 nM/ 3.5h 11.60 %

										inactive

1. **Candida albicans ATCC 64124 Anti-Fungal Assay**

Screening of the 3rd batch of the ACADEMIC Library (1408 compounds) for 1 concentration (50 µM) with 2 replicates against C. albicans ATCC64124, according to "N8: Anti-Bacterial and Anti-Fungal Assays Bioprofiling Handbook" and following the protocols optimized during the validation step indicated in that proposal (Under the frame of the EU-OS bioprofiling tender process), to determine the antibacterial properties of these compounds against this human pathogen. Briefly, compounds were tested in a liquid growth medium dispensed in 384 well plates inoculated with a standardized fungal suspension and after overnight incubation at 37ºC the plates were examined for bacterial growth as absorbance readout. The Genedata Screener software (Genedata, Inc., Basel, Switzerland) was used to process and analyze all the data from the screen. The reproducibility and sensitivity were supported by the statistical values derived from all the experiments performed. The activity of the compounds was expressed as a percentage of growth inhibition where 100% represented the inhibition growth of the target microorganism and 0% represented the total growth of target microorganism on the assay.

Assay setup

Assay stage: primary assay

Bioassay: cell growth assay

Bioassay type: functional phenotypic

Bioassay setting: in vitro

BAO tags: high throughput screening, research institute, researcher, screening lab investigator, antifungal drug

Screening site: Fundación MEDINA - Screening and target validation, Microbiology and Chemistry

Detection method

Physical detection method: absorbance

Detection instrument: EnVision Multilabel Reader

Target type: Organism

Activities

Concentration unit: micromolar

Activity determination method: "Value" column is the activity of the compound calculated as an average of the percentage of inhibition. "Active" is defined as a value equal or greater than 70 (≥ 70). "Inconclusive" is defined as a value less than 70 (< 70) and equal or greater than 50 (≥ 50). "Inactive" is defined as a value less than 50 (< 50). A column with the calculated value of the standard deviation has been added to roughly estimate the reliability of the data .

2. Aspergillus fumigatus ATCC 46645 Anti-Fungal Assay

Screening of the 3rd batch of the ACADEMIC Library (1408 compounds) for 1 concentration (50 µM) with 2 replicates against A. fumigatus ATCC46645, according to "N8: Anti-Bacterial and Anti-Fungal Assays Bioprofiling Handbook" and following the protocols optimized during the validation step

indicated in that proposal (Under the frame of the EU-OS bioprofiling tender process), to determine the antifungal properties of these compounds against this human pathogen. Briefly, compounds were tested in a liquid growth medium dispensed in 384 well plates inoculated with a standardized fungal suspension and after overnight incubation at 37ºC the plates were examined for fungal growth as fluorescence readout. The Genedata Screener software (Genedata, Inc., Basel, Switzerland) was used to process and analyze all the data from the screen. The reproducibility and sensitivity were supported by the statistical values derived from all the experiments performed. The activity of the compounds was expressed as a percentage of growth inhibition where 100% represented the inhibition growth of the target microorganism and 0% represented the total growth of target microorganism on the assay.

Assay setup

Assay stage: primary assay

Bioassay: cell growth assay

Bioassay type: functional phenotypic

Bioassay setting: in vitro

BAO tags: high throughput screening, research institute, researcher, screening lab investigator, antifungal drug

Screening site: Fundación MEDINA - Screening and target validation, Microbiology and Chemistry

Detection method

Physical detection method: absorbance

Detection instrument: EnVision Multilabel Reader

Target type: Organism

Activities

Concentration unit: micromolar

Activity determination method: "Value" column is the activity of the compound calculated as an average of the percentage of inhibition. "Active" is defined as a value equal or greater than 70 (≥ 70). "Inconclusive" is defined as a value less than 70 (< 70) and equal or greater than 50 (≥ 50). "Inactive" is defined as a value less than 50 (< 50). A column with the calculated value of the standard deviation has been added to roughly estimate the reliability of the data .

3. Candida auris DSM21092 Anti-Fungal Assay

Screening of the 3 rd batch of the Academic Library (1408 compounds) for 1 concentration (50 µM) with 2 replicates against C. auris DSM21092, according to "N8: Anti-Bacterial and Anti-Fungal Assays Bioprofiling Handbook" and following the protocols optimized during the validation step indicated in that proposal (Under the frame of the EU-OS bioprofiling tender process), to determine the antifungal properties of these compounds against this human pathogen. Briefly, compounds were tested in a liquid growth medium dispensed in 384 well plates inoculated with a standardized fungal suspension and after overnight incubation at 37ºC the plates were examined for fungal growth as absorbance readout. The Genedata Screener software (Genedata, Inc., Basel, Switzerland) was used to process and analyze all the data from the screen. The reproducibility and sensitivity were supported

by the statistical values derived from all the experiments performed. The activity of the compounds was expressed as a percentage of growth inhibition where 100% represented the inhibition growth of the target microorganism and 0% represented the total growth of target microorganism on the assay.

Assay setup

Assay stage: primary assay

Bioassay: cell growth assay

Bioassay type: functional phenotypic

Bioassay setting: in vitro

BAO tags: high throughput screening, research institute, researcher, screening lab investigator, antifungal drug

Screening site: Fundación MEDINA - Screening and target validation, Microbiology and Chemistry

Detection method

Physical detection method: absorbance

Detection instrument: EnVision Multilabel Reader

Target type: Organism

Activities

Concentration unit: micromolar

Activity determination method: "Value" column is the activity of the compound calculated as an average of the percentage of inhibition. "Active" is defined as a value equal or greater than 70 (≥ 70). "Inconclusive" is defined as a value less than 70 (< 70) and equal or greater than 50 (≥ 50). "Inactive" is defined as a value less than 50 (< 50). A column with the calculated value of the standard deviation has been added to roughly estimate the reliability of the data .

4. Enterococcus faecalis ATCC 29212 Anti-Bacterial Assay

Screening of the 3 rd batch of the Academic Library (1408 compounds) for 1 concentration (50 µM) with 2 replicates against Enterococcus faecalis ATCC29212, according to "N8: Anti-Bacterial and Anti-Fungal Assays Bioprofiling Handbook" and following the protocols optimized during the validation step indicated in that proposal (Under the frame of the EU-OS bioprofiling tender process), to determine the antibacterial properties of these compounds against this human pathogen. Briefly, compounds were tested in a liquid growth medium dispensed in 384 well plates inoculated with a standardized bacterial suspension (CFU/mL) and after overnight incubation at 37ºC the plates were examined for bacterial growth as absorbance readout. The Genedata Screener software (Genedata, Inc., Basel, Switzerland) was used to process and analyze all the data from the screen. The reproducibility and sensitivity were supported by the statistical values derived from all the experiments performed. The activity of the compounds was expressed as a percentage of growth inhibition where 100% represented the inhibition growth of the target microorganism and 0% represented the total growth of target microorganism on the assay.

Assay setup

Assay stage: primary assay

Bioassay: cell growth assay

Bioassay type: functional phenotypic

Bioassay setting: in vitro

BAO tags: high throughput screening, research institute, researcher, screening lab investigator, antifungal drug

Screening site: Fundación MEDINA - Screening and target validation, Microbiology and Chemistry

Detection method

Physical detection method: absorbance

Detection instrument: EnVision Multilabel Reader

Target type: Organism

Activities

Concentration unit: micromolar

Activity determination method: "Value" column is the activity of the compound calculated as an average of the percentage of inhibition. "Active" is defined as a value equal or greater than 70 (≥ 70). "Inconclusive" is defined as a value less than 70 (< 70) and equal or greater than 50 (≥ 50). "Inactive" is defined as a value less than 50 (< 50). A column with the calculated value of the standard deviation has been added to roughly estimate the reliability of the data .

5. Staphylococcus aureus, MSSA ATCC 29213 Anti-Bacterial Assay

Screening of the 3rd batch of the Academic Library (1408 compounds) for 1 concentration (50 µM) with 2 replicates against methicillin-susceptible Staphylococcus aureus ATCC29213, according to "N8: Anti-Bacterial and Anti-Fungal Assays Bioprofiling Handbook" and following the protocols optimized during the validation step indicated in that proposal (Under the frame of the EU-OS bioprofiling tender process), to determine the antibacterial properties of these compounds against this human pathogen. Briefly, compounds were tested in a liquid growth medium dispensed in 384 well plates inoculated with a standardized bacterial suspension (CFU/mL) and after overnight incubation at 37ºC the plates were examined for bacterial growth as absorbance readout. The Genedata Screener software (Genedata, Inc., Basel, Switzerland) was used to process and analyze all the data from the screen. The reproducibility and sensitivity were supported by the statistical values derived from all the experiments performed. The activity of the compounds was expressed as a percentage of growth inhibition where 100% represented the inhibition growth of the target microorganism and 0% represented the total growth of target microorganism on the assay.

Assay setup

Assay stage: primary assay

Bioassay: cell growth assay

Bioassay type: functional phenotypic

Bioassay setting: in vitro

BAO tags: high throughput screening, research institute, researcher, screening lab investigator, antifungal drug

Screening site: Fundación MEDINA - Screening and target validation, Microbiology and Chemistry

Detection method

Physical detection method: absorbance

Detection instrument: EnVision Multilabel Reader

Target type: Organism

Activities

Concentration unit: micromolar

Activity determination method: "Value" column is the activity of the compound calculated as an average of the percentage of inhibition. "Active" is defined as a value equal or greater than 70 (≥ 70). "Inconclusive" is defined as a value less than 70 (< 70) and equal or greater than 50 (≥ 50). "Inactive" is defined as a value less than 50 (< 50). A column with the calculated value of the standard deviation has been added to roughly estimate the reliability of the data .

6. Growth inhibition Pseudomonas aeruginosa

Compound plates were prepared using the acoustic dispenser Echo 550 to transfer 250 nL into each well of a 384 well plate. Assay volume was 60 µL of the bacterial suspension, which was prepared from an overnight culture. Plates were incubated at 37°C for 24h. Growth was followed via determination of the optical density at 600 nm. The values resulting from 24h were analysed.

Assay setup

Assay stage: primary assay

Bioassay: cell growth assay

Bioassay type: functional phenotypic

Bioassay setting: in vitro

Screening site: Helmholtz Centre for Infection Research - Department of Chemical Biology

Detection method

Physical detection method: absorbance

Detection instrument: Synergy HT Multi-Mode Reader (BioTek)

Target type: Organism

Activities

Concentration unit: micromolar

Activity determination method: Optical density was normalized as growth inhibition with respect to control samples. Compounds are active, when they resulted in more than 50% growth inhibition.

7. Inhibition of Escherichia coli

The growth inhibitory potential of compounds for E.coli ATCC 25922 is detected via the turbidity of the solutions at 600 nm (OD600).

Assay setup

Assay stage: primary assay

Bioassay: cell growth assay

Bioassay type: functional phenotypic

Bioassay setting: in vitro

Screening site: Helmholtz Centre for Infection Research - Department of Chemical Biology

Detection method

Physical detection method: transmittance

Detection instrument: Synergy HT Multi-Mode Reader (BioTek)

Target type: Organism

Activities

Concentration unit: micromolar

Activity determination method: Data are normalised with respect to controls as % growth inhibition. Compounds are defined as active, when the growth is inhibited to more than 50%.

8. Growth inhibition of Klebsiella pneumonia

Klebsiella pneumonia, DSM681 = ATCC 10031, was cultivated in MHB in 384 well plates. 250nL of 10 mM solutions of each compound in DMSO were transferred with the acoustic dispenser Echo 550 to each well. The cultivation volume was 60 µL, plates were incubated at 37°C for 24h. The optical density at 600nm was determined and used as measure for bacterial growth. Data from control wells with medium only were used as 100% inhibition, and data from control wells with bacteria only were set as 0% growth inhibition. 50% growth inhibition was chosen as threshold to identify active compounds.

Assay setup

Assay stage: primary assay

Bioassay: cell growth assay

Bioassay type: functional phenotypic

Bioassay setting: in vitro

Screening site: Helmholtz Centre for Infection Research - Department of Chemical Biology

Detection method

Physical detection method: absorbance

Detection instrument: Synergy HT Multi-Mode Reader (BioTek)

Target type: Organism

Activities

Concentration unit: micromolar

Activity determination method: primary data are single point measurements, and activity is defined as at least 50% growth inhibition after 24h.

9. Growth inhibition of Acinetobacter baumanii

Acinetobacter baumanii, DSM30007, was cultivated in TSY in 384 well plates. 250nL of 10 mM solutions of each compound in DMSO were transferred with the acoustic dispenser Echo 550 to each well. The cultivation volume was 60 µL, plates were incubated at 37°C for 24h. The optical density at 600nm was determined and used as measure for bacterial growth. Data from control wells with medium only were used as 100% inhibition, and data from control wells with bacteria only were set as 0% growth inhibition. 50% growth inhibition was chosen as threshold to identify active compounds.

Assay setup

Assay stage: primary assay

Bioassay: cell growth assay

Bioassay type: functional phenotypic

Bioassay setting: in vitro

Screening site: Helmholtz Centre for Infection Research - Department of Chemical Biology

Detection method

Physical detection method: absorbance

Detection instrument: Synergy HT Multi-Mode Reader (BioTek)

Target type: Organism

Activities

Concentration unit: micromolar

Activity determination method: primary data are single point measurements, and activity is defined as at least 50% growth inhibition after 24h..

10. Cell viability ATP quantification assay with HepG2 cells

Plate type: 384-well plate Corning 3764 Black clear bottom. 750 cells /well seeded into the plate. Cell culture medium: DMEM (1.0 g/L glucose, without L-Glutamine, Lonza 12-707F), 10% FBS (Gibco 10270106), 2 mM L-glutamine (Lonza BE17-605E), 2% Pen/Strep (Lonza DE17-602E), + 1% NEAA (Gibco 11140-035) 48 hour incubation time. Cell viability ATP quantification assay with HepG2 cells. Assay kit: CellTiter-Glo® 2.0 Cell Viability Assay - Promega Corporation. 15uL CellTiter-Glo/well added. Screening concentration was 10 uM. Luminescence was detected using Pherastar FS reader. Data was normalized to the low (10 uM Sepantronium bromide - YM155) and high controls (25 nL/well Dimethyl Sulfoxide - DMSO) to generate percent inhibition values. The percent inhibition values uploaded.

Assay setup

Assay stage: primary assay

Bioassay: cell viability ATP quantitation assay

Bioassay type: functional

Bioassay setting: in vitro

Assay organism: Homo sapiens

BAO tags: time unit, hour, screening concentration, normalized data

Screening site: Institute for Molecular Medicine Finland - High Throughput Biomedicine Unit

Detection method

Physical detection method: luminescence method

Detection instrument: BMG PHERAstar FS plate reader (BMG Labtech, Cary NC)

Target

Target type: Cell line

Cell-line type: Permanent

Activities

Concentration unit: nanomolar

Activity determination method: The compound was selected for validation if the average Percent Inhibition value (at 10 uM) was equal or greater than 30% in the primary screening.

10. Cell viability ATP quantification assay with HepG2 cells

Validation screen with 207 compounds that showed at least 30% Inhibition in the primary screen. Plate type: 384-well plate Corning 3764 Black clear bottom. 750 cells /well seeded into the plate. Cell culture medium: DMEM (1.0 g/L glucose, without L-Glutamine, Lonza 12-707F), 10% FBS (Gibco 10270106), 2 mM L-glutamine (Lonza BE17-605E), 2% Pen/Strep (Lonza DE17-602E), + 1% NEAA (Gibco 11140-035) 48 hour incubation time. Cell viability ATP quantification assay with HepG2 cells. Assay kit: CellTiter-Glo® 2.0 Cell Viability Assay - Promega Corporation. 15uL CellTiter-Glo/well added. Compounds were tested with 8 doses in triplicates in independent experiments. Screening

concentrations were 0.001, 0.01, 0.1, 0.3, 1, 3, 10, 30, 50 uM. Luminescence was detected using Pherastar FS reader. Data was normalized to the low (10 uM Sepantronium bromide - YM155) and high controls (25 nL/well Dimethyl Sulfoxide - DMSO) to generate percent inhibition values. The percent inhibition values uploaded. Curve fitting was done using four parameter hill fitting algorithm. AUC, IC50, low and high asymptote values are reported.

Assay setup

Assay stage: confirmatory assay

Bioassay: cell viability ATP quantitation assay

Bioassay type: functional

Bioassay setting: in vitro

Assay organism: Homo sapiens

BAO tags: time unit, hour, screening concentration, normalized data

Screening site: Institute for Molecular Medicine Finland - High Throughput Biomedicine Unit

Detection method

Physical detection method: luminescence method

Detection instrument: BMG PHERAstar FS plate reader (BMG Labtech, Cary NC)

Target

Target type: Cell line

Cell-line type: Permanent

Activities

Concentration unit: nanomolar

Activity determination method: Validation screen with active compounds with average percent inhibition value (at 10 uM concentration) > 30. There were 207 compounds based on the percent inhibition values from the primary screen.

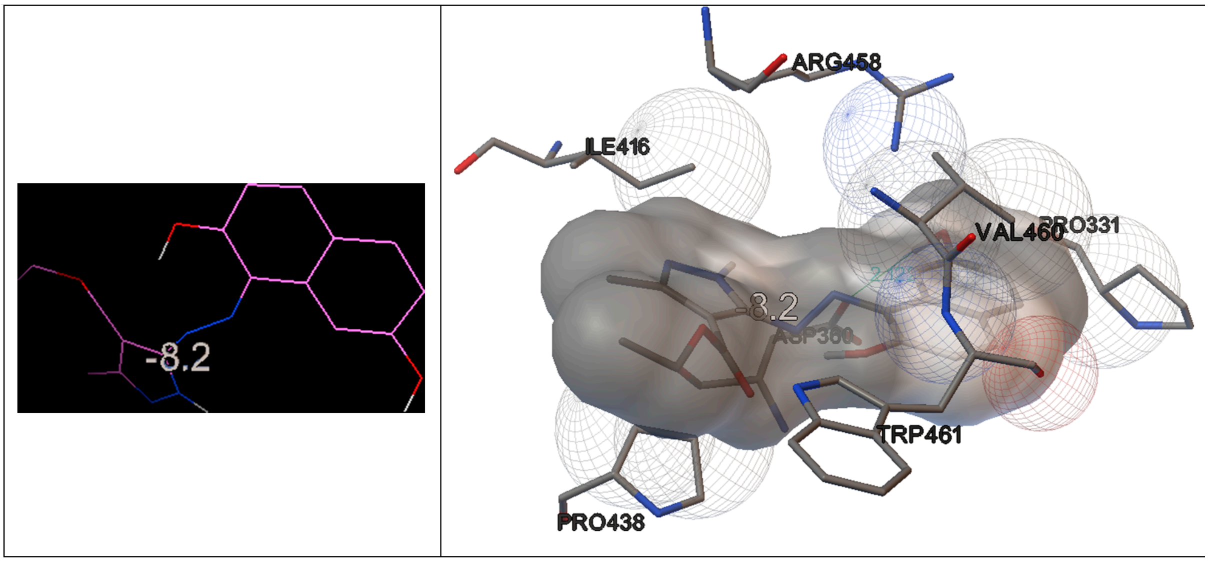

Figure S61 Best ligand **(3a)** conformation (left) and ligand **(3a)** – receptor (3GCW protein) interaction

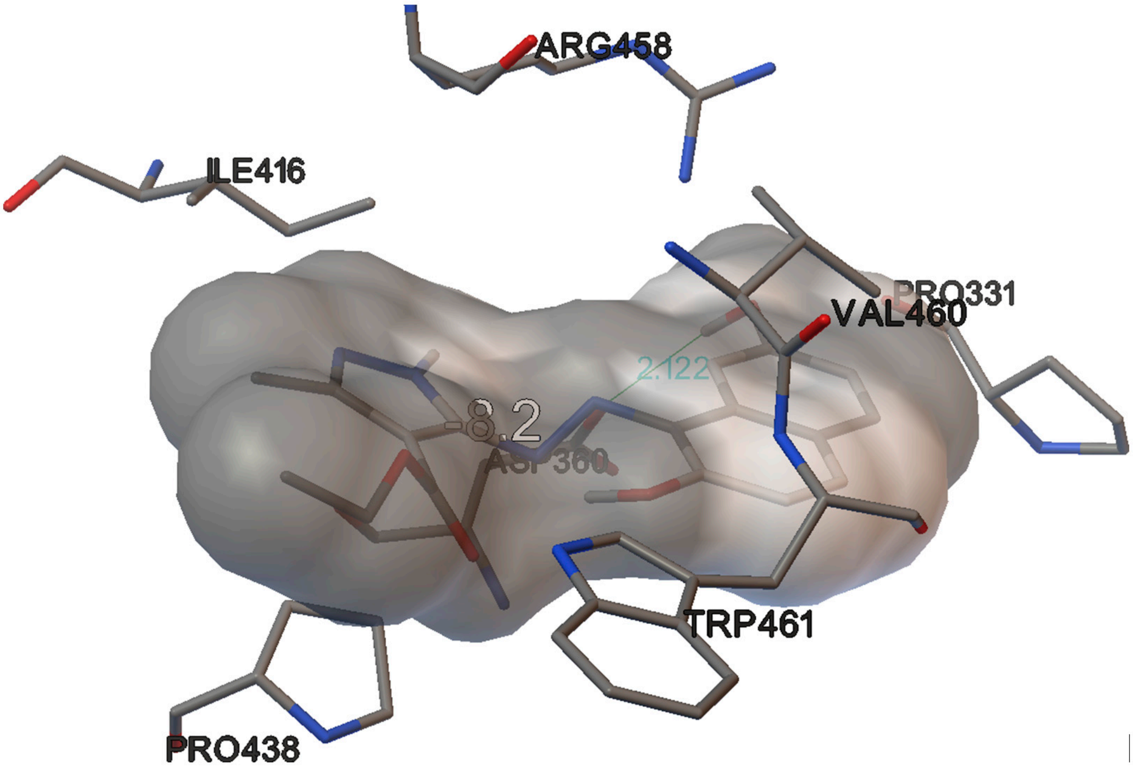

Figure S62 Depiction of the hydrogen bond established between the naphtol -OH group of compound **(3a)** and Asp360 residue

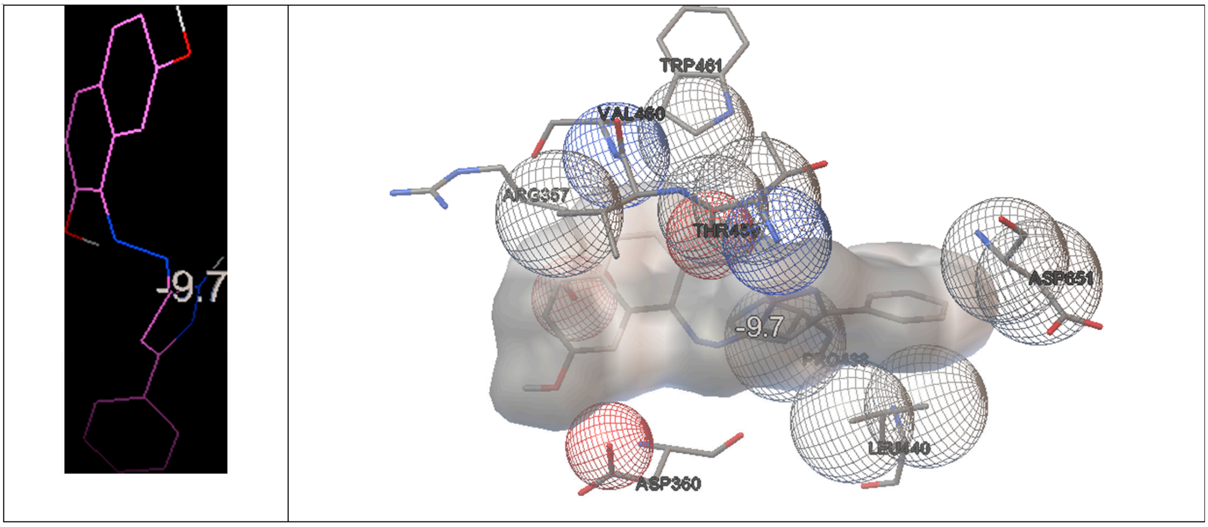

Figure S63 Best ligand **(3b)** conformation (left) and ligand **(3b)** – receptor (3GCW protein) interactions (right)

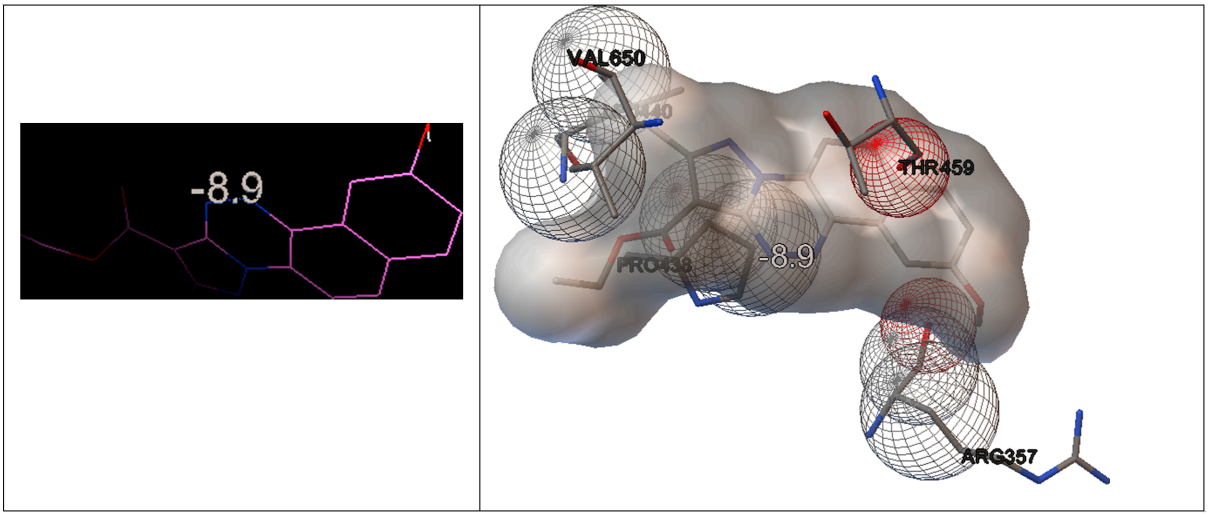

Figure S64 Best ligand **(4a)** conformation (left) and ligand **(4a)** – receptor (3GCW protein) interactions (right)

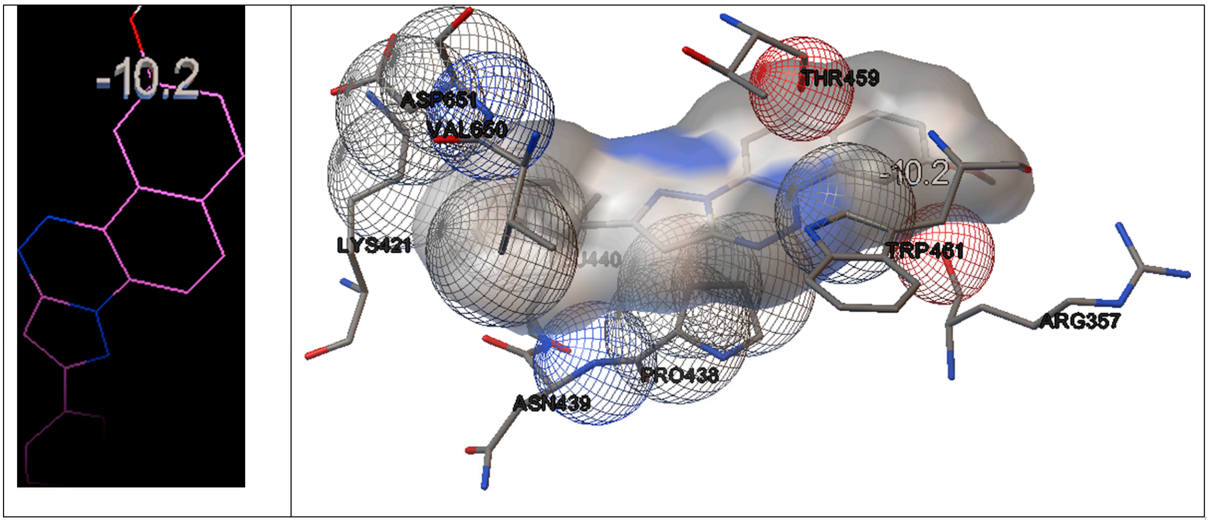

Figure S65 Best ligand **(4b)** conformation (left) and ligand **(4b)** – receptor (3GCW protein) interactions

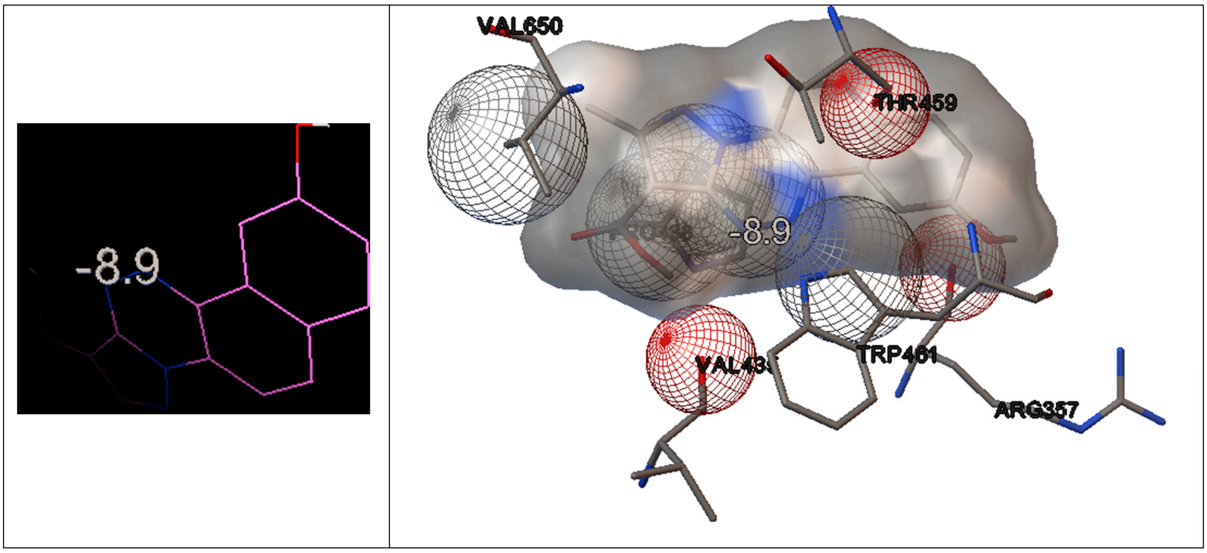

Figure S66 Best ligand **(5)** conformation (left) and ligand **(5)** – receptor (3GCW protein) interactions (right)

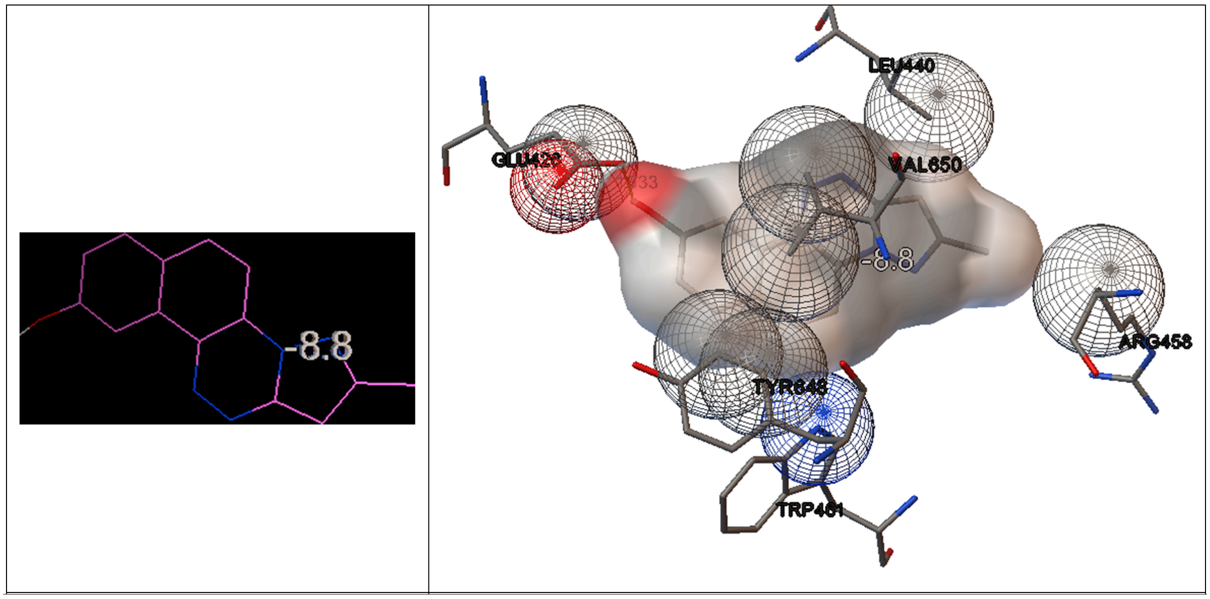

Figure S67 Best ligand **(6)** conformation (left) and ligand **(6)** – receptor (3GCW protein) interactions (right)

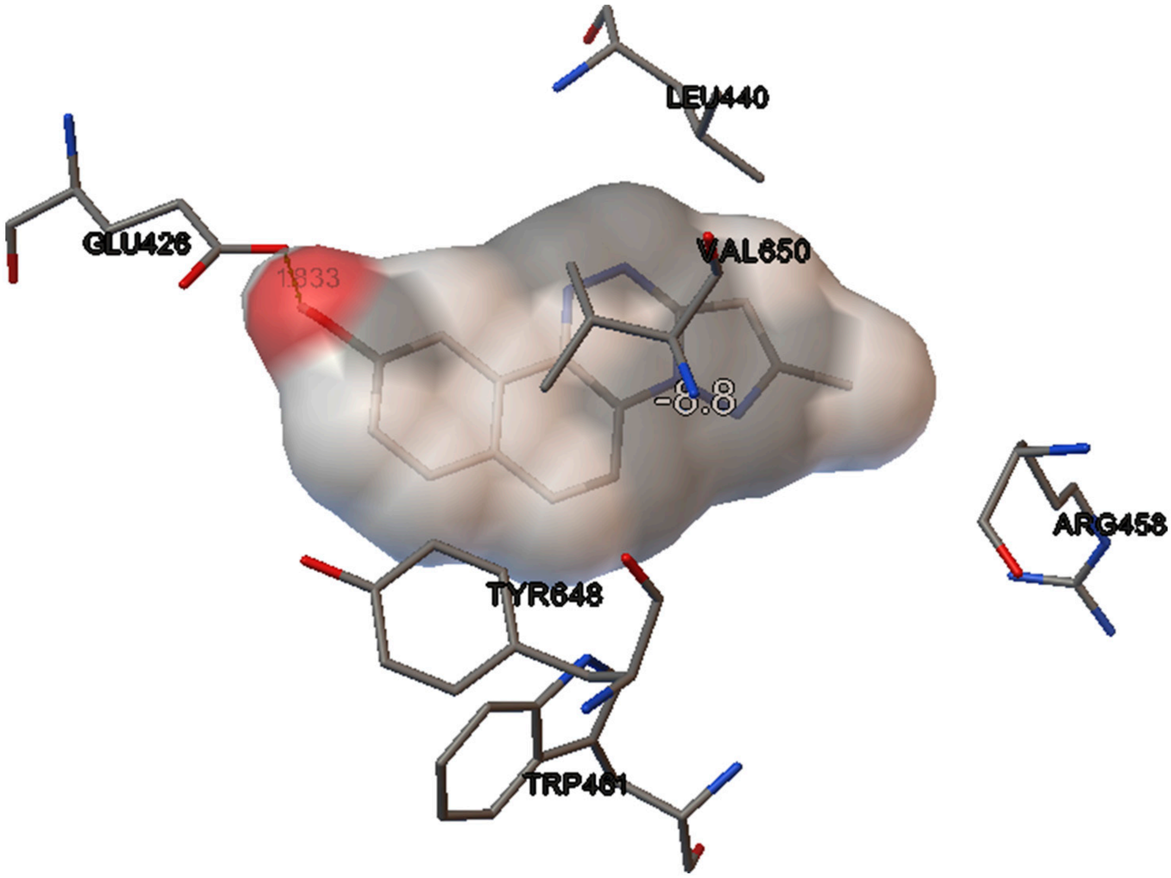

Figure S68 Depiction of the hydrogen bond established between the naphtol -OH group of compound **(6)** and Glu426 residue
